# Incidence of Herpes Zoster Infection (Shingles) Among Adults Living With and Without HIV in British Columbia, Canada: A Population-based Study

**DOI:** 10.1093/infdis/jiag118

**Published:** 2026-03-10

**Authors:** Viviane Dias Lima, Saarah Sofia Hussain, Jintong Yan, Esther Copp, Mark Hull, Michael Budu, Felipe Duailibe, Jason Trigg, Victor Leung, Peter Phillips, Silvia Guillemi, Robert S Hogg, Julio S G Montaner

**Affiliations:** Department of Epidemiology and Population Health, British Columbia Centre for Excellence in HIV/AIDS, Vancouver, British Columbia, Canada; Division of Infectious Diseases, Department of Medicine, Faculty of Medicine, University of British Columbia, Vancouver, British Columbia, Canada; College of Medicine and Health, School of Medicine, University College Cork, Cork, Ireland; Department of Epidemiology and Population Health, British Columbia Centre for Excellence in HIV/AIDS, Vancouver, British Columbia, Canada; Department of Epidemiology and Population Health, British Columbia Centre for Excellence in HIV/AIDS, Vancouver, British Columbia, Canada; Department of Epidemiology and Population Health, British Columbia Centre for Excellence in HIV/AIDS, Vancouver, British Columbia, Canada; Division of Infectious Diseases, Department of Medicine, Faculty of Medicine, University of British Columbia, Vancouver, British Columbia, Canada; Department of Epidemiology and Population Health, British Columbia Centre for Excellence in HIV/AIDS, Vancouver, British Columbia, Canada; Department of Epidemiology and Population Health, British Columbia Centre for Excellence in HIV/AIDS, Vancouver, British Columbia, Canada; Department of Epidemiology and Population Health, British Columbia Centre for Excellence in HIV/AIDS, Vancouver, British Columbia, Canada; Division of Infectious Diseases, Department of Medicine, Faculty of Medicine, University of British Columbia, Vancouver, British Columbia, Canada; Department of Pathology and Laboratory Medicine, Providence Health Care, Vancouver, British Columbia, Canada; Department of Epidemiology and Population Health, British Columbia Centre for Excellence in HIV/AIDS, Vancouver, British Columbia, Canada; Division of Infectious Diseases, Department of Medicine, Faculty of Medicine, University of British Columbia, Vancouver, British Columbia, Canada; Department of Epidemiology and Population Health, British Columbia Centre for Excellence in HIV/AIDS, Vancouver, British Columbia, Canada; Department of Epidemiology and Population Health, British Columbia Centre for Excellence in HIV/AIDS, Vancouver, British Columbia, Canada; Faculty of Health Sciences, Simon Fraser University, Burnaby, British Columbia, Canada; Department of Epidemiology and Population Health, British Columbia Centre for Excellence in HIV/AIDS, Vancouver, British Columbia, Canada; Division of Infectious Diseases, Department of Medicine, Faculty of Medicine, University of British Columbia, Vancouver, British Columbia, Canada

## Abstract

**Background:**

Herpes zoster (HZ) incidence is elevated among immunocompromised populations, including people living with HIV (PLWH). We evaluated HZ incidence, associated risk factors, recurrence, and postvaccination HZ occurrence among PLWH compared with people living without HIV (PLWoH) in British Columbia, Canada.

**Methods:**

We conducted a retrospective, population-based matched cohort study using data from the Comparative Outcomes and Service Utilization Trends study (2000–2019). PLWH aged ≥19 years initiating antiretroviral therapy were propensity-score matched to PLWoH from the general population. Incident HZ was identified from administrative health records using a 12-month washout. Age-stratified time-to-event, competing-risk, and multivariable models were used to estimate incidence, risk factors, recurrence, and vaccination-associated outcomes.

**Results:**

Among 9053 PLWH and 9053 matched PLWoH, HZ incidence rates were higher among PLWH overall. Before vaccine availability, PLWH experienced substantially higher HZ hazards than PLWoH, particularly among participants aged <50 years. After 2009, HZ risk remained elevated among younger PLWH, while hazards were similar between groups among those aged ≥50 years. HZ recurrence was approximately twice as frequent among PLWH. Among PLWH, advanced immunosuppression, including low CD4 cell counts and unsuppressed viral load, and selected comorbidities were independently associated with higher HZ risk. Postvaccination HZ incidence was low in both populations, with no meaningful difference between PLWH and PLWoH; breakthrough events were uncommon.

**Conclusions:**

PLWH remain at increased risk of HZ and recurrence, especially if younger or immunosuppressed. Vaccination was associated with a lower HZ risk supporting expanded access to recombinant zoster vaccination for immunocompromised adults irrespective of age.

## Teaser

This population-based study found higher HZ incidence and recurrence among people living with HIV-1, particularly younger and immunosuppressed individuals. Vaccination was associated with low postvaccination incidence, supporting expanded access to recombinant zoster vaccination for immunocompromised adults.

Herpes zoster (HZ), commonly known as shingles, results from reactivation of latent varicella zoster virus (VZV) infection [1, 2]. Primary VZV infection usually occurs in childhood and establishes lifelong latency in the spinal and cranial sensory ganglia nerves [3]. The estimated lifetime risk of HZ in the general population is 30% [4], with incidence increasing sharply after 50 years of age [2]. Clinically, HZ presents as a unilateral, painful, erythematous, blistering rash, usually restricted to a single dermatome [5]. Complications include postherpetic neuralgia, bacterial infections, ocular disease, hearing impairments, encephalitis, stroke, and death, contributing to substantial morbidity and healthcare utilization [5, 6].

Advanced age and immunosuppression are the primary risk factors for HZ [7]. Immunocompromised states include conditions such as malignancy, transplantation, autoimmune disease, and HIV infection. Additional risk factors include chronic comorbidities such as diabetes, cardiovascular disease (CVD), renal disease, inflammatory bowel disease, systemic lupus erythematosus, rheumatoid arthritis (RA), and selected mental health conditions [8]. People living with HIV (PLWH) are at increased risk of HZ as a consequence of immune dysfunction [9]. HZ among younger people, particularly those <50 years, is often unexpected and may prompt investigation for underlying immunosuppression, including undiagnosed HIV infection [10].

The best prevention for HZ is vaccination. Two HZ vaccines have been authorized for use in Canada: live zoster vaccine (LZV) and recombinant zoster vaccine (RZV) [2]. The LZV is a single-dose live vaccine and is contraindicated in immunocompromised individuals; it was available in Canada from 2008 to 2023. In 2017, Health Canada approved the RZV, which is more effective than the LZV [11]. The RZV is a recombinant subunit vaccine administered as a two-dose schedule, 2–6 months apart; it has >90% efficacy and >80% effectiveness in adults ≥50 years [12–14]. The RZV is recommended for adults ≥50 years and for adults ≥18 years who have a weakened immune system [15]; those who received the LZV vaccine are eligible to receive this vaccine. As of 2020, an estimated 36.3% of Canadians aged 65 and older had received the HZ vaccine [16]. Vaccine coverage and accessibility differ across Canada and globally [17]. In the province of British Columbia (BC), Canada, the RZV is currently available at no cost to First Nations individuals who are 60 years and older [18].

HZ imposes a substantial individual and societal burden [6, 17]. Although previous studies have shown that HZ incidence and recurrence are higher among PLWH—including among younger individuals and those with immune dysfunction—most evidence derives from clinical cohorts or relatively small samples. Consequently, population-level data describing long-term incidence, recurrence, and the real-world impact of HZ vaccination among PLWH remain limited. These gaps are particularly important in the contemporary antiretroviral therapy (ART) era, as PLWH may continue to experience elevated HZ risk despite effective treatment.

To address these gaps, our aims were to (1) compare the incidence of HZ between PLWH and people living without HIV (PLWoH); (2) identify risk factors associated with HZ incidence among PLWH; and (3) describe HZ vaccination uptake and postvaccination HZ occurrence among participants ≥50 years, and to examine the association between vaccination status and HZ incidence.

## METHODS

### Data Source

Data were obtained from the Comparative Outcomes and Service Utilization Trends (COAST) study [19], which includes longitudinal deidentified individual-level data on all diagnosed adult PLWH in the province, and a 10% random representative sample of BC's general population, followed from 1 April 1992 to 31 March 2020. The COAST cohort is described in the Supplementary Text 1.

### Study Design and Population

This was a population-based retrospective matched cohort study. Eligible PLWH were ≥19 years old at the start of follow-up, initiated ART, and were followed for at least 1 year between 1 January 2000 and 31 December 2019. For PLWH, the index date was the latest among the following dates: first known positive HIV serostatus, 19th birthday, 5 years since the first administrative healthcare encounter, or 1 January 2000. Participants were followed until the date of death, loss-to-follow-up, or 31 December 2019, whichever came first. Given substantial baseline differences between PLWH and PLWoH, propensity-score matching (PSM) was performed prior to all analyses to improve comparability between these populations (Supplementary Text 2 and Supplementary Tables 2 and 3).

### Study Outcome

**Figure 1.**
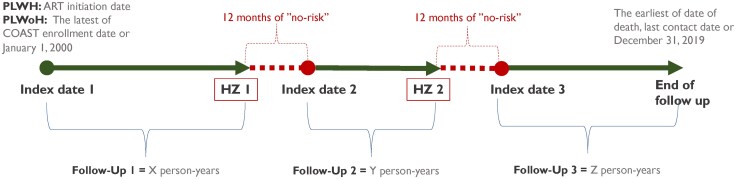
Definition of distinct HZ episodes in our database. HZ = herpes zoster; PLWH = people living with HIV; PLWoH = people living without HIV; COAST = Comparative Outcomes and Service Utilization Trends study.

The primary outcome was HZ incidence between 2000 and 2019. Incident cases were identified using the International Classification of Disease, Ninth and Tenth Revisions (ICD-9: 0.53, ICD-10: B02), diagnostic codes recorded in the physician billing and hospitalization databases, with a 12-month washout period [20]. HZ episodes occurring at least 12 months apart were considered distinct cases (Figure 1).

### Covariates

Covariates were defined as fixed (measured at index date) and time-varying (updated annually), including:

1. Sociodemographic variables (both populations): sex, age (continuous; time-varying), and history of residence in the Downtown Eastside (DTES; time-varying; yes, no). The DTES is a neighborhood within the city of Vancouver characterized by social and economic inequities (V6A postal code); it is one of the HIV epidemic epicenters among people with a history of injection drug use [21].
2. HZ-related variables (both populations): previous HZ diagnosis (Figure 1; time-varying), and HZ vaccination status (time-varying; fully vaccinated yes, no). Vaccination was ascertained from PharmaNet using Drug Identification Numbers for LZV (02315939 and 02375516), and RZV (02468425).
3. HIV-related variables: ART era (cohort effect; <2000, 2000–2009, and 2010–2019), lowest CD4 cell count (time-varying; <50, 50–199, 200–349, ≥350 cell/mm^3^, not measured), uncontrolled viremia defined by at least one viral load (VL) ≥200 copies/mL in a given year (time-varying, <100%, 100%, VL not measured) [22].
4. Comorbidities (both populations): autoimmune conditions (systemic lupus erythematosus, RA, and inflammatory bowel disease [IBD]), mood and/or anxiety disorders, schizophrenia, substance use disorder (SUD), traumatic brain injury (TBI), chronic obstructive pulmonary disease (COPD), transplant, diabetes mellitus, hypertension (HTN), asthma, chronic kidney disease (CKD), non–AIDS-defining cancer (NADC; cancer other than Kaposi's sarcoma, non–Hodgkin lymphoma, and invasive cervical [23, 24]), chronic liver disease (CLD), and CVD. Comorbidities were time-varying and identified using validated case-finding algorithms. They were assumed to continue after first identification (Supplementary Table 1). A 5-year lookback window prior to the index date was used to identify prevalent comorbidities at index date [25]. Note that, in BC, mood and anxiety disorders may also be coded under one BC-specific diagnostic code (50B) [26]. Thus, we combined these two disorders and refer to them in this article as mood/anxiety disorders (MAD).

### Statistical Analysis

Descriptive analyses used the Fisher's exact test and Kruskal-Wallis tests to compare categorical and continuous covariates, respectively [27]. Statistical analyses were conducted using SAS version 9.4.

For Aim 1, age- and sex-standardized annual HZ incidence rates were calculated using the 2011 BC Census population as the reference [28]. Incidence rates were expressed per 1000 person-years, with 95% confidence intervals (CI) estimated using the mid-p exact test [29, 30]. Because death could occur before HZ, time-to-event analyses were conducted within a competing-risk framework [31, 32]. Cumulative incidence functions for HZ and death were estimated using the Aalen-Johansen estimator [33]. Cause-specific Cox models were used to estimate unadjusted hazard ratios (uHR) for time to the first HZ.

For Aim 2, analyses were restricted to PLWH. Adjusted hazard ratios (aHR) for time to first HZ were estimated using cause-specific Cox models, with death treated as a competing event. Prior HZ was included as a covariate [34], and the proportional hazards assumption was assessed using Schoenfeld residuals. We also modeled the probability of HZ recurrence using generalized estimating equations model, assuming a binomial distribution and a logit link [35]. Vaccination status was included as a time-updated covariate. To account for informative censoring due to death and loss to follow-up, inverse probability of censoring weights were applied [36]. These weights were estimated based on baseline and time-updated covariates associated with censoring. Covariates were selected using an elimination process based on the Akaike Information Criterion and Type III *P-*values [37].

For Aim 3, HZ vaccination uptake and postvaccination HZ occurrence were described among participants aged ≥50 years. Vaccination status was treated as time-varying to reflect completion of the recommended dosing of the specific vaccine (LZV or RZV). Breakthrough HZ was defined as HZ occurring after vaccine effectiveness onset. Because the number of postvaccination HZ events was small, particularly after receipt of the RZV, breakthrough analyses were descriptive, and results should be interpreted cautiously.

Missing data occurred only for CD4 cell count and VL, reflecting intervals during which these laboratory tests were not performed. These values were coded as “not measured,” consistent with COAST methodology, and no imputation was applied. All other variables were completely observed.

## RESULTS

After PSM, the final analytical sample included 9053 PLWH and 9053 PLWoH (Supplementary Figure 1). Among PLWH, 85% were male, compared with 88% among PLWoH. Median age at index date was 39 years (25th–75th percentile [Q1–Q3]: 33–47) for PLWH and 40 years (Q1–Q3: 34–47) for PLWoH. Total follow-up time was 106 356 person-years among PLWH and 118 610 person-years among PLWoH, corresponding to a median follow-up time of 12 years (Q1–Q3: 6–18) and 14 years (Q1–Q3: 7–20), respectively.

Characteristics at index date were well balanced after PSM (Supplementary Table 4). Despite matching, small residual differences remained: PLWH were more likely to have SUD and less likely to have HTN at index date, while the prevalence of other comorbidities was similar between groups.

### Clinical Characteristics at the End of Follow-up

By the end of follow-up (Supplementary Table 4), median age had increased to 52 years (Q1–Q3: 44–59) among PLWH and 54 years (Q1–Q3: 46–61) among PLWoH. Group differences widened over time. PLWH were more likely to have resided in the DTES, and had higher cumulative prevalence of several comorbidities, including COPD, asthma, CKD, CLD, SUD, MAD, schizophrenia, and TBI (all *P* < .05). PLWH were also more likely to accumulate multiple comorbidities and were more than twice as likely as PLWoH to have experienced at least one HZ episode by study end.

### Cumulative Incidence of First HZ Before and After Vaccine Availability

**Figure 2.**
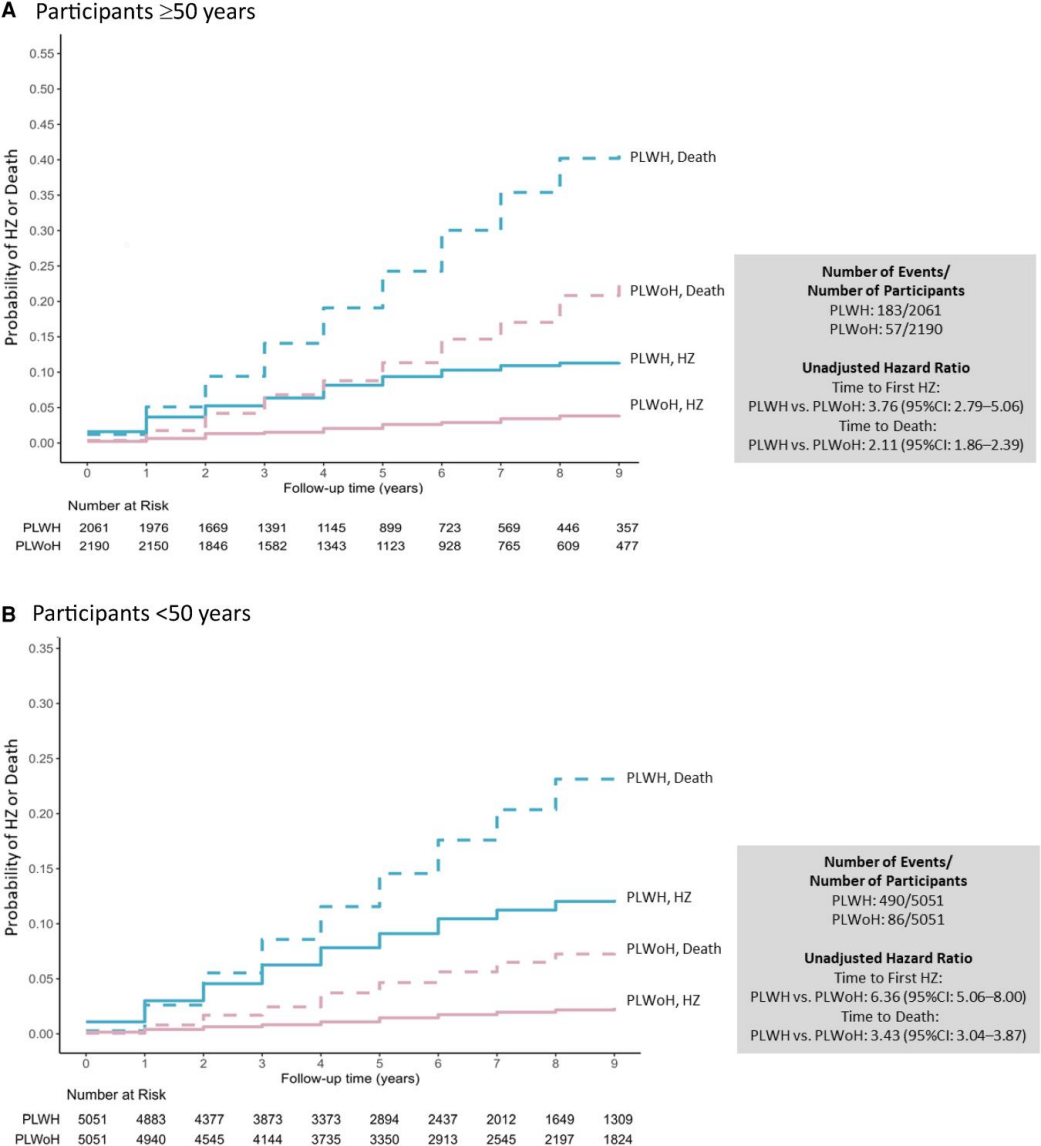
Time to first HZ infection, prior to 2009, stratified by age and HIV status, accounting for the competing risk of death. The unadjusted hazard ratio was calculated using a cause-specific hazard model. PLWoH = people living without HIV; PLWH = people living with HIV; HZ = herpes zoster; CI = confidence interval.

Before 2009, PLWH had a substantially higher cumulative incidence of first HZ than PLWoH across all age groups (Figure 2*A*, *B*). Among participants ≥50 years, the uHR for first HZ was nearly four times higher in PLWH than in PLWoH (3.76, 95% CI 2.79–5.06). Among those aged <50 years, the uHR was even greater (6.36, 95% CI 5.06–8.00), indicating a pronounced excess risk of HZ among younger PLWH.

**Figure 3.**
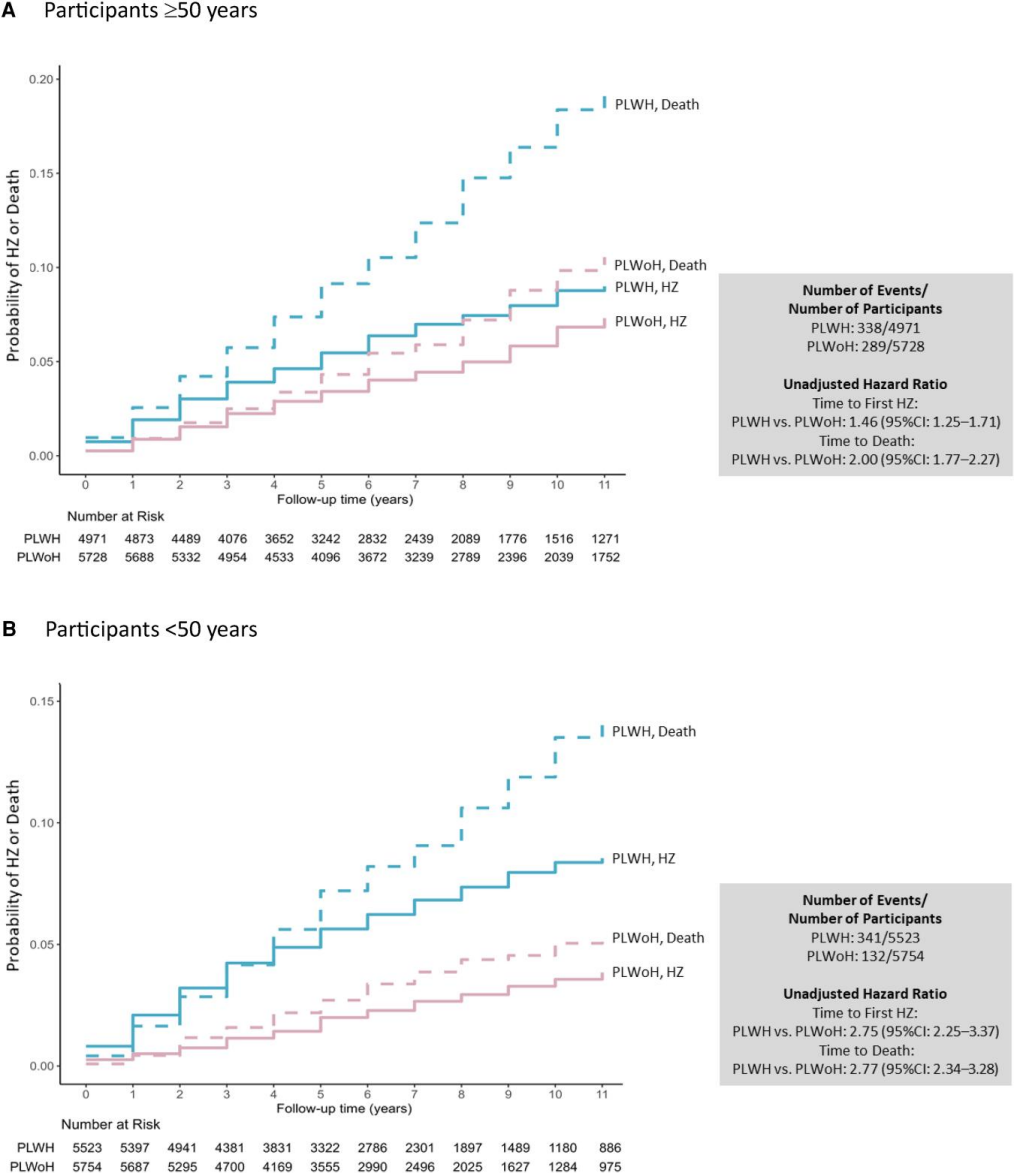
Time to first HZ infection, from 2009 onward, stratified by age and HIV status, accounting for the competing risk of death. The unadjusted hazard ratio was calculated using a cause-specific hazard model. PLWoH = people living without HIV; PLWH = people living with HIV; HZ = herpes zoster; CI = confidence interval.

After 2009, following the introduction and uptake of HZ vaccination, differences between groups narrowed (Figures 3*A*–b). Among participants aged ≥50 years, PLWH had a modestly higher hazard of HZ compared with PLWoH (uHR 1.46, 95% CI 1.25–1.71). Among those aged <50 years, the hazard remained higher among PLWH (uHR 2.75, 95% CI 2.25–3.37).

Overall, 1255 PLWH and 545 PLWoH experienced at least one HZ episode during follow-up, with incidence rates of 14.49 (95% CI: 13.18–15.80) and 6.91 (95% CI: 5.76–8.06) per 1000 person-years, respectively. Supplementary Tables 5–10 present bivariable analyses examining associations between HZ and several risk factors.

### Multivariable Competing Risk Analysis: Time to First HZ

**Figure 4.**
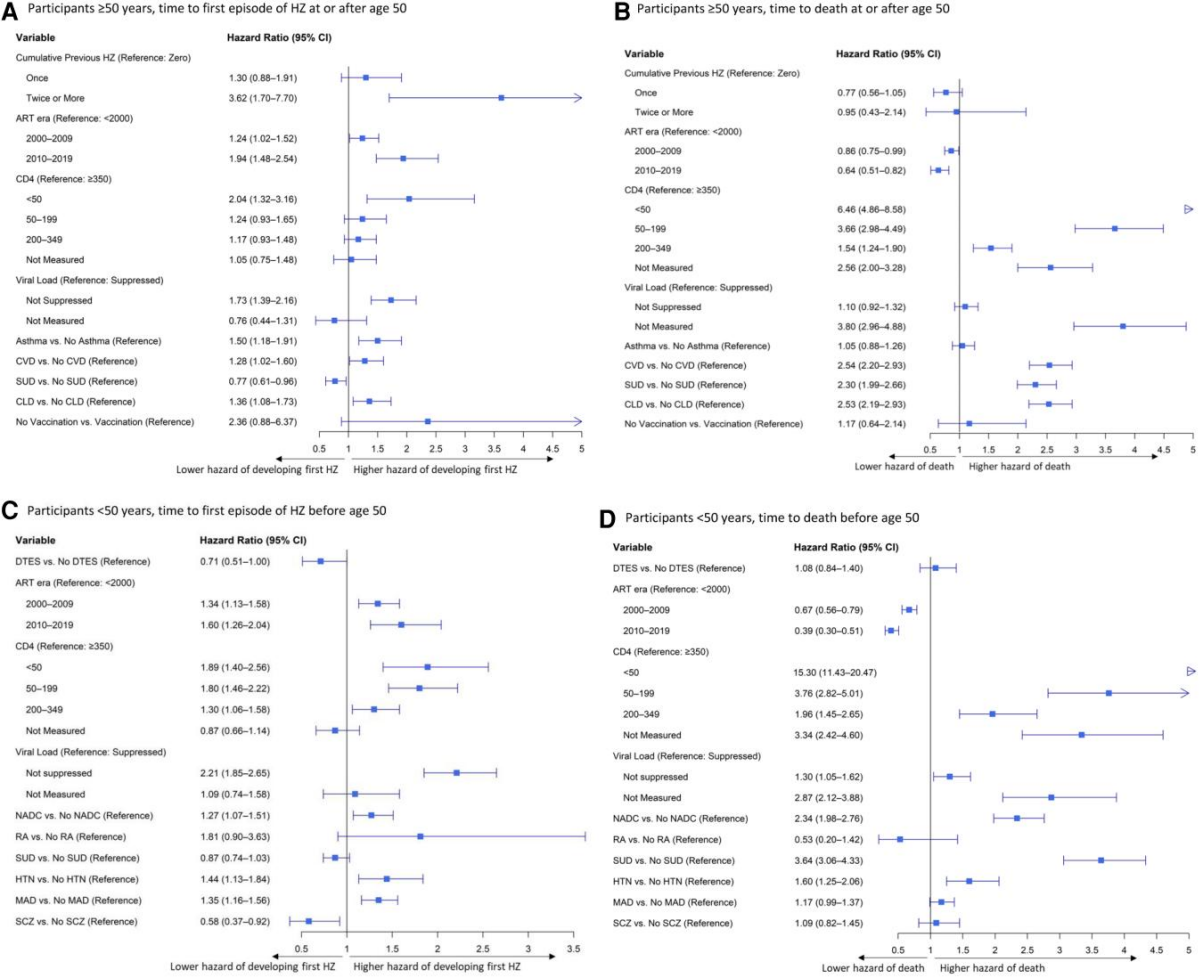
Risk factors associated with the hazard of first HZ episode and death among people living with HIV, stratified by age, estimated using cause-specific hazard models accounting for death as a competing risk. DTES = downtown eastside; ART = antiretroviral therapy; NADC = non–AIDS-defining cancer; RA = rheumatoid arthritis; SUD = substance use disorder; HTN = hypertension; MAD = mood/anxiety disorders; SCZ = schizophrenia; CLD = chronic liver disease; CVD = cardiovascular disease; HZ = herpes zoster; Tx = transplant; CI = confidence interval.

aHR for time to first HZ among PLWH are shown in Figures 4*A*–*D*. Among PLWH aged ≥50 years, prior HZ was the strongest predictor of subsequent HZ, particularly among those with two or more previous episodes (aHR 3.62, 95% CI 1.70–7.70). Advanced HIV disease was also strongly associated with HZ, including CD4 < 50 cells/mm³ (aHR 2.04, 95% CI 1.32–3.16) and unsuppressed VL (aHR 1.73, 95% CI 1.39–2.16). More recent ART eras, particularly 2010–2019, were associated with higher HZ risk compared with the pre-2000 era (aHR 1.94, 95% CI 1.48–2.54). Among comorbidities, asthma (aHR 1.50, 95% CI 1.18–1.91) and CLD (aHR 1.36, 95% CI 1.08–1.73) showed the strongest associations. HZ vaccination was associated with a lower hazard of HZ (aHR 0.42, 95% CI .16–1.14), although estimates were not statistically significant likely due to few postvaccination events. SUD was associated with a lower observed hazard of HZ (aHR 0.77, 95% CI .61–.96).

Among PLWH aged <50 years, HIV disease severity remained the primary determinant of HZ risk. Unsuppressed VL (aHR 2.21, 95% CI 1.85–2.65) and lower CD4 (CD4 < 50: aHR 1.89, 95% CI 1.40–2.56; CD4 50–199: aHR 1.80, 95% CI 1.46–2.22) were strongly associated with HZ. More recent ART eras were again associated with increased HZ risk. Among comorbidities, HTN (aHR 1.44, 95% CI 1.13–1.84) and MAD (aHR 1.35, 95% CI 1.16–1.56) showed the strongest positive associations, while DTES residence (aHR 0.71, 95% CI .51–1.00) and schizophrenia (aHR 0.58, 95% CI .37–.92) were associated with lower observed hazards.

### Multivariable Analysis of HZ Recurrence

**Figure 5.**
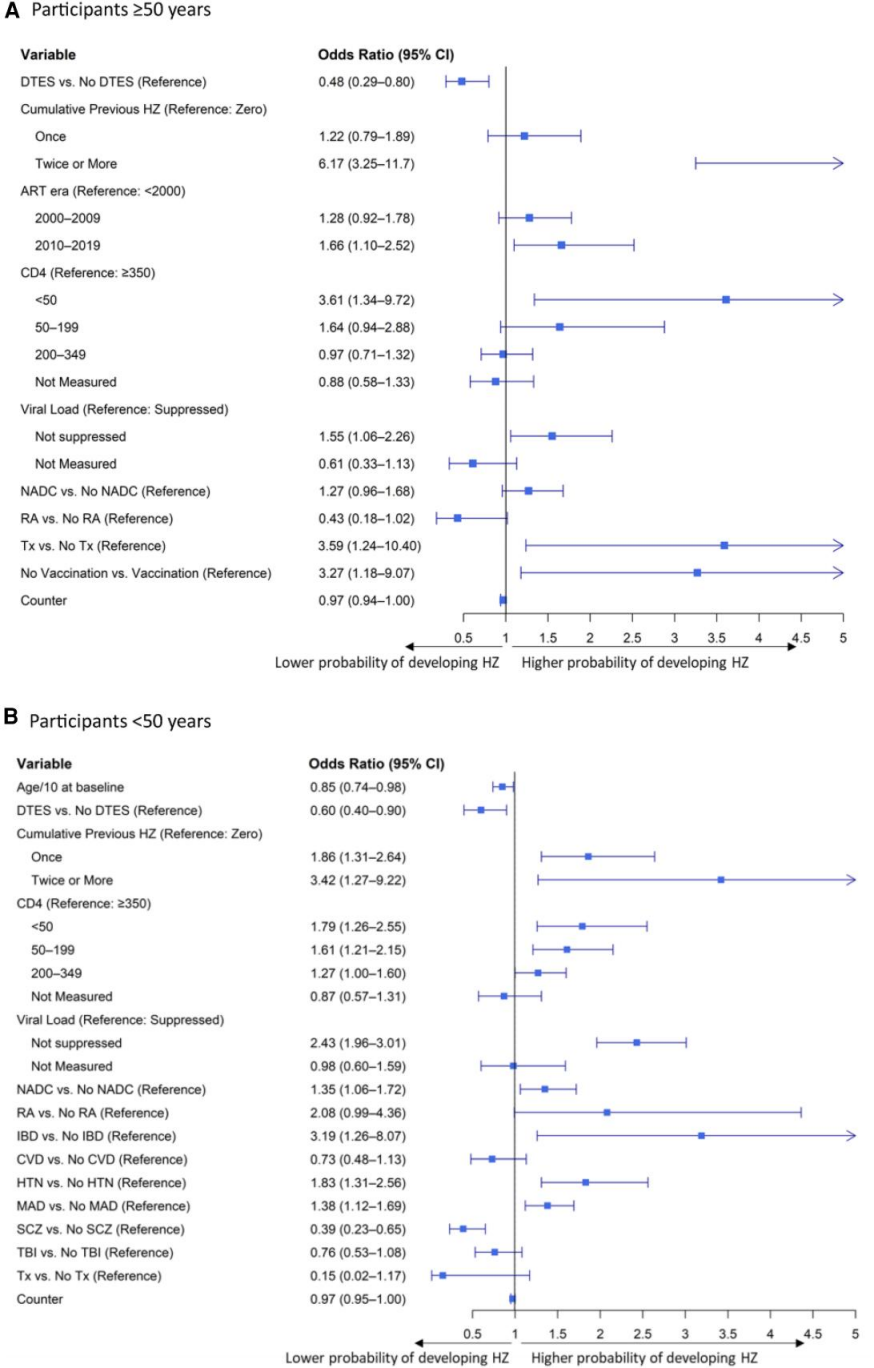
Risk factors associated with the probability of developing HZ among people living with HIV, estimated using inverse probability weighted generalized estimating equations model, stratified by age. HZ = herpes zoster; DTES = downtown eastside; ART = antiretroviral therapy; NADC = non–AIDS-defining cancer; RA = rheumatoid arthritis; IBD = inflammatory bowel disease; CVD = cardiovascular disease; HTN = hypertension; MAD = mood/anxiety disorders; SCZ = schizophrenia; TBI = traumatic brain injury; Tx = transplant; CI = confidence interval.

Adjusted odds ratios for experiencing recurrent HZ among PLWH are shown in Figure 5*A*, *B*. Among PLWH aged ≥50 years, prior HZ was the strongest predictor of HZ recurrence, particularly among those with two or more prior episodes (aOR 6.17, 95% CI 3.25–11.7). Advanced HIV disease, including CD4 < 50 cells/mm³ (aOR 3.61, 95% CI 1.34–9.72) and unsuppressed VL (aOR 1.55, 95% CI 1.06–2.26), transplant history (aOR 3.59, 95% CI 1.24–10.40), and lack of vaccination (aOR 3.27, 95% CI 1.18–9.07) were associated with higher odds of HZ. DTES residence was associated with lower odds of HZ (aOR 0.48, 95% CI .29–.80).

Among PLWH aged <50 years, prior HZ remained a strong predictor, with increasing odds for one prior episode (aOR 1.86, 95% CI 1.31–2.64) and two or more episodes (aOR 3.42, 95% CI 1.27–9.22). The same other core risk factors were also associated with an increased odds of HZ. The principal differences in this younger group were the prominence of IBD (aOR 3.19, 95% CI 1.26–8.07) and mood/anxiety disorders (aOR 1.38, 95% CI 1.12–1.69) as associated comorbidities, whereas transplant history and vaccination status did not emerge as independent predictors. DTES residence (aOR 0.60, 95% CI .40–.90) and schizophrenia (aOR 0.39, 95% CI .23–.65) were also associated with lower odds of observed HZ.

### Postvaccination HZ Incidence

Standardized HZ incidence rates following vaccination were low overall and accompanied by wide CIs, reflecting the small number of postvaccination events (Supplementary Tables 11 and 12). Among participants aged ≥50 years who received LZV, HZ incidence was 3.79 per 1000 person-years (95% CI .73–6.85) in PLWH and 5.86 per 1000 person-years (95% CI 1.04–10.67) in PLWoH. Following RZV receipt, HZ events were extremely uncommon in both groups, precluding stable incidence estimation.

### Subanalyses

Supplementary Tables 13–18 present post-PSM bivariable comparisons stratified by DTES residence, schizophrenia, and SUD to understand the reason why their aHR and aOR were below one in our analyses. Across strata, HZ events were infrequent and concentrated within small subgroups. PLWH who developed HZ were generally older and more likely to have accumulated more chronic conditions such as NADC, HTN, CKD, or CVD. In contrast, those who did not develop HZ more often were younger and showed characteristics associated with structural vulnerability, including DTES residence, and markers of uncontrolled HIV disease. Vaccination uptake was uncommon in these strata.

## DISCUSSION

In this population-based study, we compared the HZ incidence between PLWH and PLWoH, identified risk factors associated with experiencing at least one HZ episode, and examined the association between zoster vaccination and subsequent HZ. Before 2009, the HZ hazard among PLWH was six and four times higher than PLWoH for those aged <50 vs ≥50 years, respectively. After 2009, the HZ hazard among PLWH aged <50 remained approximately three times higher than PLWoH, whereas, among those aged ≥50 years, the hazards were similar in both groups. HZ recurrence was twice as frequent among PLWH. Low CD4, unsuppressed VL, and several comorbidities were associated with a higher likelihood of developing HZ at least once, regardless of age. These findings are consistent with the role of immune dysfunction and chronic inflammatory conditions in promoting VZV reactivation. Note that the association between later ART eras and higher observed HZ incidence likely reflects calendar-time effects (eg, cohort aging and improved clinical detection) rather than a causal increase in HZ risk.

DTES residence, schizophrenia, and SUD were associated with lower hazards and odds of HZ in multivariable models. These inverse associations should not be interpreted as protective effects. Rather, subanalyses demonstrated that these groups of participants experienced substantially higher mortality and shorter follow-up time, resulting in a reduced opportunity for HZ to be observed. This pattern is consistent with the excess mortality associated with structural vulnerability and the ongoing opioid toxicity crisis in BC, and underscores the importance of accounting for competing risks when evaluating age-related outcomes in these populations.

Among vaccinated participants, postvaccination HZ incidence was low and no longer meaningfully differed by HIV status. Breakthrough events were uncommon and occurred less frequently among PLWH. Several factors may explain this pattern. First, vaccine type differed between groups, with a larger proportion of vaccinated PLWH receiving the RZV, which has demonstrated higher protection against HZ than the LZV. In addition, the RZV was introduced late in the observation window, limiting postvaccination person-time. Second, among PLWH, vaccination was more common among participants with markers of better health status and engagement in HIV care, including higher CD4 cell counts and viral suppression, suggesting selection and residual confounding may have contributed to lower observed breakthrough rates in this group. Finally, event counts were small and CIs were wide; therefore, these findings should be interpreted with caution.

Our study also demonstrated that low CD4 and comorbidities—such as asthma, CVD, NADC, HTN, CLD, and MAD—further increased the risk of PLWH developing HZ. Prior studies in the general population support these findings and highlight that having HIV poses a significant risk of HZ. Steinmmann et al [38] found the highest risk of HZ among participants with transplantation, lupus, NADC, hematological disorders, and HIV. Marra et al [20] showed that HIV/AIDS, malignancies, and systemic lupus erythematosus had relative risks greater than two. That study also identified that family history and physical trauma as important risk factors. Marra et al [39] showed elevated HZ risk among patients treated with immunosuppressants for autoimmune conditions (eg, RA, systemic lupus erythematosus, IBD). Also, in Marra et al [39], the authors showed that patients treated with corticosteroids (eg, with asthma) were at an elevated risk of HZ, as was likewise demonstrated in our study. Although autoimmune conditions treated with long-term systemic immunosuppressive therapy have been associated with a higher risk of HZ in previous studies, the small number of these conditions in our cohort limited our ability to detect such effects. By contrast, asthma appeared significant, primarily because of its higher prevalence rather than because its usual treatments—mostly inhaled corticosteroids or short systemic courses—confer a greater degree of systemic immunosuppression.

### Public Health and Policy Implications

Our findings support reconsideration of eligibility criteria for publicly funded or private insurance–covered RZV, irrespective of age, among immunocompromised populations. In Canada and the United States, each dose of the RZV costs CAD (Canadian dollars) 160 and USD 198 (U.S. dollars) out-of-pocket [40, 41], and in Canada, public coverage varies by jurisdiction [6]. In most settings, the RZV is available to immunocompromised individuals ≥18 years, or it is based on the minimum age, usually ranging between 50 and 65 years. In BC, the provincial government and most private health insurance plans do not cover the cost of this vaccine, which precludes eligible people, especially those on a fixed income, from taking this highly effective vaccination. Evidence from the United States shows that vaccine uptake increased substantially after cost barriers were removed [42], and multiple studies from Canada, the United States, and other parts of the world have demonstrated that the RZV is safe, cost-effective, and cost-saving [43, 44].

### Strengths and Limitations

This study has several strengths, including 19 years of population-based administrative and clinical data, a large sample size permitting age-stratified analyses, and a matched cohort design that reduced confounding when comparing PLWH with the general population. Use of the COAST cohort allowed linkage of comprehensive HIV clinical data with provincial healthcare records, and comorbidities were identified using validated BC Ministry of Health case definitions.

Limitations include reliance on administrative data, which may be subject to coding errors and incomplete capture of HZ cases that did not result in healthcare encounters. We were unable to account for certain risk factors, such as ethnicity, trauma, or family history. Vaccine uptake may be underestimated, particularly for vaccinations received outside BC or early after RZV introduction in 2018. In addition, the LZV was historically contraindicated for PLWH with low CD4 counts, which may have influenced uptake patterns. Finally, we were unable to assess complications of HZ, like postherpetic neuralgia, due to power issues as we only observed seven cases of this complication.

## CONCLUSIONS

It is predicted that the BC population aged ≥50 years will more than double by 2050, and it will be important to address their evolving healthcare needs. Since age is a key risk factor for the reactivation of the VZV due to immunosenescence, it is expected that the incidence of HZ will increase [45, 46]. In conclusion, broadening eligibility for RZV among immunocompromised adults aged ≥18 years and among all adults aged ≥50 years could yield substantial public health benefits by preventing HZ-related morbidity, healthcare utilization, and long-term complications.

## ETHICS

The University of British Columbia/Providence Health Care Research Ethics Board and Simon Fraser University Office of Research Ethics provided ethics approval for this study (H09-02905; H22-02875). The study complied with the BC Freedom of Information and Protection of Privacy Act and did not require informed consent as it is conducted retrospectively for research and statistical purposes only using anonymized data.

## Supplementary Material

jiag118_Supplementary_Data

## Supplementary Data

Supplementary materials are available at *The Journal of Infectious Diseases* online (http://jid.oxfordjournals.org/). Supplementary materials consist of data provided by the author that are published to benefit the reader. The posted materials are not copyedited. The contents of all supplementary data are the sole responsibility of the authors. Questions or messages regarding errors should be addressed to the author.

## References

[1] Centers for Disease Control and Prevention. Shingles (Herpes zoster). https://www.cdc.gov/shingles/index.html. Accessed 19 June 2024.

[2] Government of Canada. Herpes zoster (Shingles) vaccine: Canadian immunization guide for health professionals. https://www.canada.ca/en/public-health/services/publications/healthy-living/canadian-immunization-guide-part-4-active-vaccines/page-8-herpes-zoster-(shingles)-vaccine.html#a5. Accessed 17 June 2024.

[3] Sampathkumar P, Drage LA, Martin DP. Herpes zoster (shingles) and postherpetic neuralgia. Mayo Clin Proc 2009; 84:274–80. 19252116 10.4065/84.3.274 PMC2664599

[4] Yawn BP, Saddier P, Wollan PC, et al A population-based study of the incidence and complication rates of herpes zoster before zoster vaccine introduction. Mayo Clin Proc 2007; 82:1341–9. 17976353 10.4065/82.11.1341

[5] Patil A, Goldust M, Wollina U. Herpes zoster: a review of clinical manifestations and management. Viruses 2022; 14:192. 35215786 10.3390/v14020192 PMC8876683

[6] National Institute on Ageing: Ryerson University. The overlooked issue of shingles infections in older Canadians and how to address it! https://static1.squarespace.com/static/5c2fa7b03917eed9b5a436d8/t/66194eeb7f54fd7779e3d27b/1712934636474/Shingles+Report+-+Final3.pdf. Accessed 10 July 2024.

[7] McKay SL, Guo A, Pergam SA, Dooling K. Herpes zoster risk in immunocompromised adults in the United States: a systematic review. Clin Infect Dis 2020; 71:e125–34. 31677266 10.1093/cid/ciz1090 PMC7195255

[8] Batram M, Witte J, Schwarz M, et al Burden of herpes zoster in adult patients with underlying conditions: analysis of German claims data, 2007-2018. Dermatol Ther (Heidelb) 2021; 11:1009–26. 33959878 10.1007/s13555-021-00535-7 PMC8163947

[9] Dauby N, Motet C, Libois A, Martin C. The value of herpes zoster prevention in people aging with HIV: a narrative review. HIV Med 2023; 24:1190–7. 37772682 10.1111/hiv.13548

[10] Nanditha NGA, St-Jean M, Tafessu H, et al Missed opportunities for earlier diagnosis of HIV in British Columbia, Canada: a retrospective cohort study. PLoS One 2019; 14:e0214012. 30897143 10.1371/journal.pone.0214012 PMC6428302

[11] Public Health Agency of Canada. An Advisory Committee Statement (ACS). National Advisory Committee on Immunization (NACI): updated recommendations on the use of herpes zoster vaccines. https://www.canada.ca/en/services/health/publications/healthy-living/updated-recommendations-use-herpes-zoster-vaccines.html. Accessed 17 June 2024.

[12] Lal H, Cunningham AL, Godeaux O, et al Efficacy of an adjuvanted herpes zoster subunit vaccine in older adults. N Engl J Med 2015; 372:2087–96. 25916341 10.1056/NEJMoa1501184

[13] Sun Y, Kim E, Kong CL, Arnold BF, Porco TC, Acharya NR. Effectiveness of the recombinant zoster vaccine in adults aged 50 and older in the United States: a claims-based cohort study. Clin Infect Dis 2021; 73:949–56. 33580245 10.1093/cid/ciab121 PMC8442779

[14] Dooling KL, Guo A, Patel M, et al Recommendations of the advisory committee on immunization practices for use of herpes zoster vaccines. MMWR Morb Mortal Wkly Rep 2018; 67:103–8. 29370152 10.15585/mmwr.mm6703a5 PMC5812314

[15] Immunize BC. Shingles vaccine. https://immunizebc.ca/vaccines/shingles. Accessed 18 July 2024.

[16] Gilmour H . Factors associated with shingles and pneumococcal vaccination among older Canadians. https://www150.statcan.gc.ca/n1/pub/82-003-x/2024001/article/00002-eng.htm. Accessed 19 June 2024. 10.25318/82-003-x202400100002-eng 38232409

[17] Pan CX, Lee MS, Nambudiri VE. Global herpes zoster incidence, burden of disease, and vaccine availability: a narrative review. Ther Adv Vaccines Immunother 2022; 10:25151355221084535. 35340552 10.1177/25151355221084535 PMC8941701

[18] British Columbia's First Nations Health Authority. Recent changes to coverage of Shingrix® Vaccine and FreeStyle® Libre 2 glucose monitor. https://www.fnha.ca/benefits/health-benefits-news/recent-changes-to-coverage-of-shingrix-vaccine-and-freestyle-libre-2-glucose-monitor. Accessed 19 June 2024.

[19] Budu MO, Kooij KW, Heath K, et al Cohort profile update: reflecting back and looking ahead: updating the Comparative Outcomes and Service Utilization Trends (COAST) study to include 28 years of linked data from people with and without HIV in British Columbia, Canada. Int J Popul Data Sci 2025; 10:2496. 40110112 10.23889/ijpds.v10i1.2496 PMC11922098

[20] Marra F, Chong M, Najafzadeh M. Increasing incidence associated with herpes zoster infection in British Columbia, Canada. BMC Infect Dis 2016; 16:589. 27765026 10.1186/s12879-016-1898-z PMC5073843

[21] Linden IA, Mar MY, Werker GR, Jang K, Krausz M. Research on a vulnerable neighborhood-the Vancouver downtown eastside from 2001 to 2011. J Urban Health 2013; 90:559–73. 23161093 10.1007/s11524-012-9771-x PMC3665976

[22] Lima VD, St-Jean M, Rozada I, et al Progress towards the United Nations 90-90-90 and 95-95-95 targets: the experience in British Columbia, Canada. J Int AIDS Soc 2017; 20:e25011. 29130644 10.1002/jia2.25011 PMC5810311

[23] Chiu CG, Smith D, Salters KA, et al Overview of cancer incidence and mortality among people living with HIV/AIDS in British Columbia, Canada: implications for HAART use and NADM development. BMC Cancer 2017; 17:270. 28410587 10.1186/s12885-017-3229-1 PMC5391557

[24] Choi HG, Zehnder JL, Lee YK, Lim H, Kim M. Increased risk of lymphoid malignancy in patients with herpes zoster: a longitudinal follow-up study using a national cohort. BMC Cancer 2019; 19:1148. 31775678 10.1186/s12885-019-6349-y PMC6882027

[25] Nanditha NGA, Dong X, McLinden T, et al The impact of lookback windows on the prevalence and incidence of chronic diseases among people living with HIV: an exploration in administrative health data in Canada. BMC Med Res Methodol 2022; 22:1. 34991473 10.1186/s12874-021-01448-x PMC8734246

[26] British Columbia Ministry of Health. British Columbia Chronic Disease Registries (BCCDR) case definitions - mood and anxiety disorders. http://www.bccdc.ca/resource-gallery/Documents/Chronic-Disease-Dashboard/mood-anxiety-disorders.pdf. Accessed 27 October 2023.

[27] McDonald JH . Handbook of biological statistics. Baltimore, MD: Sparky House Publishing, 2009.

[28] Gordis L . Epidemiology. 5th ed. Philadelphia, PA: Elsevier Saunders, 2014.

[29] Daly LE . Confidence limits made easy: interval estimation using a substitution method. Am J Epidemiol 1998; 147:783–90. 9554420 10.1093/oxfordjournals.aje.a009523

[30] Rothman KJ, Boice JD. Epidemiologic analysis with a programmable calculator. Vol. no. 79–1649. Bethesda, MD; Washington: U.S. Dept. of Health, Education, and Welfare, Public Health Service, National Institutes of Health, 1979:25–32.

[31] Schuster NA, Hoogendijk EO, Kok AAL, Twisk JWR, Heymans MW. Ignoring competing events in the analysis of survival data may lead to biased results: a nonmathematical illustration of competing risk analysis. J Clin Epidemiol 2020; 122:42–8. 32165133 10.1016/j.jclinepi.2020.03.004

[32] Austin PC, Lee DS, Fine JP. Introduction to the analysis of survival data in the presence of competing risks. Circulation 2016; 133:601–9. 26858290 10.1161/CIRCULATIONAHA.115.017719 PMC4741409

[33] Aalen OO, Johansen S. An empirical transition matrix for non-homogeneous Markov chains based on censored observations. Scand J Stat 1978; 5:141–50.

[34] Parikh R, Spence O, Giannelos N, Kaan I. Herpes zoster recurrence: a narrative review of the literature. Dermatol Ther (Heidelb) 2024; 14:569–92. 38416279 10.1007/s13555-024-01101-7 PMC10965844

[35] McCullagh P, Nelder JA. Generalized linear models: monographs on statistics and applied probability 37. 2nd ed. Boca Raton, FL: Chapman and Hall/CRC, 1989.

[36] Robins JM, Finkelstein DM. Correcting for noncompliance and dependent censoring in an AIDS clinical trial with inverse probability of censoring weighted (IPCW) log-rank tests. Biometrics 2000; 56:779–88. 10985216 10.1111/j.0006-341x.2000.00779.x

[37] Lima VD, Bangsberg DR, Harrigan PR, et al Risk of viral failure declines with duration of suppression on highly active antiretroviral therapy irrespective of adherence level. J Acquir Immune Defic Syndr 2010; 55:460–5. 20838225 10.1097/QAI.0b013e3181f2ac87 PMC2974791

[38] Steinmann M, Lampe D, Grosser J, et al Risk factors for herpes zoster infections: a systematic review and meta-analysis unveiling common trends and heterogeneity patterns. Infection 2024; 52:1009–26. 38236326 10.1007/s15010-023-02156-y PMC11142967

[39] Marra F, Lo E, Kalashnikov V, Richardson K. Risk of herpes zoster in individuals on biologics, disease-modifying antirheumatic drugs, and/or corticosteroids for autoimmune diseases: a systematic review and meta-analysis. Open Forum Infect Dis 2016; 3:ofw205. 27942537 10.1093/ofid/ofw205 PMC5144657

[40] Ministry of Health. ImmunizeBC: where can I get the Shingles vaccine? How much does it cost? https://immunizebc.ca/ask-us/questions/where-can-i-get-shingrixⓡ-vaccine. Accessed 10 July 2024.

[41] US Centers for Disease Control and Prevention . Current CDC vaccine price list. https://www.cdc.gov/vaccines-for-children/php/price-list/?CDC_AAref_Val=https://www.cdc.gov/vaccines/programs/vfc/awardees/vaccine-management/price-list/index.html. Accessed 10 July 2024.

[42] Qato DM, Romley JA, Myerson R, Goldman D, Fendrick AM. Shingles vaccination in medicare part D after inflation reduction act elimination of cost sharing. JAMA 2024; 331:2043–5. 38780935 10.1001/jama.2024.7348 PMC11117145

[43] George S, Carrico J, Hicks KA, et al Updated public health impact and cost effectiveness of recombinant zoster vaccine in Canadian adults aged 50 years and older. Pharmacoecon Open 2024; 8:481–92. 38605257 10.1007/s41669-024-00483-w PMC11058134

[44] Giannelos N, Ng C, Curran D. Cost-effectiveness of the recombinant zoster vaccine (RZV) against herpes zoster: an updated critical review. Hum Vaccin Immunother 2023; 19:2168952. 36916240 10.1080/21645515.2023.2168952 PMC10054181

[45] United Nations. World Social Report 2023: leaving no one behind in an ageing world. https://www.un.org/development/desa/dspd/wp-content/uploads/sites/22/2023/01/WSR_2023_Chapter_Key_Messages.pdf. Accessed 19 July 2924.

[46] World Health Organization. Ageing and health. https://www.who.int/news-room/fact-sheets/detail/ageing-and-health. Accessed 19 July 2024.

[47] BC Centre for Excellence in HIV/AIDS. Drug treatment programs. https://bccfe.ca/drug-treatment-programs/. Accessed 13 November 2024.

[48] British Columbia Ministry of Health. Vital statistics deaths [dataset]. Victoria, BC: Population Data BC, **2021**.

