# Supplementary material for: Incidence of Herpes Zoster Infection (Shingles) Among Adults Living With and Without HIV in British Columbia, Canada: A Population-based Study
Source: J Infect Dis. 2026 Mar 10;234(1):e69–80. doi: 10.1093/infdis/jiag118 (PMC13431671; doi:10.1093/infdis/jiag118)
Supplement: jiag118_Supplementary_Data [file jiag118_supplementary_data.docx]

**Incidence of Herpes Zoster Infection (Shingles) Among Adults Living With and Without HIV in British Columbia, Canada: A Population-Based Study.**

*Corresponding author: Dr. Viviane D. Lima

British Columbia Centre for Excellence in HIV/AIDS

608-1081 Burrard Street, Vancouver, BC, Canada V6Z 1Y6

Tel: 1-604-806-8796

E-mail: [vlima@bccfe.ca](mailto:vlima@bccfe.ca)

Table of Contents

[Text S1. Description of linked databases in COAST relevant to this study. 3](#_Toc219479551)

[Table S1. List of diseases and respective case-finding algorithms. 4](#_Toc219479552)

[Figure S1. Flowchart describing eligibility criteria and derivation of the final analytical samples of people living with HIV (PLWH) and people living without HIV (PLWoH). 22](#_Toc219479553)

[Table S2. Descriptive characteristics of all eligible participants before propensity score matching, by HIV status. 23](#_Toc219479554)

[Text S2. Propensity Score Matching Overview and Diagnostic Assessment 27](#_Toc219479555)

[Figure S2. Propensity score distributions before and after matching 28](#_Toc219479556)

[Figure S3. Standardised mean differences before and after matching 29](#_Toc219479557)

[Table S3. Characteristics of individuals retained versus excluded after propensity score matching, at index date. 31](#_Toc219479558)

[Table S4. Descriptive characteristics of included participants after propensity score matching, by HIV status. 33](#_Toc219479559)

[Table S5. Bivariable comparison of study variables by herpes zoster status among people living with HIV. 37](#_Toc219479560)

[Table S6. Bivariable comparison of study variables by herpes zoster status among people living without HIV. 41](#_Toc219479561)

[Table S7. Bivariable comparison of study variables by the number of episodes of herpes zoster (HZ) infection among people living with HIV. 45](#_Toc219479562)

[Table S8. Bivariable comparison of study variables by the number of episodes of herpes zoster (HZ) infection among people living without HIV. 49](#_Toc219479563)

[Table S9. Bivariable comparison of study variables by herpes zoster (HZ) vaccination status among people living with HIV aged 50 years and over. 52](#_Toc219479564)

[Table S10. Bivariable comparison of study variables by herpes zoster (HZ) vaccination status among people living without HIV aged 50 years and over. 55](#_Toc219479565)

[Table S11. Standardized cumulative incidence rate (CIR) (per 1000 person-years) of herpes zoster (HZ) infection over the study period, by HIV status, for (A) the total population, and (B) those who received a complete course of HZ vaccination. 57](#_Toc219479566)

[Table S12. Number of people who developed herpes zoster (HZ) infection after completing vaccination for those aged ≥50 years, by HIV status. 58](#_Toc219479567)

[Table S13. Bivariable comparison of study variables among people living with HIV, under 50 years old, who never versus ever developed herpes zoster (HZ) infection after being diagnosed with schizophrenia. 59](#_Toc219479568)

[Table S14. Bivariable comparison of study variables among people living with HIV, aged 50 years and over, who never versus ever developed herpes zoster (HZ) infection after being diagnosed with schizophrenia. 63](#_Toc219479569)

[Table S15. Bivariable comparison of study variables among people living with HIV, under 50 years old, who never versus ever developed herpes zoster (HZ) infection after being diagnosed with substance use disorder (SUD). 67](#_Toc219479570)

[Table S16. Bivariable comparison of study variables among people living with HIV, aged 50 years and over, who never versus ever developed herpes zoster (HZ) infection after being diagnosed with substance use disorder (SUD). 71](#_Toc219479571)

[Table S17. Bivariable comparison of study variables among people living with HIV, under 50 years old, who never versus ever developed herpes zoster (HZ) infection after residing in Vancouver’s Downtown Eastside (DTES). 75](#_Toc219479572)

[Table S18. Bivariable comparison of study variables among people living with HIV, aged 50 years and over, who never versus ever developed herpes zoster (HZ) infection after residing in Vancouver’s Downtown Eastside (DTES). 79](#_Toc219479573)

[References 83](#_Toc219479574)

# **Text S1**. Description of linked databases in COAST relevant to this study.

| **Database** | **Description** |
| --- | --- |
| Drug Treatment Program (DTP)[1] | The DTP collects data on age, sex, gender, place of residence (including information on access to housing), mode of HIV transmission, health behaviour (e.g., substance use), laboratory test results (e.g., CD4, viral load, hepatitis C), clinical staging of HIV, duration of HIV infection, and treatment-related factors (including ART dispensation and retention). |
| COAST[2-4] | COAST (Comparative Outcomes and Service Utilization Trends) is a population-based longitudinal cohort study that links the DTP and several administrative databases through Population Data BC, the province’s repository of administrative health data. COAST currently includes individual-level non-identifiable data on ever diagnosed people living with HIV and a random 10% comparison sample of the total BC adult HIV-negative population. COAST contains millions of records containing lifetime health information on both populations since the date of the first-ever encounter with the BC healthcare system as early as 1992. The administrative databases we will use within COAST include:  (1) Medical Services Plan, BC's universal insurance program that collects data on outpatient medical services provided by fee-for-service practitioners. This database includes the billing number, name, specialty, and professional address of the prescriber;  (2) Discharge Abstracts Database, which contains data on discharges, transfers, and deaths of inpatients and day-surgery patients from acute care hospitals across BC;  (3) PharmaCare, BC's public drug insurance program that pays for eligible prescription drugs;  (4) PharmaNet, the province-wide network that links all BC pharmacies to a centralized data system. In addition to information on the drugs’ payers (individual, government, or private insurance), it also contains information regarding Opioid Substitution Therapy and COVID-19 vaccines administered by pharmacies;  (5) BC Vital Statistics, which contains death records and causes of death of all BC’s residents;  (6) National Ambulatory Care Reporting System, which includes data on all levels of hospital-based and community-based ambulatory care, including emergency departments;  (7) BC Cancer, which contains records of all cancer diagnoses (including date of diagnosis, type, and site), treatment (including medications), and outcomes reported to this registry;  (8) Providence Health Care Laboratory Interface, which includes results from routine and specialized tests, including COVID-19 test results for the DTP patients. |
| **Note.** | BC: British Columbia; ART: antiretroviral therapy |

# **Table S1.** List of diseases and respective case-finding algorithms.

| **Comorbidities** | **Case Definitions** | **Diagnostic, Procedure, Drug Code** | **References** |
| --- | --- | --- | --- |
| Diabetes Mellitus (DM) | 1 or more hospitalization with a DM diagnostic code  OR  2 or more physician visits DM diagnostic codes(s) in one year  OR  2 or more insulin prescriptions in one-year  OR  2 or more oral antihyperglycemic (not including metformin) prescriptions in one-year  OR  1 insulin and one oral antihyperglycemic (including metformin) in one year  OR  2 metformin prescriptions and one physician visit with DM diagnostic code in one year.  Note: Cases of suspected gestational diabetes in women aged 10-54 are not included by excluding hospitalizations, physician claims or prescriptions within the time period 120 days preceding or 180 days after hospital records containing birth-related diagnostic codes. | **ICD-9:**   - 250 (Diabetes mellitus)   **ICD-10**   - E10 (Type 1 diabetes mellitus) - E11 (Type 2 diabetes mellitus) - E13 (Other specified diabetes mellitus) - E14 (Unspecified diabetes mellitus)   **ICD-9 excluded:** (641, 642, 643, 644, 645, 646, 647, 648, 649, 650, 651, 652, 653, 654, 655, 656, 657, 658, 659, 660, 661, 662, 663, 664, 665, 666, 667, 668, 669, 670, 671, 672, 673, 674, 675, 676, 763.)  **ICD-10 excluded:** (V27, O10, O11, O12, O13, O14, O15, O16, O21, O22, O23, O24, O25, O26, O28, O29, O30, O31, O32, O33, O34, O35, O36, O37, O40, O41, O42, O43, O44, O45, O46, O47, O48, O60, O61, O62, O63, O64, O65, O66, O67, O68, O69).  If there’s medication taken by patients with the following diagnosis or codes for medication prescription are not enough to determine diabetes.  ICD-9/10:   - 256.4 (polycystic ovarian syndrome) - 249 (Secondary (chemical induced) diabetes) - 790.29 (Hyperglycemia) - E28.2 (Polycystic ovarian syndrome) - R73.09 (Hyperglycemia, Other abnormal glucose)   **Medication codes:**  2303442 2303450 2303469 2365286 2408228 2365294 2408236 2409283 2409291 2339587 2339595 2391600 2429764 2429772 2257726 2257734 2302861 2302888 2302896 2321475 2321483 2321491 2242572 2242573 2242574 612162 2374587 2374595 2245272 2245273 2245274 2279460 2279479 2279487 2294346 399302 312711 1913654 1913662 2245247  2297795 2407124 2405067 2295377 2295385 2295393 2167786 2229785 2305062 2302942 2302950 2302977 2355663 2355671 2355698 2403366 2403374 2403382 2348578 2340763 2340771 2341603 2341522 2438275 2438283 2384906 2384914 2384922 2424258 2424266 2424274 2363518 2363704 2363712 2364506 2364514 2247085 2247086 2247087 2248440  2248441 2258781 2258803 2258811 2241112 2241113 2241114 2241111 2363232 2363240 2363259 2444844 2444852 2265575 2265583 2448610 2361809 2361817 271330 12556  12564 586773 156728 209937 377937 430986 2274248 2274256 2274264 2274272 2224550 2224569 12599 454753 1987836 1987534  24708 24716 765996 2356422 2242987 15598 2234513 2234514 2229994 2242726 2307634 2307642 2307650 2307553 2307561 2307588 2421828 2421836 2421674 2421682 2421690 2443635 2443643 720933 720941 480304 244449 420336 480290 2435462 2435470 2279061 2279088 2279126 2248210 2287072  2155850 2248453 2190885 2190893 2239924 2239925 2239926 2099233 2162849 2446065 314552 2230444 2230443 2268493 2300451 2268507 2350459 2350467 2147521 2226804 2147548 2226812 2230036 2230037 1959352 1959360 2229516 2239214 2238471 2239476 2238469 2239474 2238470 2239475 2252945 2252953 2229704 2229705 2403412 2233562  2439611 2240294 2403420 2240295 2240297 2403439 2241283 1962639 889113 1962655 889105 795879 1959212 1962647 889091 1962663 889121 646148 587737 1959239  2403447 2241310 586714 2415089 1959220 733075 2275864 2275872 66123203 446564 446580 446572 446610 446602 446599 614416 1985981 1985949 2022249 1986813  632651 552275 1985957 552259 999717 5894 1934112 546348 612227 539244 552267 2455404 2455412 2455420 2455439 2455447  2455455 2425483 2425491 2378043 2378051 2380196 2380218 2380722 2380730 2365529 2365537 2397307 2333856 2333864 2333872 2416786 2416794 2416808 2303922 2388839 2388847 2443937 2443945 2403250  2403269 2403277 2417219 2417227 2417235 2389169 2389177 2389185 2245689 2251930 2276410 2294338 542911 1934090 612278 275409 514535 2412829 2271842 2378620 2378639 2084341 2085887 2230671 2230670 2231389 2242793 2242794 2246965 2284782 2284790 2343606 2343614 2353377 2353385 2378841 2378868 2334437 2238827 2334445  2230027 2385341 2385368 2406020 2406039 2230026 2220628 2231058 2423286 2388766 2388774 2326477 2326485 2326493 1985973 773654 1986821 2022230 632694 632678  1985965 13889 612359 2229519 2438658 2449765 808733 808741 2148765 2229656 2298279 2298287 2298295 2373270 2373289 2373297 2354349 2354357 2354365 2417189  2417197 2417200 21849 2273756 2273764 2273772 650935 2024292 2024306 2024217 2025248 2024314 2024322 2024241 2024225 2024268 2024233 2024284 2024276 1986791 612197 644358 612200 2024446 2024403 2025256 612189 2265435 2265443 21350 2244353 2245397 2377209 274127 542938 1934066 612235 542946 514551 2345854  2345862 2361264 2361272 2336316 2020734 2020742 2162822 2229517 2333554 2375842 237000 12602 12610 1987828 2224798 1987542 2224771 2419300 2419319 2419327 2419335 2419343 2419351 2229595 2229596 2378116 2378124 2246964 2307669 2307677 2307723 2307170 2307189 2307197 2345366 2345374 2345382 2417049 2417057 2417065  2374013 2374021 2374048 2294400 2284545 2284553 2313596 2236733 2236734 2242589 2223562 2303124 2303132 2303140 22303140 2354926 2354934 2354942 2306166 2306174 2306182 2357887 2357895 2357909 2357917 2357925 2236985 2238698 2236986 2449390 2449404 2316544 2314894 2314908 2312050 2312069 2312077 274119 539201 612219  612170 2339110 2339129 2439328 2269031 2269058 2375850 2375869 2375877 2389312 2389290 2389304 2273136 2273101 2273128 1900927 1900935 2242931 2242974 2301423 2301431 2301458 513644 2415968 2415976 2415984 2231095 2231096 2237531 2233999 2236543 2236548 2242095 2242096 2239081 2242783 2254719 2269589 2269597 2269600  2269619 2248008 2248009 2246820 2246821 2297906 2297914 2297922 2357453 2357461 2357488 2326329 2326337 2326345 2437899 1934082 612251 275417 2379767 2379775  2331519 2331527 13730 2245439 2245440 2245438 554820 6009 648094 1934074 2456575 2456583 2456591 2456605 2456613 2456621 2238103 1913670 1913689 2045710  2230475 2274914 2274922 2274930 2366347 2366355 2366363 2354144 2354152 2354160 312762 156663 431168 93033 178543 209872  2418002 2418010 2418029 2441829 2370921 2228920 2228939 2188902 2448599 2448602 1934104 612243 275425 2444933 2444941 2434121 2434148 2434156 1985930 1986085 632686 1986805 628301 723789 2351064 2351056 2449935 2449943 2320754 2320762 2320770 | [5, 6] |
| Cardiovascular Disease (CVD) | **Ischemic Heart Disease:**  2 or more physician visits with Angina ICD-9 code 413 plus 1 prescription within 1 year  OR  1 or more specialist visits with Angina ICD-9 code 413 plus one prescription within 1 year  OR  2 or more physician visits with two ICD-9 codes 410, 411, 412, 413, 414 in 1 year  OR  1 or more CABG, PCI/PCTA procedure code  OR  1 or more hospitalization with any ischemic heart disease diagnostic codes(s) | **ICD-9:**   - 410 (Acute myocardial infarction) - 411 (Other acute and subacute forms of ischaemic heart disease) - 412 (Old myocardial infarction) - 413 (Angina pectoris) - 414 (Other forms of chronic ischaemic heart disease)   **Canadian Classification of Diagnostic, Therapeutic, and Surgical Procedures (CCP):**   - **Coronary artery bypass surgery (CABG):** 4811, 4812, 4813, 4814, 4815, 4816, 4817, 4819. - **Percutaneous Coronary Intervention/Percutaneous Transluminal Coronary Angioplasty (PCI/PTCA):** 4802, 4803   **ICD-10:**   - I20 (Angina pectoris) - I21 (Acute myocardial infarction) - I22 (Subsequent myocardial infarction) - I23 (Certain current complications following acute myocardial infarction) - I24 (Other acute ischaemic heart diseases) - I25 (Chronic ischaemic heart disease)   **CABG:** 1IJ57LA, 1IJ57VS, 1IJ76.  **PCI/PTCA:** 1IJ50, 1IJ57G.  **DIN:**  3662 14214 15954 15962 37613 37621 104736 125849 202959 202967 202975 208973 243116 279536 299790 342734 441686 441694 442925 446661 446688 446696 458686 458694 476579 476587 476595 476609 525529 576174 584223 584258 584266I 590673 590681 590738 590746 602892 608785 614262 658812 660655 670944 677469 695726 725242 740721 749362 749370 749389 749397 754188 778869 786640 786667 786683 852384 860778 874213 874248 1911902 1911910 1911929 1913921 1926454 1926721 1927809 1946196 2011271 2041715 2041723 2042606 2042614 2042622 2044811 2046156 2046164 2058472 2125218 2126559 2145294 2145308 2145316 2162806 2163527 2163535 2164337 2182734 2213370 2230732 2230733 2230734 2231441 2238998 2243588 2272830 2301288 2393433 2407442 2407450 2407469 2407477 | [7] |
|  | **Transient Ischemic Attack (TIA):**  1 or more hospitalization with TIA diagnostic codes. | **ICD-9:**   - 435 (Transient cerebral ischemia)   **ICD-10:**   - H34.0 (Transient retinal artery occlusion) - G45.0 (Vertebro-basilar artery syndrome) - G45.1 (Carotid artery syndrome (hemispheric)) - G45.2 (Multiple and bilateral precerebral artery syndromes) - G45.3 (Amaurosis fugax) - G45.8 (Other transient cerebral ischemic attacks and related syndromes) - G45.9 (Transient cerebral ischemic attack, unspecified) | [8] |
|  | **Stroke:**  1 or more hospitalization with stroke diagnostic code(s). | **ICD-9:**   - 362.3 (Retinal vascular occlusion) - 430 (Subarachnoid hemorrhage) - 431 (Intracerebral hemorrhage) - 433.x1 (Occlusion and stenosis of precerebral arteries) - 434.x (Occlusion cerebral arteries) - 436 (Acute but ill-defined cerebrovascular disease)   **ICD-10:**   - H34.1 (Central retinal artery occlusion) - I60 (Subarachnoid hemorrhage) - I61 (Intracerebral haemorrhage) - I63 (Cerebral infarction) - I64 (Stroke, not specified as haemorrhage or infarction)   Excluding I63.6 (Cerebral infarction due to cerebral venous thrombosis)  Exclusions: any traumatic brain injury | [9] |
|  | **Heart Failure:**  1 or more hospitalization with heart failure diagnostic code(s)    OR  2 or more physician visits in one year with heart failure diagnostic code(s) | **ICD-9:**   - - 428 (Heart failure)   **ICD-10:**   - - I50 (Heart Failure) | [10] |
|  | **Acute Myocardial Infarction**:  1 or more hospitalization with an Acute Myocardial Infarction diagnostic code | **ICD-9:**   - 410 (Acute myocardial infarction)   **ICD-10:**   - I21 (Acute myocardial infarction) - I22 (Subsequent myocardial infarction) | [11] |
| Mood/Anxiety Disorders* | 1 or more hospitalization with a mood/anxiety diagnostic code    OR  2 or more medical visits with a mood/anxiety disorder diagnostic code within one year  *Note: Dissociative (conversion) disorders (F44), and Somatoform disorders (F45) were excluded | **ICD-9:**   - 296, 300,311, 50B   **ICD-10:**   - F30, F31, F32, F34, F38, F39, F40, F41, F42, F43, F48, F68   Excluding: F44, F45 | [12], and expert opinion |
| Chronic Kidney Diseases | **Chronic Kidney Disease (CKD)**  1 hospitalization  with CKD diagnostic code  OR  2 physician visits with CKD diagnostic code(s) in one year | **ICD-9:**   - 581, 582, 583, 585, 586, 587, 589   **ICD-10:**   - N01, N03, N04, N05, N06, N07, N18, N19, N26, N27 | [13] |
|  | **Dialysis**  9 or more physician visits within 90 days with dialysis fee code(s) | **Fee Codes:** 00308, 00323, 00350, 00351,00352, 00355, 00356, 00358, 00359, 00361, 00390, 33708, 33723, 33750, 33751, 33752, 33755, 33756, 33758, 33759, 33761, 33790 | [13] |
|  | **Kidney Transplant**  1 hospitalization for kidney transplant with procedure code(s) | **Canadian Classification of Health Interventions (CCI):** 675  **CCP:** 1PC85 | [13] |
| Chronic Obstructive Pulmonary Disease (COPD) | 1 or more hospitalization with COPD diagnostic code(s)  OR  2 or more physician visits with COPD diagnostic code(s) within one year | **ICD-9:**   - 491 (Chronic bronchitis) - 492 (Emphysema) - 496 (Chronic airways obstruction, not elsewhere classified)   **ICD-10:**   - J41 (Simple and mucopurulent chronic bronchitis) - J42 (Unspecified chronic bronchitis) - J43 (Emphysema) - J44 (Other chronic obstructive pulmonary disease)   **Physician Claims ICD-9:**   - 491 (Chronic bronchitis) - 492 (Emphysema) - 496 (Chronic airways obstruction, not elsewhere classified) | [14] |
| Asthma | 1 or more hospitalization with Asthma diagnostic code  OR  2 or more physician visits within one year with Asthma diagnostic code(s)  OR  1 or more physician visit with an Asthma diagnostic code and 2 or more Asthma prescriptions within 1 year. | **ICD-9:**   - 493 (Asthma)   **ICD-10:**   - J45 (Asthma)   **DIN:**  2401150 2236606 362611 454494 258350 362638 454508 270695 263761 2410265 2410273 2240835 2245126 2245127 2240836 2245125 2240837 2232570 2241407 872342 872334 2094959 2231787 2231784 2231783 2122154 2122146 2122138 2404311 2376938 2229862 2236783 2237602 2237224 2237225 589950 589942 379603 2427494 2427508 2427516 2239074 2236934 2236935 878715 2230249 1986872 254134 3859 249920 3891 2285606 2285614 2285592 128902 101036 12033 12041 267155 392707 535664 582662 392723 535672 582654 497193 497207 54836 14923 14931 92940 178497 178500 441724 441732 511692 2231431 2126222 2231494 2243827 2374609 2237134 2237135 2259842 2259850 2259869 2259877 2261324 2236931 2236932 2236933 2261332 2014270 491179 2014289 593230 792934 2231135 2231136 2231245 2231244 2354977 2354985 2373947 92942 2069571 2208229 2208237 2208245 2091186 2046113 575151 629480 454559 2440350 634549 817228 2229099 2377608 2377616 2046741 790419 2245669 2231488 2231678 2243828 2146843 2146851 2266393 2358395 2358409 692689 692697 692700 2446561 2446588 604380 47171 604399 2243595 2243596 2438690 2231430 2048760 731439 576158 2247686 2026759 1950681 2401274 2422867 2422875 2379376 2379341 2379368 1978918 1978926 634530 814091 852074 851752 851760 466409 556742 547115 2242029 2242030 2389517 2402793 2402807 2243789 2097141 2097168 2097176 2336685 2336669 2336677 2152568 851841 860808 1986864 2239365 2239366 2244914 574937 574945 2398796 2398818 2398826 405310 451282 346071 2259583 2259591 2213710 2213729 828521 828548 897353 768707 2215055 2079976 2216531 893633 334243 2215039 2215047 545325 1949993 545333 1950002 541389 2006383 371807 522589 454796 2056712 2056704 2445735 2408872 2444186 818739 444774 335355 335363 786616 476366 380660 226718 328944 812463 2154382 2164434 2164442 2230005 2237113 2230006 2236786 2237114 2419858 2245547 2245548 2148617 2215616 2215624 2215632 2237256 637793 637807 2228297 2330385 2330393 2358611 2328593 2154412 2211742 2136139 2214261 2231129 2136147 2238216 2238217 2243602 2247997 1926586 1926594 226696 476374 476382 202940 476390 503436 536709 476404 31259 476412 31267 483591 467006 2259486 2259494 2276348 2163721 2419106 2231675 451665 451673 18228 46752 2376679 2376687 2376695 2216930 2216957 2139324 2216949 2439530 2148633 627410 286214 529095 704482 529109 1926608 1926616 600024 600032 600040 778893 551430 609013 551414 609021 551422 692778 692751 442275 442283 442291 2245385 2245386 2192675 2272695 2216221 2355507 2355515 2355523 1926934 2173360 2230085 2230086 2230087 704016 2360101 2360128 355003 631698 631701 599905 460982 530603 704466 704490 530581 273066 281905 367869 2243646 2213648 2213656 2213621 2213664 2237244 2237245 2237246 2237247 2174707 2174715 2174723 2174693 2244291 2244292 2244293 2213605 2213613 2213583 2213591 2174731 2174758 2174766 2174774 2230898 205796 2238795 2246066 2219468 555649 460990 461008 722065 713422 713430 713406 713414 1966219 395218 461687 461709 791652 461695 545732 502014 1966243 1966251 1966278 1966286 1966235 1966227 828718 640093 828726 828742 261203 599042 599050 599077 156701 488070 532223 261181 307548 1926659 1926640 704024 534609 261238 638641 2437619 2437627 2443007 2264609 2231455 2231456 2237189 2153408 868450 2391422 2442353 2442361 2399865 2399873 2399997 2241119 2241120 2084333 2237414 2408627 2408635 2408643 2230446 2379317 2379325 2379333 2379821 2379848 2379856 2382458 2382466 2382474 2410516 2391139 704032 2230543 766038 2418282 2396793 2396807 2396815 2014165 2014181 738875 738883 2427605 2427613 2427621 2214997 2215004 832766 832758 2243115 2241497 602914 2213443 2213435 2213451 602906 894249 303569 2213478 253081 867179 2063689 894257 602922 894265 667242 2213419 2213427 2391112 2391104 2391120 2379236 2239131 2368226 2380749 2380757 2232987 500895 2049082 2210479 2326450 874086 620955 620963 458708 458716 565377 2376822 2376830 2376849 2231671 2228238 2231785 2236565 2165368 2165376 2213400 2022125 897345 1945203 2212390 670790 2035421 334227 2213486 1947222 2212315 2212323 622060 1938878 2213215 622079 1938851 361135 1961039 332267 1932691 782351 782378 2260565 2361744 2361752 2361760 | [15] |
| Rheumatoid Arthritis (RA) | 2 or more physician visits 61─720 days apart with rheumatoid arthritis diagnostic codes (ICD-9: 714). Exclusions apply – see notes below:  **Exclude:**  (Exclusion Rule 1) patients who have seen a rheumatologist, but who have never been diagnosed with rheumatoid arthritis by the rheumatologist;  (Exclusion Rule 2) patients who had two rheumatologist visits coded with other inflammatory arthritis diagnostic codes (ICD-9: 696, 710, 711, 720) at any point subsequent to their last RA visit. | **ICD-9:**   - 714 (Rheumatoid Arthritis and other inflammatory polyarthropathies)   **Exclude:**  As per Exclusion Rule 1-   - 714 (Rheumatoid arthritis)   As per Exclusion Rule 2 -   - 696 (Psoriasis and similar disorders) - 710 (Diffuse diseases of connective tissue) - 711 (Arthropathy associated with infections) - 720 (Ankylosing spondylitis and other inflammatory spondylopathies) | [13] |
| Systemic lupus erythematosus | 2 non-rheumatology physician visits, at least 2 months apart, within a 2-year period), with lupus diagnostic codes  OR  1 physician visit to a rheumatologist with a lupus diagnostic code  OR  1 hospitalization with lupus diagnostic code(s)  AND  excluding individuals with at least two visits, at least 2 months apart, subsequent to the second visit, with two identical diagnoses of other inflammatory arthritis and connective tissue diseases (RA, psoriatic arthritis, ankylosing spondylitis, and other spondyloarthropathies, scleroderma Sjögren’s syndrome, dermatomyositis, polymyositis, other connective tissue diseases, primary systemic vasculitis)  AND  excluding individuals where a lupus diagnosis by a non-rheumatologist was not confirmed if/when the individual saw a rheumatologist. | **ICD-9:**   - 710.0. (Systemic lupus erythematosus)   **ICD-10:**   - M32 (Drug-induced systemic lupus erythematosus) - M32.1 (Systemic lupus erythematosus with organ or system involvement) - M32.8 (Other forms of systemic lupus erythematosus) - M32.9 (Systemic lupus erythematosus, unspecified)   **Excluding the following codes:**  **Rheumatoid arthritis**   - **ICD-9:** 714.0, 714.1, 714.2. - **ICD-10:** MO5 – MO5.9, MO6.0, MO6.8, MO6.9.   **Psoriatic arthritis**   - **ICD-9:** 696.0. - **ICD-10:** L40.5   **Ankylosing spondylitis**   - **ICD-9:** 720.0. - **ICD-10:** M45   **Other Spondyloarthropathies**   - **ICD-9:** 720.1, 720.2, 720.8, 720.9. - **ICD-10:** M46.0, M46.1, M46.2, M46.3, M46.4, M46.5 M46.8, M46.9   **Scleroderma**   - **ICD-9:** 710.1. - **ICD-10:** M34   **Sjögren’s syndrome**   - **ICD-9:** 710.2. - **ICD-10:** M35.0   **Dermatomyositis**   - **ICD-9:** 710.3. - **ICD-10:** M33.1, M33.9)   **Polymyositis**   - **ICD-9:** 710.4. - **ICD-10:** M33.2   **Other connective tissue diseases**   - **ICD-9:**710.5, 710.8, 710.9. - **ICD-10:** M35.1, M35.2, M35.8, M35.9   **Primary systemic vasculitis:**   - **ICD-9:** 446.0, 446.2, 446.4, 446.5, 446.7, 447.6. - **ICD-10:** D69.0, M31.0, M30.0, M31.3, M31.4, M31.5, M31.6, M31.7, M31.8, M31.9) | [16] |
| Inflammatory Bowel Disease (IBD) | **Crohn's Disease (CD)**  Individual registered within the healthcare system for more than 2 years: 5 or more hospitalizations or physician claims with CD diagnostic code(s)  Individual registered within the healthcare system for less than 2 years: 3 or more hospitalizations or physician claims with CD diagnostic code(s) | **ICD-9:**   - 555 (Regional Enteritis)   **ICD-10:**   - K50 (Crohn's disease [regional enteritis]) | [17] |
|  | **Ulcerative Colitis (UC)**  Individual registered within the healthcare system for more than 2 years: 5 or more hospitalizations or physician claims with UC diagnostic code(s)  Individual registered within the healthcare system for less than 2 years: 3 or more hospitalizations or physician claims with UC diagnostic code(s) | **ICD-9:**   - 556 (Idiopathic Proctocolitis)   **ICD-10:**   - K51 (Ulcerative colitis) | [17] |
| Hypertension | 1 hospitalization with a hypertension diagnostic code  OR  2 physician visits in one year with hypertension diagnostic codes(s) | **ICD-9:**   - 401, 402, 403, 404, 405   **ICD-10:**   - I10, I11, I12, I13, I15 | [13, 18] |
| Schizophrenia | 1 hospitalization with a schizophrenia diagnostic code  OR  2 physician visits with schizophrenia diagnostic code(s) at least 30 days apart in two years | **ICD-9:**   - 295   **ICD-10:**   - F20, F21, F23, F25 | [19] |
| Substance use disorder (SUD) | 1 or more hospitalization with a SUD diagnostic code  OR  2 or more physician visits with SUD diagnostic code(s) within one year | **ICD-9:**   - 291, 292, 303, 304, 305,   **ICD-10:**   - F10, F11, F12, F13, F14, F15, F16, F17, F18, F19 | [20] |
| Traumatic Brain Injury | 1 traumatic brain injury diagnostic code | **ICD-9:** 800, 801, 802, 803, 804, 850, 851, 852, 853, 854, V57  **ICD 10:** S02.x, S06.x, Z50 | [21] |
| Delirium | 1 or more physician visit with an episodic delirium diagnostic code  OR    1 or more hospitalization with an episodic delirium diagnostic code | **ICD-9:**   - 290.11, 290.12, 290.13 290.2, 290.3, 290.41, 290.42, 290.43 290.8, 290.9, 291.0, 292, 292.11, 292.12 292.2, 292.81, 292.82, 293.0, 293.1, 293.81, 293.82, 293.83, 293.84,293.8, 293.9, 348.3, 348.31, 348.39, 349.82, 780.09, 780.02, 780.97   **ICD-10:**   - F05, F10121, F10221, F10231, F10921, F11121, F11221, F11921, F12121, F12221, F12921, F13121, F13221, F13231, F13921, F13931, F14121, F14221, F14921, F15121, F15221, F15921, F16121, F16221, F16921, F18121, F18221, F18921, F19121, F19231, F19921, F19931, F10.4, F11.4, F12.4, F13.4, F14.4, F15.4, F16.4, F17.4 F18.4, F19.4A81.2, E51.2, G0430, G0431, G0432, G0439, G92, G9340, G9341, G9349, I67.3, I67.4, I6783, J10.8, J11.8 | [22], and expert opinion |
| Non-AIDS-defining cancers (NADC) | 1 hospitalization with a NADC diagnostic code  OR  2 Physician visits in 1Y with NADC diagnostic codes | **ICD-9:**   - 140: Malignant Neoplasm of the Lip - 141: Malignant Neoplasm of Tongue - 142: Malignant Neoplasm of Major Salivary Glands - 143: Malignant Neoplasm of Gum - 144: Malignant Neoplasm of Floor of Mouth - 145: Malignant Neoplasm of Other and Unspecified Parts of Mouth - 146: Malignant Neoplasm of Oropharynx - 147: Malignant Neoplasm of Nasopharynx - 148: Malignant Neoplasm of Hypopharynx - 149: Malignant Neoplasm of Other and Ill-defined Sites within the Lip, Oral Cavity and Pharynx - 150: Malignant Neoplasm of Esophagus - 151: Malignant Neoplasm of Stomach - 152: Malignant Neoplasm of Small Intestine, Including Duodenum - 153: Malignant Neoplasm of Colon - 154: Malignant Neoplasm of Rectum, Rectosigmoid Junction and Anus. - 155: Malignant Neoplasm of Liver and Intrahepatic Bile Ducts - 156: Malignant Neoplasm of Gallbladder and Extrahepatic Bile Ducts - 157: Malignant Neoplasm of Pancreas - 158: Malignant Neoplasm of Retroperitoneum and Peritoneum - 159: Malignant Neoplasm of Other and Ill-Defined Sites Within the Digestive Organs and Peritoneum - 160: Malignant Neoplasm of Nasal Cavities, Middle Ear and Accessory Sinuses - 161: Malignant Neoplasm of Larynx - 162: Malignant Neoplasm of Trachea, Bronchus and Lung - 163: Malignant Neoplasm of Pleura - 164: Malignant Neoplasm of Thymus, Heart and Mediastinum - 165: Malignant Neoplasm of Other and Ill-Defined Sites Within the Respiratory System and Intrathoracic Organs - 170: Malignant Neoplasm of Bone and Articular Cartilage - 171: Malignant Neoplasm of Connective and Other Soft Tissue - 172: Malignant Melanoma of Skin - 173: Other Malignant Neoplasm of Skin - 174: Malignant Neoplasm of Female Breast - 175: Malignant Neoplasm of Male Breast - 179: Malignant Neoplasm of Uterus, Part Unspecified - 181: Malignant Neoplasm of Placenta - 182: Malignant Neoplasm of Body of Uterus - 183: Malignant Neoplasm of Ovary and Other Uterine Adnexa - 184: Malignant Neoplasm of Other and Unspecified Female Genital Organs - 185: Malignant Neoplasm of Prostate - 186: Malignant Neoplasm of Testis - 187: Malignant Neoplasm of Penis and Other Male Genital Organs - 188: Malignant Neoplasm of Bladder - 189: Malignant Neoplasm of Kidney and Other and Unspecified Urinary Organs - 190: Malignant Neoplasm of Eye - 191: Malignant Neoplasm of Brain - 192: Malignant Neoplasm of Other and Unspecified Parts of Nervous System - 193: Malignant Neoplasm of Thyroid Gland - 194: Malignant Neoplasm of Other Endocrine Glands and Related Structures - 195: Malignant Neoplasm of Other and Ill-Defined Sites - 196 Secondary and unspecified malignant neoplasm of lymph nodes - 197 Secondary malignant neoplasm of respiratory and digestive systems - 198 Secondary malignant neoplasm of other specified sites - 199 Malignant neoplasms without specification of site - 200: Lymphosarcoma and Reticulosarcoma - 201: Hodgkin's Disease - 202: Other Malignant Neoplasm of Lymphoid and Histiocytic Tissue - 203: Multiple Myeloma and Immunoproliferative Neoplasms - 204: Lymphoid Leukaemia - 205: Myeloid Leukaemia - 206: Monocytic Leukaemia - 207: Other Specified Leukaemia - 208: Leukaemia of Unspecified Cell Type - 210: Benign Neoplasm of Lip, Oral Cavity and Pharynx - 211: Benign Neoplasm of Other Parts of Digestive System - 212: Benign Neoplasm of Respiratory and Intrathoracic Organs - 213: Benign Neoplasm of Bone and Articular Cartilage - 214: Lipoma - 215: Other Benign Neoplasm of Connective and Other Soft Tissue - 216: Benign Neoplasm of Skin - 217: Benign Neoplasm of Breast - 218: Uterine Leiomyoma - 219: Other Benign Neoplasm of Uterus - 220: Benign Neoplasm of Ovary - 221: Benign Neoplasm of Other Female Genital Organs - 222: Benign Neoplasm of Male Genital Organs - 223: Benign Neoplasm of Kidney and Other Urinary Organs - 224: Benign Neoplasm of Eye - 225: Benign Neoplasm of Brain and Other Parts of Nervous System - 226: Benign Neoplasm of Thyroid Gland - 227: Benign Neoplasm of Other Endocrine Glands and Related Structures - 228: Haemangioma and Lymphangioma, Any Site - 229: Benign Neoplasm of Other and Unspecified Sites - 230: Carcinoma in Situ of Digestive Organs - 231: Carcinoma in Situ of Respiratory System - 232: Carcinoma in Situ of Skin - 233: Carcinoma in Situ of Breast and Genitourinary System - 234: Carcinoma in Situ of Other and Unspecified Sites - 235: Neoplasm of Uncertain Behaviour of Digestive and Respiratory Systems - 236: Neoplasm of Uncertain Behaviour of Genitourinary Organs - 237: Neoplasm of Uncertain Behaviour of Endocrine Glands and Nervous System - 238: Neoplasm of Uncertain Behaviour of Other and Unspecified Sites and Tissues - 239: Neoplasm of Unspecified Nature   **ICD-10**   - C00: Malignant Neoplasm of External Upper Lip - C01- C02: Malignant Neoplasm of tongue - C03: Malignant Neoplasm of Gum - C04: Malignant Neoplasm of Floor of Mouth - C05: Malignant Neoplasm of Palate - C06: Malignant Neoplasm of Cheek Mucosa - C07 - C08: Malignant Neoplasm of Salivary Glands - C09: Malignant Neoplasm of Tonsils - C12: Malignant Neoplasm of Pyriform sinus - C13: Malignant Neoplasm of Hypopharynx - C14: Malignant Neoplasm of Pharynx Unspecified - C10: Malignant Neoplasm of Oropharynx - C11: Malignant Neoplasm of Nasopharynx - C15: Malignant Neoplasm of Esophagus - C16: Malignant Neoplasm of the Stomach - C17: Malignant Neoplasm of the small intestine (Duodenum, Jejunum and Ileum) - C18, C26.0: Malignant Neoplasm of the Large intestine (Colon) - C19: Malignant Neoplasm of the Rectosigmoid Junction - C20: Malignant Neoplasm of the Rectum - C21: Malignant Neoplasm of the Anus - C22.0: Liver Cell Carcinoma - C22.1: Intrahepatic Bile Duct Carcinoma - C22.2: Hepatoblastoma - C22.3: Angiosarcoma of Liver - C22.4: Other Sarcomas of Liver - C22.7: Other Specified Carcinomas of Liver - C22.9: Malignant Neoplasm of Liver Unspecified - C23: Malignant Neoplasm of the gall bladder - C24: Malignant Neoplasm of the Biliary Tract - C25: Malignant Neoplasm of the Pancreas - C26.0: Malignant Neoplasm of Intestinal Tract, Unspecified - C26.2 - C26.9: Other Malignant Neoplasm of the digestive system and unspecified - C30: Malignant Neoplasm of Nasal Cavity and Ears - C31: Malignant Neoplasm of Sinuses - C32: Malignant Neoplasm of the Larynx - C33: Malignant Neoplasm of Trachea - C34: Malignant Neoplasm of Bronchus and Lung - C38.0: Malignant Neoplasm of Heart - C38.1: Malignant Neoplasm of Anterior Mediastinum - C38.2: Malignant Neoplasm of Posterior Mediastinum - C38.3: Malignant Neoplasm Mediastinum, Unspecified - C38.4: Malignant Neoplasm of Pleura - C38.8: Overlapping Malignant Lesion of Heart, Mediastinum and Pleura - C39: Other Malignant Neoplasm of the respiratory system, Unspecified - C40 - C41: Malignant Neoplasm of the Bones - C43: Malignant Melanoma - C45: Mesothelioma - C48: Malignant of the Retroperitoneum - C47: Malignant Neoplasm of Peripheral Nerves, Autonomic Nervous System - C49: Malignant Neoplasm of Connective and Soft Tissue - C50: Malignant Neoplasm of the Breast - C51: Malignant Neoplasm of the Labia, Clitoris and Vulva - C52: Malignant Neoplasm of Vagina - C57: Malignant Neoplasm of the Fallopian Tubes, Round Ligament, Parametrium and Unspecified - C58: Malignant Neoplasm of Placenta - C60: Malignant Neoplasm of the Penis - C63: Other Malignant Neoplasm of the male genital organs and Unspecified - C54: Malignant Neoplasm of the body of the Uterus - C55: Malignant Neoplasm of the Uterus, part Unspecified - C56: Malignant Neoplasm of the Ovary - C61: Malignant Neoplasm of the Prostate - C62: Malignant Neoplasm of the Testis - C64: Malignant Neoplasm of the Kidney, except Renal Pelvis - C65: Malignant Neoplasm of Renal Pelvis - C66: Malignant Neoplasm of Ureter - C68: Malignant Neoplasm of Urethra - C67: Malignant Neoplasm of the Bladder (invasive, in situ) - C69: Malignant Neoplasm of the Eye - C70: Malignant Neoplasm of Meninges - C71: Malignant Neoplasm of Cerebrum and Lobes - C72: Malignant Neoplasm of Central Nervous System - C73: Malignant Neoplasm of Thyroid Gland - C37, C74 - C75: Malignant Neoplasm of other endocrine glands - C81: Hodgkin's Lymphoma - C90.0, C90.2: Multiple Myeloma - C91 - C95, C90.1: Leukemia - C26.1, C44, C76–C80, C88, C96.0–C96.2, C96.7–C96.9, C97: All Other and Unspecified Cancers | [23] |
| Cirrhosis | 1 hospitalization with a cirrhosis diagnostic code    OR    2 physician visits with cirrhosis diagnostic code(s) in one year | - **ICD-9 (MSP):** 571 - **ICD-9 (Hospital):** 4561, 5712, 5715 - **ICD-10 (Hospital):** I859, I982, K703, K717, K746 | [24, 25] |
| Decompensated Liver Cirrhosis | 1 hospitalization with a with a decompensated cirrhosis diagnostic code    OR    2 physician visits with decompensated cirrhosis diagnostic code(s) in one year | **ICD-9:**   - 456.0, 456.2, 572.2, 572.3, 572.4, 782.4, 789.5   **ICD-10:**   - I85.0, I86.4, I98.20, I98.3, K72.1, K72.9, K76.6, K76.7, R17, R18 | [24, 25] |
| Chronic liver disease (CLD) | 1 hospitalization with a CLD diagnostic code    OR    2 physician visits with CLD diagnostic code(s) in one year | **ICD-9:**   - 070.2, 070.3, 070.41, 070.44, 070.51, 070.54, 070.7, 275.0, 573.3   **ICD-10:**   - B16, B17.10, B17.11, B18.0, B18.1, B18.2, B19.10, B19.11, B19.20, B19.21, E83.11, K71.6, K75.9 | [24, 25] |
| Transplant | 1 transplant diagnostic code | - **ICD9:** V42 - **ICD10:** Z94 - **CCI:** 85 | Expert Opinion |

**Note.** ICD-9/10: International Classification of Diseases (Ninth and Tenth revisions)

# **Figure S1.** Flowchart describing eligibility criteria and derivation of the final analytical samples of people living with HIV (PLWH) and people living without HIV (PLWoH).


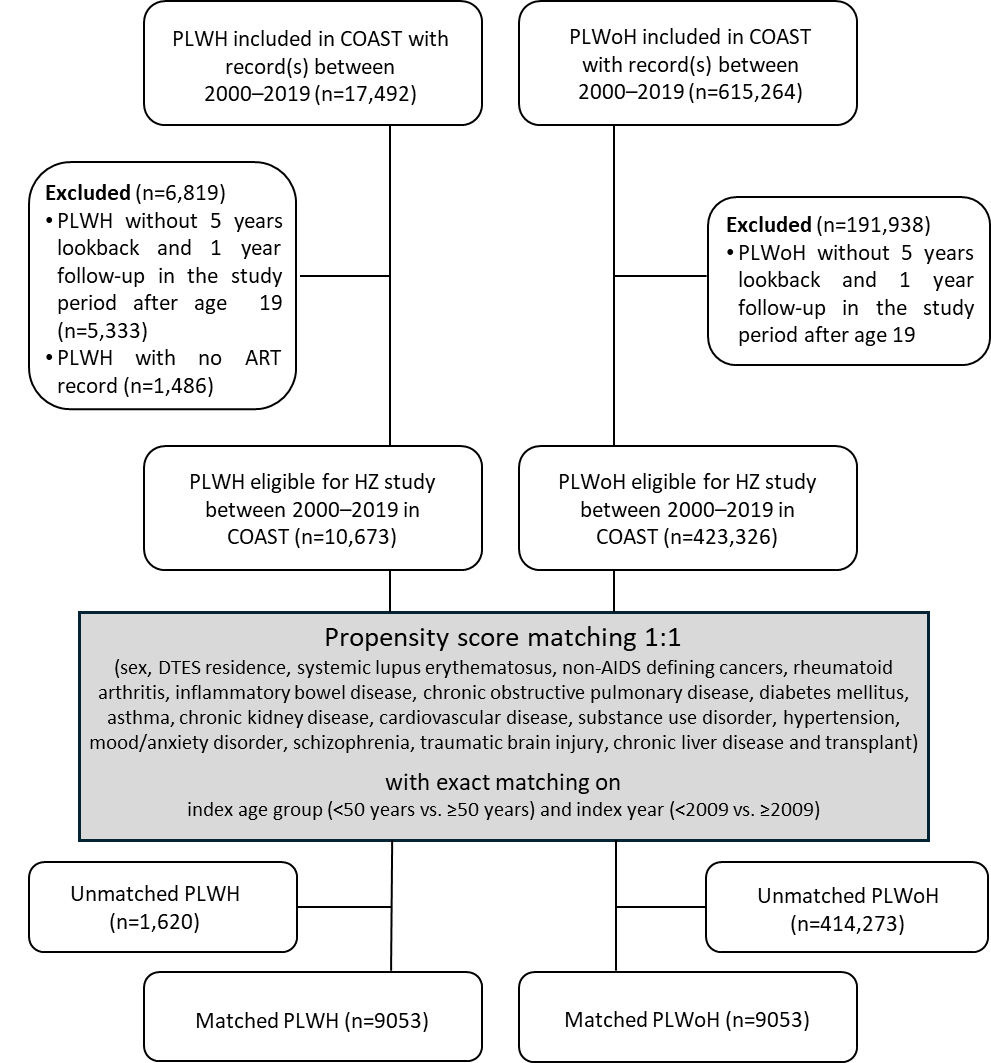


**Note.** COAST: Comparative Outcomes and Service Utilization Trends study cohort; DTES: Vancouver‘s Downtown Eastside; HZ: herpes zoster.

# **Table S2.** Descriptive characteristics of all eligible participants before propensity score matching, by HIV status.

| **Variable** | **PLWH** | **PLWoH** | **P-value** |
| --- | --- | --- | --- |
|  | **n = 10673**  n(%) | **n = 423326**  n(%) |  |
| Index Age (years) | | | |
| <50 | 8819(3) | 303439(97) | <0.0001 |
| ≥50 | 1854(2) | 119887(98) |  |
| Index Year |  |  |  |
| <2009 | 7513(2) | 364204(98) | <0.0001 |
| ≥2009 | 3160(5) | 59122(95) |  |
| Sex |  |  |  |
| Male | 8835(4) | 212467(96) | <0.0001 |
| Female | 1838(1) | 210859(99) |  |
| Downtown Eastside residence (Index date) | |  |  |
| No | 10135(2) | 421948(98) | <0.0001 |
| Yes | 538(28) | 1378(72) |  |
| Downtown Eastside residence (End of follow-up) | |  |  |
| No | 9950(2) | 421261(98) | <0.0001 |
| Yes | 723(26) | 2065(74) |  |
| Herpes zoster infection (End of follow-up) |  |  |  |
| No | 9268(2) | 391734(98) | <0.0001 |
| Yes | 1405(4) | 31592(96) |  |
| Herpes zoster infection (End of follow up) |  |  |  |
| No | 9268(2) | 391734(98) | <0.0001 |
| 1 time | 1225(4) | 29090(96) |  |
| 2 or more times | 180(7) | 2502(93) |  |
| Systemic lupus erythematosus (Index date) |  |  |  |
| No | 10666(20) | 423007(98) | 0.7160 |
| Yes | 7(21) | 319(98) |  |
| Systemic lupus erythematosus (End of follow-up) | |  |  |
| No | 10650(2) | 422235(98) | 0.3900 |
| Yes | 23(2) | 1091(98) |  |
| Non-AIDS-defining cancer (Index date) | |  |  |
| No | 9236(2) | 379614(98) | <0.0001 |
| Yes | 1437(3) | 43712(97) |  |
| Non-AIDS-defining cancer (End of follow-up) | |  |  |
| No | 7045(2) | 288902(98) | <0.0001 |
| Yes | 3628(3) | 134424(97) |  |
| Rheumatoid arthritis (Index date) | |  |  |
| No | 10620(2) | 420761(98) | 0.1570 |
| Yes | 53(2) | 2565(98) |  |
| Rheumatoid arthritis (End of follow-up) | |  |  |
| No | 10550(2) | 415756(98) | <0.0001 |
| Yes | 123(2) | 7570(98) |  |
| Inflammatory bowel disease (Index date) | |  |  |
| No | 10645(2) | 422325(98) | 0.5764 |
| Yes | 28(3) | 1001(97) |  |
| Inflammatory bowel disease (End of follow-up) | |  |  |
| No | 10627(2) | 421051(98) | 0.1280 |
| Yes | 46(2) | 2275(98) |  |
| Chronic obstructive pulmonary disease (Index date) | |  |  |
| No | 10460(2) | 417342(98) | <0.0001 |
| Yes | 213(3) | 5984(97) |  |
| Chronic obstructive pulmonary disease (End of follow-up) | |  |  |
| No | 9441(2) | 391788(98) | <0.0001 |
| Yes | 1232(4) | 31538(96) |  |
| Diabetes melllitus (Index date) |  |  |  |
| No | 10278(2) | 407169(98) | 0.6529 |
| Yes | 395(2) | 16157(98) |  |
| Diabetes mellitus (End of follow-up) |  |  |  |
| No | 9223(2) | 360638(98) | <0.0001 |
| Yes | 1450(2) | 62643(98) |  |
| Asthma (Index date) |  |  |  |
| No | 9818(2) | 402997(98) | <0.0001 |
| Yes | 855(4) | 20329(96) |  |
| Asthma (End of follow-up) |  |  |  |
| No | 8961(2) | 374993(98) | <0.0001 |
| Yes | 1712(3) | 48333(97) |  |
| Chronic kidney disease (Index date) | |  |  |
| No | 10405(2) | 420636(98) | <0.0001 |
| Yes | 268(9) | 2690(91) |  |
| Chronic kidney disease (End of follow-up) | |  |  |
| No | 9014(2) | 392804(98) | <0.0001 |
| Yes | 1659(5) | 30522(95) |  |
| Cardiovascular disease (Index date) | |  |  |
| No | 10269(2) | 400527(98) | <0.0001 |
| Yes | 404(2) | 22799(98) |  |
| Cardiovascular disease (End of follow-up) | |  |  |
| No | 8987(3) | 345449(97) | <0.0001 |
| Yes | 1686(2) | 77877(98) |  |
| Substance use disorder (Index date) |  |  |  |
| No | 7492(2) | 413243(98) | <0.0001 |
| Yes | 3181(24) | 10083(76) |  |
| Substance use disorder (End of follow-up) |  |  |  |
| No | 5826(1) | 394602(99) | <0.0001 |
| Yes | 4847(14) | 28724(86) |  |
| Hypertension (Index date) |  |  |  |
| No | 9993(3) | 377611(97) | <0.0001 |
| Yes | 680(1) | 45715(99) |  |
| Hypertension (End of follow-up) |  |  |  |
| No | 8189(3) | 284219(97) | <0.0001 |
| Yes | 2484(2) | 139107(98) |  |
| Mood/anxiety disorders (Index date) |  |  |  |
| No | 5731(2) | 341632(98) | <0.0001 |
| Yes | 4942(6) | 81694(94) |  |
| Mood/anxiety disorders (End of follow-up) | |  |  |
| No | 3687(1) | 255926(99) | <0.0001 |
| Yes | 6986(4) | 167400(96) |  |
| Schizophrenia (Index date) |  |  |  |
| No | 10366(2) | 420923(98) | <0.0001 |
| Yes | 307(11) | 2403(89) |  |
| Schizophrenia (End of follow-up) | |  |  |
| No | 9928(2) | 417483(98) | <0.0001 |
| Yes | 745(11) | 5843(89) |  |
| Traumatic brain injury (Index date) |  |  |  |
| No | 10047(2) | 411602(98) | <0.0001 |
| Yes | 626(5) | 11724(95) |  |
| Traumatic brain injury (End of follow-up) |  |  |  |
| No | 9007(2) | 378952(98) | <0.0001 |
| Yes | 1666(4) | 44374(96) |  |
| Chronic liver disease (Index date) |  |  |  |
| No | 9836(2) | 421131(98) | <0.0001 |
| Yes | 837(28) | 2195(72) |  |
| Chronic liver disease (End of follow-up) |  |  |  |
| No | 7708(2) | 409985(98) | <0.0001 |
| Yes | 2965(18) | 13341(82) |  |
| Transplant operation (Index date) |  |  |  |
| No | 10654(2) | 422805(98) | 0.0924 |
| Yes | 19(4) | 521(96) |  |
| Transplant operation (End of follow-up) |  |  |  |
| No | 10557(2) | 418951(98) | 0.5920 |
| Yes | 116(3) | 4375(97) |  |
| **Continuous Variables** | | | |
| Age (years)(Index date) |  |  |  |
| Median | 39 | 37 | <0.0001 |
| 25^th^–75^th^ percentiles | 33–46 | 24–52 |  |
| Age (years)(End of follow-up) |  |  |  |
| Median | 52 | 55 | <0.0001 |
| 25^th^­–75^th^ percentiles | 44–59 | 39–69 |  |

**Note.**  PLWH: People living with HIV/AIDS; PLWoH: People living without HIV/AIDS; p-value significance is set at 0.05; row percentages are shown.

# **Text S2.** Propensity Score Matching Overview and Diagnostic Assessment

We first present the results of our propensity score matching (PSM) procedure, which was introduced to reduce baseline differences between people living with HIV (PLWH) and people living without HIV (PLWoH). The substantial baseline imbalance observed across sociodemographic and clinical characteristics (Table S2) required a structured approach to ensure that comparisons between groups were valid and interpretable. This section summarises the matching strategy, the diagnostic assessments, and the characteristics of individuals retained and excluded after matching.

**1. Baseline Imbalance Before Matching**

Table S2 illustrates the pronounced asymmetry between PLWH and PLWoH at baseline. PLWH had markedly higher prevalence of Downtown Eastside residence, substance use disorder, schizophrenia, mood and anxiety disorders, traumatic brain injury, and chronic liver disease (all p <0.0001). These differences persisted—and in some cases widened—by the end of follow-up. The number and magnitude of these imbalances indicated substantial confounding, which directly informed the selection of covariates included in the propensity score model.

**2. Propensity Score Estimation and Matching Procedure**

We estimated propensity scores using logistic regression with HIV serostatus as the dependent variable. Baseline covariates included age group, sex, calendar era, Downtown Eastside residence, substance use disorder, mental health diagnoses, and a comprehensive list of chronic conditions. Exact matching was applied on index age group (<50 vs. ≥50 years) and index year (before vs. after 2009), so we could conduct the stratified analyses. We used 1:1 nearest-neighbour matching without replacement and a caliper of 0.3 standard deviations of the logit of the propensity score.

**3. Diagnostic Results: Success of Matching**

Propensity score distributions before and after matching (Figure S2) demonstrated excellent improvement in common support across groups. Standardised mean differences (SMDs) for all matched covariates were substantially reduced, with all post-match SMDs falling well below 0.10 (Figure S3). This confirms that covariate balance was achieved across all major sociodemographic and clinical domains, when comparing PLWH and PLWoH.

**Figure S2.** Propensity score distributions before and after matching

(a) Before PSM


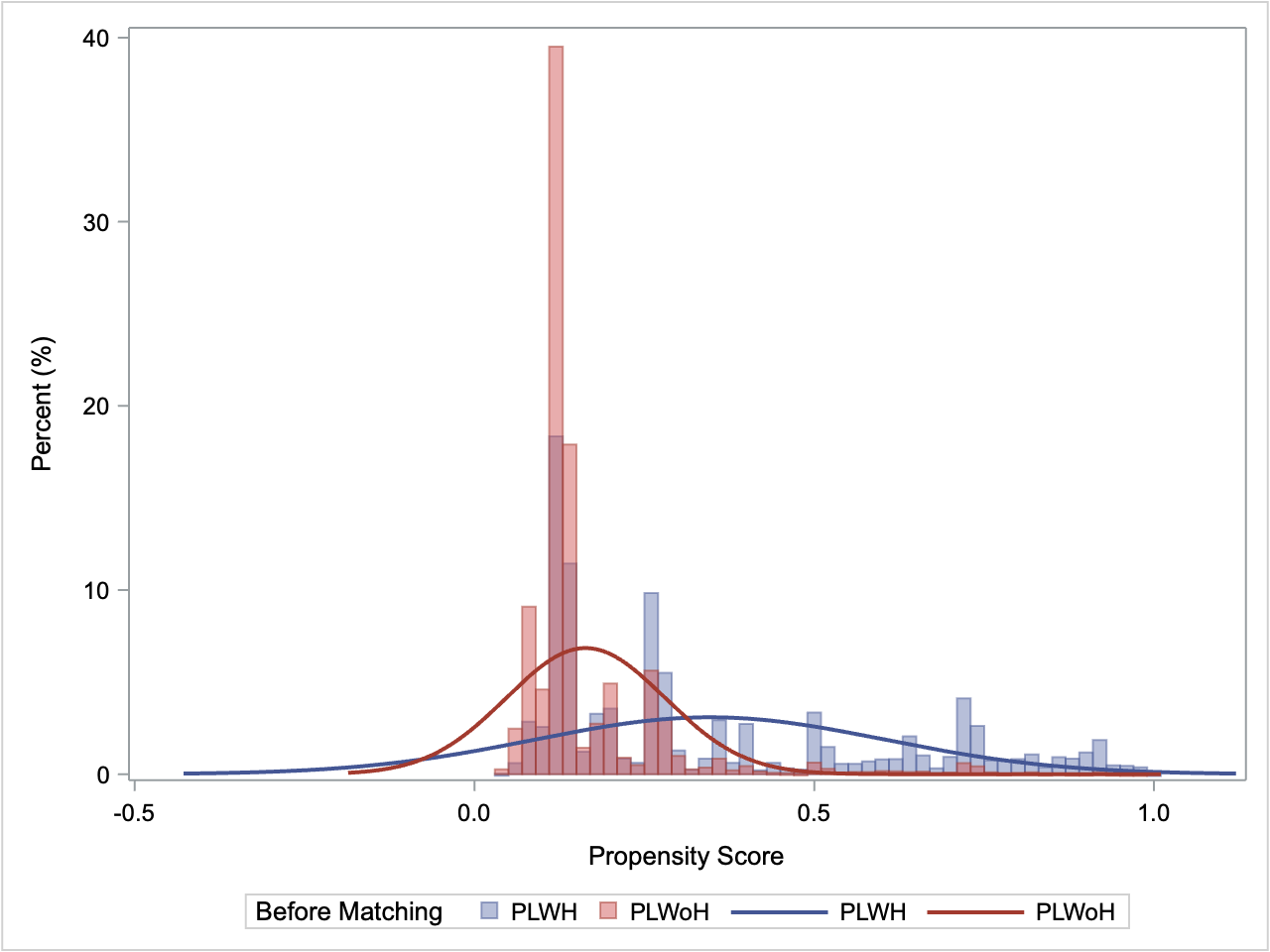


(b) After PSM


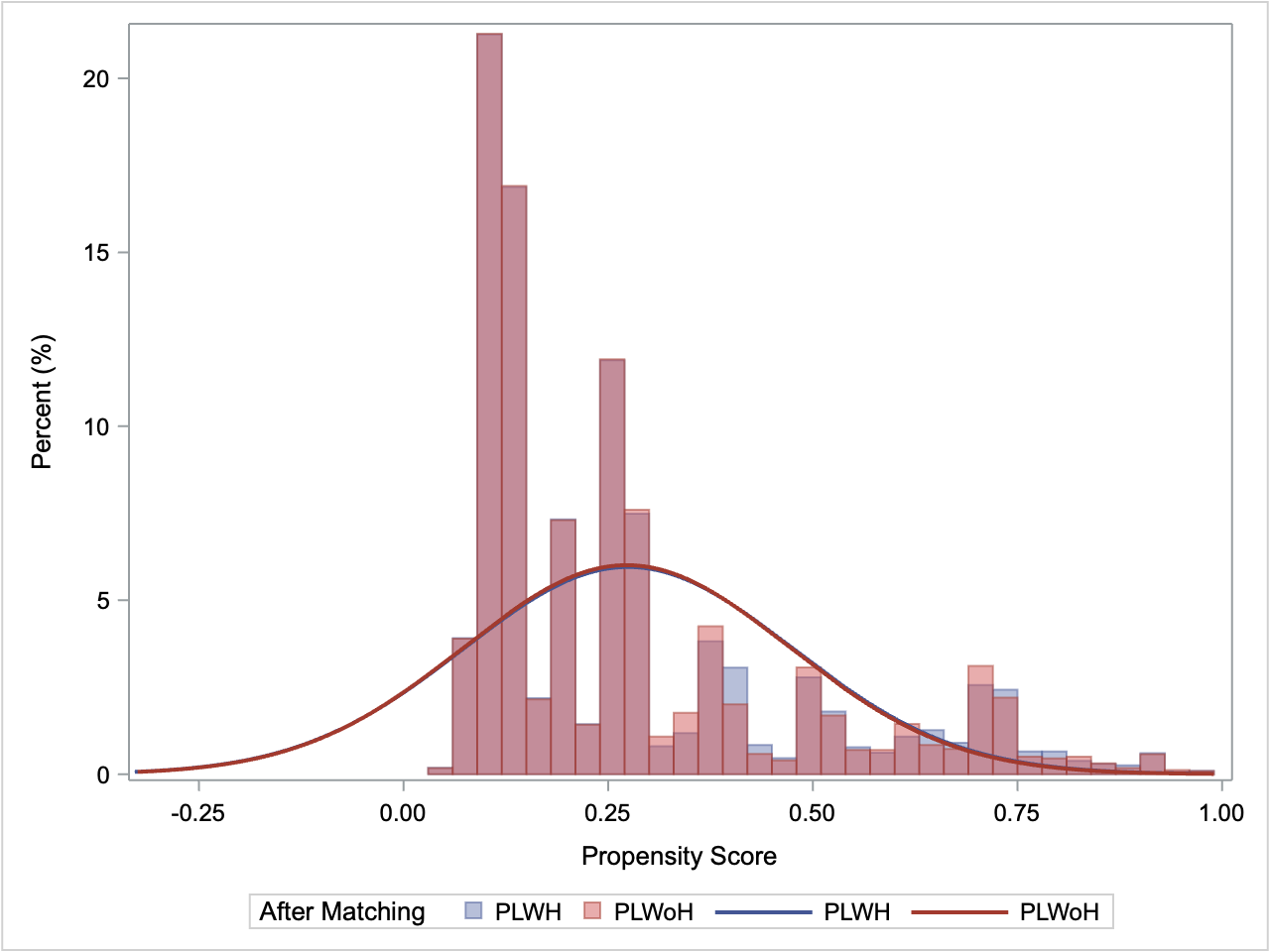


**Note:** PSM: propensity score matching; PLWH: people living with HIV; PLWoH: people living without HIV.

# **Figure S3.** Standardised mean differences before and after matching


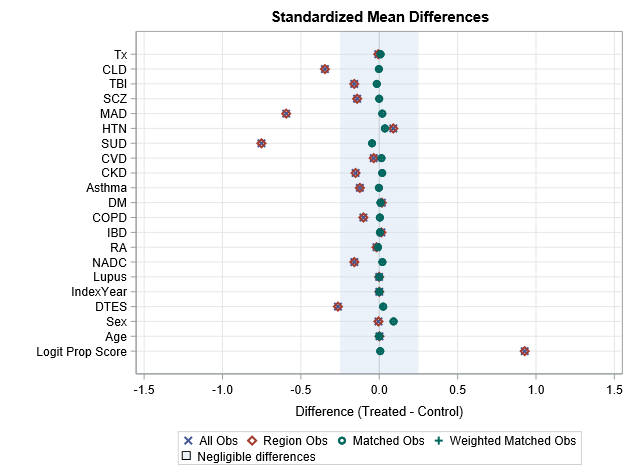


**Note.** NADC: Non–AIDS-Defining Cancer; RA: Rheumatoid Arthritis; IBD: Inflammatory Bowel Disease; COPD: Chronic Obstructive Pulmonary Disease; DM: Diabetes Mellitus; CKD: Chronic Kidney Disease; CVD: Cardiovascular Disease; SUD: Substance Use Disorder; HTN: Hypertension; MAD: Mood/Anxiety Disorder; SCZ: Schizophrenia; TBI: Traumatic Brain Injury; CLD: Chronic Liver Disease; Tx: Transplant; DTES: Downtown Eastside.

Table S4 presents the matched baseline characteristics, showing the near-elimination of group differences that were evident in the unmatched population. These results indicate that the PSM procedure successfully created a cohort of PLWH and PLWoH who were comparable with respect to measured baseline factors.

**4. Characteristics of Individuals Retained vs. Excluded After Matching**

After propensity score matching, 9,053 PLWH and 9,053 PLWoH were retained in the matched analytic cohort, while 1,620 PLWH and 414273 PLWoH were excluded due to lack of suitable matches. Among both PLWH and PLWoH, individuals who were excluded differed systematically from those who were retained. Excluded PLWH were more likely to be younger than 50 years, reside in the Downtown Eastside, and have a history of substance use disorder, chronic obstructive pulmonary disorder, mood and anxiety disorders, schizophrenia, traumatic brain injury, asthma, chronic liver disease, and chronic kidney disease (all p <0.0001). Similar patterns were observed among excluded PLWoH. Please see Table S3.

Across both study groups, exclusions were concentrated among individuals with higher levels of social vulnerability and a greater burden of comorbid conditions, reflecting limited overlap in baseline characteristics between the two groups in these subpopulations. In contrast, individuals retained in the matched cohort exhibited more comparable sociodemographic and clinical characteristics, indicating that the matching procedure successfully restricted the analytic sample to individuals for whom meaningful comparisons could be made based on observed baseline factors.

# **Table S3.** Characteristics of individuals retained versus excluded after propensity score matching, at index date.

|  | **PLWH** | |  | **PLWoH** | |  |
| --- | --- | --- | --- | --- | --- | --- |
| **Variable** | **Excluded** | **Retained** | **P-value** | **Excluded** | **Retained** | **P-value** |
|  | **n = 1620**  n(%) | **n = 9053**  n(%) |  | **n = 414273**  n(%) | **n = 9053**  n(%) |  |
| Index Age (years) |  |  |  |  |  |  |
| <50 | 1508(17) | 7311(83) | <0.0001 | 296128(98) | 7311(2) | <0.0001 |
| ≥50 | 112(6) | 1742(94) |  | 118145(99) | 1742(1) |  |
| Index Year | |  |  |  |  |  |
| <2009 | 1389(18) | 6124(82) | <0.0001 | 358080(98) | 6124(2) | <0.0001 |
| ≥2009 | 231(7) | 2929(93) |  | 56193(95) | 2929(5) |  |
| Sex |  |  |  |  |  |  |
| Male | 1157(13) | 7678(87) | <0.0001 | 204477(96) | 7990(4) | <0.0001 |
| Female | 463(25) | 1375(75) |  | 209796(99) | 1063(1) |  |
| Downtown Eastside residence | |  |  |  |  |  |
| No | 1313(13) | 8822(87) | <0.0001 | 413163(98) | 8785(2) | <0.0001 |
| Yes | 307(57) | 231(43) |  | 1110(81) | 268(19) |  |
| Systemic lupus erythematosus | | |  |  |  |  |
| No | 1616–1619(15) | 9047(85) | 0.9475 | 413960(98) | 9047(2) | 0.7503 |
| Yes | 1–4(10–14) | 6(86–90) |  | 313(98) | 6(2) |  |
| Non-AIDS defining cancer | | |  |  |  |  |
| No | 1395(15) | 7841(85) | 0.5863 | 371829(98) | 7785(2) | <0.0001 |
| Yes | 225(16) | 1212(84) |  | 42444(97) | 1268(3) |  |
| Rheumatoid arthritis | |  |  |  |  |  |
| No | 1608(15) | 9012(85) | 0.1290 | 411744(98) | 9017(2) | 0.0098 |
| Yes | 12(23) | 41(77) |  | 2529(99) | 36(1) |  |
| Inflammatory bowel disease | | |  |  |  |  |
| No | 1615(15) | 9030(85) | 0.6924 | 413297(98) | 9028(2) | 0.4319 |
| Yes | 5(18) | 23(82) |  | 976(98) | 25(2) |  |
| Chronic obstructive pulmonary disease | | | |  |  |  |
| No | 1565(15) | 8895(85) | <0.0001 | 408451(98) | 8891(2) | 0.0022 |
| Yes | 55(26) | 158(74) |  | 5822(97) | 162(3) |  |
| Diabetes mellitus | |  |  |  |  |  |
| No | 1585(15) | 8693(85) | <0.0001 | 398490(98) | 8679(2) | 0.1143 |
| Yes | 35(9) | 360(91) |  | 15783(98) | 374(2) |  |
| Asthma |  |  |  |  |  |  |
| No | 1391(14) | 8427(86) | <0.0001 | 394565(98) | 8432(2) | <0.0001 |
| Yes | 229(27) | 626(73) |  | 19708(97) | 621(3) |  |
| Chronic kidney disease | |  |  |  |  |  |
| No | 1500(14) | 8905(86) | <0.0001 | 411752(98) | 8884(2) | <0.0001 |
| Yes | 120(45) | 148(55) |  | 2521(94) | 169(6) |  |
| Cardiovascular disease | |  |  |  |  |  |
| No | 1551(15) | 8718(85) | 0.2777 | 391832(98) | 8695(2) | <0.0001 |
| Yes | 69(17) | 335(83) |  | 22441(98) | 358(2) |  |
| Substance use disorder | |  |  |  |  |  |
| No | 94(1) | 7398(99) | <0.0001 | 405699(98) | 7544(2) | <0.0001 |
| Yes | 1526(48) | 1655(52) |  | 8574(85) | 1509(15) |  |
| Hypertension |  |  |  |  |  |  |
| No | 1564(16) | 8429(84) | <0.0001 | 369268(98) | 8343(2) | <0.0001 |
| Yes | 56(8) | 624(92) |  | 45005(98) | 710(2) |  |
| Mood/anxiety disorders | |  |  |  |  |  |
| No | 382(7) | 5349(93) | <0.0001 | 336362(98) | 5270(2) | <0.0001 |
| Yes | 1238(25) | 3704(75) |  | 77911(95) | 3783(5) |  |
| Schizophrenia diagnosis | | |  |  |  |  |
| No | 1503(14) | 8863(86) | <0.0001 | 412059(98) | 8864(2) | <0.0001 |
| Yes | 117(38) | 190(62) |  | 2214(92) | 189(8) |  |
| Traumatic brain injury | |  |  |  |  |  |
| No | 1387(14) | 8660(86) | <0.0001 | 402913(98) | 8689(2) | <0.0001 |
| Yes | 233(37) | 393(63) |  | 11360(97) | 364(3) |  |
| Chronic liver disease | |  |  |  |  |  |
| No | 1126(11) | 8710(89) | <0.0001 | 412416(98) | 8715(2) | <0.0001 |
| Yes | 494(59) | 343(41) |  | 1857(85) | 338(15) |  |
| Transplant operation | |  |  |  |  |  |
| No | 1616–1619(15) | 9038(85) | 0.4751 | 413770(98) | 9035(2) | 0.0377 |
| Yes | 1–4(6–21) | 15(79–94) |  | 503(97) | 18(3) |  |

**Note.** PLWH: people living with HIV; PLWoH: people living without HIV; p-value significance is set at 0.05; row percentages are shown.

# **Table S4.** Descriptive characteristics of included participants after propensity score matching, by HIV status.

| **Variable** | **PLWH** | **PLWoH** | **P-value** |
| --- | --- | --- | --- |
|  | **n = 9053**  n(%) | **n = 9053**  n(%) |  |
| Index age (years) |  |  |  |
| <50 | 7311(50) | 7311(50) | 1.0000 |
| ≥50 | 1742(50) | 1742(50) |  |
| Index year |  |  |  |
| <2009 | 6124(50) | 6124(50) | 1.0000 |
| ≥2009 | 2929(50) | 2929(50) |  |
| Sex |  |  |  |
| Male | 7678(49) | 7990(51) | <0.0001 |
| Female | 1375(56) | 1063(44) |  |
| Downtown Eastside residence (Index date) | |  |  |
| No | 8822(50) | 8785(50) | 0.0930 |
| Yes | 231(46) | 268(54) |  |
| Downtown Eastside residence (End of follow-up) | |  |  |
| No | 8594(49) | 8855(51) | <0.0001 |
| Yes | 459(70) | 198(30) |  |
| Herpes zoster infection (End of follow-up) |  |  |  |
| No | 7798(48) | 8508(52) | <0.0001 |
| Yes | 1255(70) | 545(30) |  |
| Herpes zoster infection (End of follow-up) |  |  |  |
| No | 7798(48) | 8508(52) | <0.0001 |
| 1 time | 1087(68) | 506(32) |  |
| 2 or more times | 168(81) | 39(19) |  |
| Ever vaccinated (End of follow-up) |  |  |  |
| No | 8701(51) | 8482(49) | <0.0001 |
| Yes | 352(38) | 571(62) |  |
| Systemic lupus erythematosus (Index date) | |  |  |
| No | 9047(50) | 9047(50) | 1.0000 |
| Yes | 6(50) | 6(50) |  |
| Systemic lupus erythematosus (End of follow-up) | |  |  |
| No | 9035(50) | 9037(50) | 0.7314 |
| Yes | 18(53) | 16(47) |  |
| Non-AIDS-defining cancer (Index date) | | |  |
| No | 7841(50) | 7785(50) | 0.2261 |
| Yes | 1212(49) | 1268(51) |  |
| Non-AIDS-defining cancer (End of follow-up) | |  |  |
| No | 5951(49) | 6272(51) | <0.0001 |
| Yes | 3102(53) | 2781(47) |  |
| Rheumatoid arthritis (Index date) | |  |  |
| No | 9012(50) | 9017(50) | 0.5680 |
| Yes | 41(53) | 36(47) |  |
| Rheumatoid arthritis (End of follow-up) | |  |  |
| No | 8952(50) | 8966(50) | 0.3047 |
| Yes | 101(54) | 87(46) |  |
| Inflammatory bowel disease (Index date) | | |  |
| No | 9030(50) | 9028(50) | 0.7725 |
| Yes | 23(48) | 25(52) |  |
| Inflammatory bowel disease (End of follow-up) | |  |  |
| No | 9017(50) | 9008(50) | 0.3162 |
| Yes | 36(44) | 45(56) |  |
| Chronic obstructive pulmonary disease (Index date) | | |  |
| No | 8895(50) | 8891(50) | 0.8215 |
| Yes | 158(49) | 162(51) |  |
| Chronic obstructive pulmonary disease (End of follow-up) | |  |  |
| No | 8157(49) | 8402(51) | <0.0001 |
| Yes | 896(58) | 651(42) |  |
| Diabetes mellitus (Index date) | |  |  |
| No | 8693(50) | 8679(50) | 0.5978 |
| Yes | 360(49) | 374(51) |  |
| Diabetes mellitus (End of follow-up) | |  |  |
| No | 7763(50) | 7785(50) | 0.6388 |
| Yes | 1290(50) | 1268(50) |  |
| Asthma (Index date) | |  |  |
| No | 8427(50) | 8432(50) | 0.8833 |
| Yes | 626(50) | 621(50) |  |
| Asthma (End of follow-up) | |  |  |
| No | 7736(49) | 7928(51) | <0.0001 |
| Yes | 1317(54) | 1125(46) |  |
| Chronic kidney disease (Index date) | | |  |
| No | 8905(50) | 8884(50) | 0.2341 |
| Yes | 148(47) | 169(53) |  |
| Chronic kidney disease (End of follow-up) | |  |  |
| No | 7791(48) | 8539(52) | <0.0001 |
| Yes | 1262(71) | 514(29) |  |
| Cardiovascular disease (Index date) | | |  |
| No | 8718(50) | 8695(50) | 0.3730 |
| Yes | 335(48) | 358(52) |  |
| Cardiovascular disease (End of follow-up) | |  |  |
| No | 7644(50) | 7726(50) | 0.0889 |
| Yes | 1409(51) | 1327(49) |  |
| Substance use disorder (Index date) | | |  |
| No | 7398(50) | 7544(50) | 0.0043 |
| Yes | 1655(52) | 1509(48) |  |
| Substance use disorder (End of follow-up) | |  |  |
| No | 5762(45) | 7137(55) | <0.0001 |
| Yes | 3291(63) | 1916(37) |  |
| Hypertension (Index date) | |  |  |
| No | 8429(50) | 8343(50) | 0.0144 |
| Yes | 624(47) | 710(53) |  |
| Hypertension (End of follow-up) | |  |  |
| No | 6838(51) | 6487(49) | <0.0001 |
| Yes | 2215(46) | 2566(54) |  |
| Mood/anxiety disorders (Index date) | | |  |
| No | 5349(50) | 5270(50) | 0.2332 |
| Yes | 3704(49) | 3783(51) |  |
| Mood/anxiety disorders (End of follow-up) | |  |  |
| No | 3479(45) | 4171(55) | <0.0001 |
| Yes | 5574(53) | 4882(47) |  |
| Schizophrenia (Index date) | |  |  |
| No | 8863(50) | 8864(50) | 0.9586 |
| Yes | 190(50) | 189(50) |  |
| Schizophrenia (End of follow-up) | |  |  |
| No | 8543(50) | 8711(50) | <0.0001 |
| Yes | 510(60) | 342(40) |  |
| Traumatic brain injury (Index date) | | |  |
| No | 8660(50) | 8689(50) | 0.2816 |
| Yes | 393(52) | 364(48) |  |
| Traumatic brain injury (End of follow-up) | |  |  |
| No | 7856(50) | 8013(50) | 0.0004 |
| Yes | 1197(54) | 1040(46) |  |
| Chronic liver disease (Index date) | |  |  |
| No | 8710(50) | 8715(50) | 0.8452 |
| Yes | 343(50) | 338(50) |  |
| Chronic liver disease (End of follow-up) | |  |  |
| No | 7077(46) | 8474(54) | <0.0001 |
| Yes | 1976(77) | 579(23) |  |
| Transplant operation (Index date) | |  |  |
| No | 9038(50) | 9035(50) | 0.6012 |
| Yes | 15(45) | 18(55) |  |
| Transplant operation (End of follow-up) | |  |  |
| No | 8963(50) | 8951(50) | 0.3839 |
| Yes | 90(47) | 102(53) |  |
| **Continuous Variables** | | | |
| Age (years)(Index date) |  |  |  |
| Median | 39 | 39 | 0.8000 |
| 25^th^–75^th^ percentiles | 33–46 | 33–46 |  |
| Age (years)(End of follow-up) |  |  |  |
| Median | 52 | 54 | <0.0001 |
| 25^th^­–75^th^ percentiles | 44–59 | 45–60 |  |
| Follow-up time (person-years) |  |  |  |
| Total | 106356 | 118610 | . |
| Median (25^th^–75^th^ percentiles) | 12(6–18) | 14(7–20) | <0.0001 |

**Note.**  PLWH: People living with HIV/AIDS; PLWoH: People living without HIV/AIDS; p-value significance is set at 0.05; row percentages are shown.

# **Table S5.** Bivariable comparison of study variables by herpes zoster status among people living with HIV.

| **Variable** | **Never Had**  **Herpes Zoster** | **Ever Had**  **Herpes Zoster** | **P-value** |
| --- | --- | --- | --- |
|  | **n = 7798**  n(%) | **n = 1255**  n(%) |  |
| Sex |  |  |  |
| Male | 6629(86) | 1049(14) | 0.1923 |
| Female | 1169(85) | 206(15) |  |
| Downtown Eastside residence (Index date) | |  |  |
| No | 7593(86) | 1229(14) | 0.2453 |
| Yes | 205(89) | 26(11) |  |
| Downtown Eastside residence (End of follow-up) |  |  |  |
| No | 7379(86) | 1215(14) | 0.0011 |
| Yes | 419(91) | 40(9) |  |
| CD4 (cells/mm^3^)(Index date) | |  |  |
| <50 | 483(87) | 73(13) | 0.0890 |
| 50─199 | 1077(84) | 198(16) |  |
| 200─349 | 1457(85) | 251(15) |  |
| ≥350 | 2930(87) | 427(13) |  |
| Not measured | 1851(86) | 306(14) |  |
| CD4 (cells/mm^3^)(End of follow-up) | |  |  |
| <50 | 312(86) | 49(14) | 0.0420 |
| 50─199 | 627(84) | 118(16) |  |
| 200─349 | 921(85) | 160(15) |  |
| ≥350 | 4227(86) | 697(14) |  |
| Not measured | 1711(88) | 231(12) |  |
| Uncontrolled viremia (Index date) | |  |  |
| Not suppressed | 4849(84) | 890(16) | <0.0001 |
| Suppressed | 1900(90) | 216(10) |  |
| Not measured | 1049(88) | 149(12) |  |
| Uncontrolled viremia (End of follow-up) | |  |  |
| Not suppressed | 1120(87) | 174(13) | <0.0001 |
| Suppressed | 5659(85) | 966(15) |  |
| Not measured | 1019(90) | 115(10) |  |
| Year of antiretroviral therapy initiation | |  |  |
| <2000 | 2355(83) | 481(17) | <0.0001 |
| 2000─2009 | 2711(84) | 512(16) |  |
| 2010─2019 | 2732(91) | 262(9) |  |
| Herpes zoster infection (End of follow-up) |  |  |  |
| No | 7798(100) | 0(0) | <0.0001 |
| 1 time | 0(0) | 1087(100) |  |
| 2 or more times | 0(0) | 168(100) |  |
| Ever vaccinated (End of follow-up) |  |  |  |
| No | 7515(86) | 1186(14) | 0.0015 |
| Yes | 283(80) | 69(20) |  |
| Systemic lupus erythematosus (Index date) | |  |  |
| No | 7794–7797(86) | 1251-1254(14) | 0.1674 |
| Yes | <5(20–80) | <5(20–80) |  |
| Systemic lupus erythematosus (End of follow-up) | |  |  |
| No | 7783(86) | 1251–1254(14) | 0.7304 |
| Yes | 15(79–94) | <5(6–11) |  |
| Non-AIDS-defining cancer (Index date) | | |  |
| No | 6777(86) | 1064(14) | 0.0401 |
| Yes | 1021(84) | 191(16) |  |
| Non-AIDS-defining cancer (End of follow-up) | |  |  |
| No | 5263(88) | 688(12) | <0.0001 |
| Yes | 2535(82) | 567(18) |  |
| Rheumatoid arthritis (Index date) | |  |  |
| No | 7767(86) | 1245(14) | 0.0506 |
| Yes | 31(76) | 10(24) |  |
| Rheumatoid arthritis (End of follow-up) |  |  |  |
| No | 7722(86) | 1230(14) | 0.0014 |
| Yes | 76(75) | 25(25) |  |
| Inflammatory bowel disease (Index date) | | |  |
| No | 7782(86) | 1248(14) | 0.0213 |
| Yes | 16(70) | 7(30) |  |
| Inflammatory bowel disease (End of follow-up) | |  |  |
| No | 7770(86) | 1247(14) | 0.1458 |
| Yes | 28(78) | 8(22) |  |
| Chronic obstructive pulmonary disease (Index date) | | |  |
| No | 7659(86) | 1236(14) | 0.5001 |
| Yes | 139(88) | 19(12) |  |
| Chronic obstructive pulmonary disease (End of follow-up) | |  |  |
| No | 7052(86) | 1105(14) | 0.0086 |
| Yes | 746(83) | 150(17) |  |
| Diabetes mellitus (Index date) | |  |  |
| No | 7482(86) | 1211(14) | 0.3580 |
| Yes | 316(88) | 44(12) |  |
| Diabetes mellitus (End of follow-up) | |  |  |
| No | 6735(87) | 1028(13) | <0.0001 |
| Yes | 1063(82) | 227(18) |  |
| Asthma (Index date) | |  |  |
| No | 7281(86) | 1146(14) | 0.0077 |
| Yes | 517(83) | 109(17) |  |
| Asthma (End of follow-up) | |  |  |
| No | 6734(87) | 1002(13) | <0.0001 |
| Yes | 1064(81) | 253(19) |  |
| Chronic kidney disease (Index date) | | |  |
| No | 7662(86) | 1243(14) | 0.0411 |
| Yes | 136(92) | 12(8) |  |
| Chronic kidney disease (End of follow-up) | |  |  |
| No | 6775(87) | 1016(13) | <0.0001 |
| Yes | 1023(81) | 239(19) |  |
| Cardiovascular disease (Index date) | |  |  |
| No | 7514(86) | 1204(14) | 0.4626 |
| Yes | 284(85) | 51(15) |  |
| Cardiovascular disease (End of follow-up) | |  |  |
| No | 6661(87) | 983(13) | <0.0001 |
| Yes | 1137(81) | 272(19) |  |
| Substance use disorder (Index date) | | |  |
| No | 6346(86) | 1052(14) | 0.0376 |
| Yes | 1452(88) | 203(12) |  |
| Substance use disorder (End of follow-up) | |  |  |
| No | 5012(87) | 750(13) | 0.0020 |
| Yes | 2786(85) | 505(15) |  |
| Hypertension (Index date) | |  |  |
| No | 7265(86) | 1164(14) | 0.5893 |
| Yes | 533(85) | 91(15) |  |
| Hypertension (End of follow-up) | |  |  |
| No | 5998(88) | 840(12) | <0.0001 |
| Yes | 1800(81) | 415(19) |  |
| Mood/anxiety disorders (Index date) | | |  |
| No | 4695(88) | 654(12) | <0.0001 |
| Yes | 3103(84) | 601(16) |  |
| Mood/anxiety disorders (End of follow-up) | |  |  |
| No | 3133(90) | 346(10) | <0.0001 |
| Yes | 4665(84) | 909(16) |  |
| Schizophrenia (Index date) | |  |  |
| No | 7626(86) | 1237(14) | 0.0768 |
| Yes | 172(91) | 18(9) |  |
| Schizophrenia (End of follow-up) | |  |  |
| No | 7360(86) | 1183(14) | 0.8639 |
| Yes | 438(86) | 72(14) |  |
| Traumatic brain injury (Index date) | |  |  |
| No | 7453(86) | 1207(14) | 0.3334 |
| Yes | 345(88) | 48(12) |  |
| Traumatic brain injury (End of follow-up) | |  |  |
| No | 6789(86) | 1067(14) | 0.0476 |
| Yes | 1009(84) | 188(16) |  |
| Chronic liver disease (Index date) | |  |  |
| No | 7494(86) | 1216(14) | 0.1732 |
| Yes | 304(89) | 39(11) |  |
| Chronic liver disease (End of follow-up) |  |  |  |
| No | 6168(87) | 909(13) | <0.0001 |
| Yes | 1630(82) | 346(18) |  |
| Transplant operation (Index date) | |  |  |
| No | 7784(86) | 1251–1254(14) | 0.4195 |
| Yes | 14(78–93) | <5(7–22) |  |
| Transplant operation (End of follow-up) | |  |  |
| No | 7728(86) | 1235(14) | 0.0211 |
| Yes | 70(78) | 20(22) |  |
| **Continuous Variables** | | | |
| Age (years)(Index date) |  |  |  |
| Median | 40 | 39 | 0.8237 |
| 25^th^–75^th^ percentiles | 33─47 | 33─47 |  |
| Age (years)(End of follow-up) |  |  |  |
| Median | 52 | 55 | <0.0001 |
| 25^th^­–75^th^ percentiles | 43─59 | 48─62 |  |
| Follow-up time (person-years) |  |  |  |
| Total | 87874 | 18482 | . |
| Median (25^th^–75^th^ percentiles) | 11(6–18) | 17(10–20) | <0.0001 |

**Note.** P-value significance is set at 0.05; row percentages are shown.

# **Table S6.** Bivariable comparison of study variables by herpes zoster status among people living without HIV.

| **Variable** | **Never Had**  **Herpes Zoster** | **Ever Had**  **Herpes Zoster** | **P-value** |
| --- | --- | --- | --- |
|  | **n = 8508**  n(%) | **n = 545**  n(%) |  |
| Sex |  |  |  |
| Male | 7528(94) | 462(6) | 0.0091 |
| Female | 980(92) | 83(8) |  |
| Downtown Eastside residence (Index date) | |  |  |
| No | 8258(94) | 527(6) | 0.6266 |
| Yes | 250(93) | 18(7) |  |
| Downtown Eastside residence (End of follow-up) | |  |  |
| No | 8318(94) | 537(6) | 0.2363 |
| Yes | 190(96) | 8(4) |  |
| Herpes zoster infection (End of follow-up) |  |  |  |
| No | 8508(100) | 0(0) | <0.0001 |
| 1 time | 0(0) | 506(100) |  |
| 2 or more times | 0(0) | 39(100) |  |
| Ever vaccinated (End of follow-up) |  |  |  |
| No | 8004(94) | 478(6) | <0.0001 |
| Yes | 504(88) | 67(12) |  |
| Systemic lupus erythematosus (Index date) | |  |  |
| No | 8502(94) | 545(6) | 0.5352 |
| Yes | 6(100) | 0(0) |  |
| Systemic lupus erythematosus (End of follow-up) | |  |  |
| No | 8493(94) | 541–544(6) | 0.9691 |
| Yes | 15(79–94) | <5(6–21) |  |
| Non-AIDS-defining cancer (Index date) | |  |  |
| No | 7349(94) | 436(6) | <0.0001 |
| Yes | 1159(91) | 109(9) |  |
| Non-AIDS-defining cancer (End of follow-up) | |  |  |
| No | 5980(95) | 292(5) | <0.0001 |
| Yes | 2528(91) | 253(9) |  |
| Rheumatoid arthritis (Index date) | |  |  |
| No | 8475(94) | 541–544(6) | 0.5588 |
| Yes | 33(89–97) | <5(3–11) |  |
| Rheumatoid arthritis (End of follow-up) | |  |  |
| No | 8431(94) | 535(6) | 0.0310 |
| Yes | 77(89) | 10(11) |  |
| Inflammatory bowel disease (Index date) | | |  |
| No | 8485(94) | 541–544(6) | 0.6768 |
| Yes | 23(85–96) | <5(4–15) |  |
| Inflammatory bowel disease (End of follow-up) | |  |  |
| No | 8467(94) | 541–544(6) | 0.4173 |
| Yes | 41(91–98) | <5(2–9) |  |
| Chronic obstructive pulmonary disease (Index date) | | |  |
| No | 8359(94) | 532(6) | 0.2791 |
| Yes | 149(92) | 13(8) |  |
| Chronic obstructive pulmonary disease (End of follow-up) | | |  |
| No | 7907(94) | 495(6) | 0.0645 |
| Yes | 601(92) | 50(8) |  |
| Diabetes mellitus (Index date) | |  |  |
| No | 8169(94) | 510(6) | 0.0056 |
| Yes | 339(91) | 35(9) |  |
| Diabetes mellitus (End of follow-up) | |  |  |
| No | 7331(94) | 454(6) | 0.0619 |
| Yes | 1177(93) | 91(7) |  |
| Asthma (Index date) | |  |  |
| No | 7923(94) | 509(6) | 0.8087 |
| Yes | 585(94) | 36(6) |  |
| Asthma (End of follow-up) | |  |  |
| No | 7469(94) | 459(6) | 0.0144 |
| Yes | 1039(92) | 86(8) |  |
| Chronic kidney disease (Index date) | |  |  |
| No | 8353(94) | 531(6) | 0.2116 |
| Yes | 155(92) | 14(8) |  |
| Chronic kidney disease (End of follow-up) | |  |  |
| No | 8040(94) | 499(6) | 0.0040 |
| Yes | 468(91) | 46(9) |  |
| Cardiovascular disease (Index date) | |  |  |
| No | 8172(94) | 523(6) | 0.9191 |
| Yes | 336(94) | 22(6) |  |
| Cardiovascular disease (End of follow-up) | |  |  |
| No | 7294(94) | 432(6) | <0.0001 |
| Yes | 1214(91) | 113(9) |  |
| Substance use disorder (Index date) | |  |  |
| No | 7078(94) | 466(6) | 0.1603 |
| Yes | 1430(95) | 79(5) |  |
| Substance use disorder (End of follow-up) | |  |  |
| No | 6697(94) | 440(6) | 0.2631 |
| Yes | 1811(95) | 105(5) |  |
| Hypertension (Index date) | |  |  |
| No | 7845(94) | 498(6) | 0.4841 |
| Yes | 663(93) | 47(7) |  |
| Hypertension (End of follow-up) | |  |  |
| No | 6149(95) | 338(5) | <0.0001 |
| Yes | 2359(92) | 207(8) |  |
| Mood/anxiety disorders (Index date) | |  |  |
| No | 4977(94) | 293(6) | 0.0298 |
| Yes | 3531(93) | 252(7) |  |
| Mood/anxiety disorders (End of follow-up) | |  |  |
| No | 3956(95) | 215(5) | 0.0014 |
| Yes | 4552(93) | 330(7) |  |
| Schizophrenia (Index date) | |  |  |
| No | 8327(94) | 537(6) | 0.2965 |
| Yes | 181(96) | 8(4) |  |
| Schizophrenia (End of follow-up) | |  |  |
| No | 8188(94) | 523(6) | 0.7436 |
| Yes | 320(94) | 22(6) |  |
| Traumatic brain injury (Index date) | |  |  |
| No | 8170(94) | 519(6) | 0.3580 |
| Yes | 338(93) | 26(7) |  |
| Traumatic brain injury (End of follow-up) | |  |  |
| No | 7528(94) | 485(6) | 0.7177 |
| Yes | 980(94) | 60(6) |  |
| Chronic liver disease (Index date) | |  |  |
| No | 8187(94) | 528(6) | 0.4352 |
| Yes | 321(95) | 17(5) |  |
| Chronic liver disease (End of follow-up) | |  |  |
| No | 7962(94) | 512(6) | 0.7374 |
| Yes | 546(94) | 33(6) |  |
| Transplant operation (Index date) | |  |  |
| No | 8493(94) | 541–544(6) | 0.0573 |
| Yes | 15(79–94) | <5(6–21) |  |
| Transplant operation (End of follow-up) | |  |  |
| No | 8416(94) | 535(6) | 0.1062 |
| Yes | 92(90) | 10(10) |  |
| **Continuous Variables** | | | |
| Age (years)(Index date) |  |  |  |
| Median | 40 | 43 | <0.0001 |
| 25^th^–75^th^ percentiles | 34–47 | 36–51 |  |
| Age (years)(End of follow-up) |  |  |  |
| Median | 54 | 58 | <0.0001 |
| 25^th^­–75^th^ percentiles | 46–60 | 52–66 |  |
| Follow-up time (person-years) |  |  |  |
| Total | 109871 | 8739 | . |
| Median (25^th^–75^th^ percentiles) | 14(7–20) | 18(13–20) | <0.0001 |

**Note.** P-value significance is set at 0.05; row percentages are shown.

# **Table S7.** Bivariable comparison of study variables by the number of episodes of herpes zoster (HZ) infection among people living with HIV.

| **Variable** | **Had Herpes**  **Zoster Once** | **Had Herpes Zoster**  **More Than Once** | **P-value** |
| --- | --- | --- | --- |
|  | **n = 1087**  n(%) | **n = 168**  n(%) |  |
| Sex |  |  |  |
| Male | 915(87) | 134(13) | 0.1505 |
| Female | 172(83) | 34(17) |  |
| Downtown Eastside residence (Index date) | |  |  |
| No | 1064(86–87) | 164–167(13–14) | 0.7797 |
| Yes | 23(85–96) | <5(4–15) |  |
| Downtown Eastside residence (End of follow-up) | |  |  |
| No | 1064(86–87) | 164–167(13–14) | 0.5230 |
| Yes | 36(90–97) | <5(3–10) |  |
| CD4 (cells/mm^3^)(Index date) | |  |  |
| <50 | 67(92) | 6(8) | 0.6246 |
| 50–199 | 174(88) | 24(12) |  |
| 200–349 | 215(86) | 36(14) |  |
| ≥350 | 370(87) | 57(13) |  |
| Not measured | 261(85) | 45(15) |  |
| CD4 (cells/mm^3^)(End of follow-up) | |  |  |
| <50 | 45(92–98) | <5(2–8) | 0.2910 |
| 50–199 | 96(79–81) | 22–25(19–21) |  |
| 200–349 | 136(85) | 24(15) |  |
| ≥350 | 606(87) | 91(13) |  |
| Not measured | 204(88) | 27(12) |  |
| Uncontrolled viremia (Index date) | |  |  |
| Not suppressed | 769(86) | 121(14) | 0.1807 |
| Suppressed | 194(90) | 22(10) |  |
| Not measured | 124(83) | 25(17) |  |
| Uncontrolled viremia (End of follow-up) | |  |  |
| Not suppressed | 148(85) | 26(15) | 0.6720 |
| Suppressed | 837(87) | 129(13) |  |
| Not measured | 102(89) | 13(11) |  |
| Year of antiretroviral therapy initiation | |  |  |
| <2000 | 408(85) | 73(15) | 0.0650 |
| 2000–2009 | 441(86) | 71(14) |  |
| 2010–2019 | 238(91) | 24(9) |  |
| Herpes zoster infection (End of follow-up) |  |  |  |
| 1 time | 1087(100) | 0(0) | <0.0001 |
| 2 or more times | 0(0) | 168(100) |  |
| Ever vaccinated (End of follow-up) |  |  |  |
| No | 1036(87) | 150(13) | 0.0014 |
| Yes | 51(74) | 18(26) |  |
| Systemic lupus erythematosus (Index date) | |  |  |
| No | 1083–1086(87) | 164–167(13) | 0.1280 |
| Yes | <5(20–80) | <5(20–80) |  |
| Systemic lupus erythematosus (End of follow-up) | |  |  |
| No | 1083–1086(87) | 164–167(13) | 0.0070 |
| Yes | <5(20–80) | <5(20–80) |  |
| Non-AIDS-defining cancer (Index date) | |  |  |
| No | 923(87) | 141(13) | 0.7411 |
| Yes | 164(86) | 27(14) |  |
| Non-AIDS-defining cancer (End of follow-up) | |  |  |
| No | 611(89) | 77(11) | 0.0120 |
| Yes | 476(84) | 91(16) |  |
| Rheumatoid arthritis (Index date) | |  |  |
| No | 1077(87) | 168(13) | 0.2120 |
| Yes | 10(100) | 0(0) |  |
| Rheumatoid arthritis (End of follow-up) | |  |  |
| No | 1066(86–87) | 164–167(13–14) | 0.6980 |
| Yes | 21(84–95) | <5(5–16) |  |
| Inflammatory bowel disease (Index date) | |  |  |
| No | 1081(87) | 164–167(13) | 0.9441 |
| Yes | 6(60–86) | <5(14–40) |  |
| Inflammatory bowel disease End of follow-up) | |  |  |
| No | 1080(87) | 164–167(13) | 0.9410 |
| Yes | 7(64–88) | <5(13–36) |  |
| Chronic obstructive pulmonary disease (Index date) | | |  |
| No | 1071(87) | 164–167(13) | 0.7566 |
| Yes | 16(80–94) | <5(16–20) |  |
| Chronic obstructive pulmonary disease (End of follow-up) | |  |  |
| No | 964(87) | 141(13) | 0.0770 |
| Yes | 123(82) | 27(18) |  |
| Diabetes mellitus (Index date) | |  |  |
| No | 1049(87) | 162(13) | 0.9605 |
| Yes | 38(86) | 6(14) |  |
| Diabetes mellitus (End of follow-up) | |  |  |
| No | 895(87) | 133(13) | 0.3200 |
| Yes | 192(85) | 35(15) |  |
| Asthma (Index date) | |  |  |
| No | 995(87) | 151(13) | 0.4783 |
| Yes | 92(84) | 17(16) |  |
| Asthma (End of follow-up) | |  |  |
| No | 878(88) | 124(12) | 0.0360 |
| Yes | 209(83) | 44(17) |  |
| Chronic kidney disease (Index date) | |  |  |
| No | 1077(87) | 164–167(13) | 0.7374 |
| Yes | 10(71–91) | <5(8–29) |  |
| Chronic kidney disease (End of follow-up) | |  |  |
| No | 884(87) | 132(13) | 0.3980 |
| Yes | 203(85) | 36(15) |  |
| Cardiovascular disease (Index date) | |  |  |
| No | 1044(87) | 160(13) | 0.6224 |
| Yes | 43(84) | 8(16) |  |
| Cardiovascular disease (End of follow-up) | |  |  |
| No | 854(87) | 129(13) | 0.6020 |
| Yes | 233(86) | 39(14) |  |
| Substance use disorder (Index date) | |  |  |
| No | 913(87) | 139(13) | 0.6811 |
| Yes | 174(86) | 29(14) |  |
| Substance use disorder (End of follow-up) | |  |  |
| No | 652(87) | 98(13) | 0.6850 |
| Yes | 435(86) | 70(14) |  |
| Hypertension (Index date) | |  |  |
| No | 1014(87) | 150(13) | 0.0629 |
| Yes | 73(80) | 18(20) |  |
| Hypertension (End of follow-up) | |  |  |
| No | 732(87) | 108(13) | 0.4330 |
| Yes | 355(86) | 60(14) |  |
| Mood/anxiety disorders (Index date) | |  |  |
| No | 586(90) | 68(10) | 0.0012 |
| Yes | 501(83) | 100(17) |  |
| Mood/anxiety disorders (End of follow-up) | |  |  |
| No | 318(92) | 28(8) | 0.0010 |
| Yes | 769(85) | 140(15) |  |
| Schizophrenia (Index date) | |  |  |
| No | 1071(87) | 164–167(13) | 0.7752 |
| Yes | 16(80–94) | <5(6–20) |  |
| Schizophrenia (End of follow-up) | |  |  |
| No | 1026(87) | 157(13) | 0.6270 |
| Yes | 61(85) | 11(15) |  |
| Traumatic brain injury (Index date) | |  |  |
| No | 1044(86) | 163(14) | 0.5378 |
| Yes | 43(90) | 5(10) |  |
| Traumatic brain injury (End of follow-up) | |  |  |
| No | 925(87) | 142(13) | 0.8460 |
| Yes | 162(86) | 26(14) |  |
| Chronic liver disease (Index date) | |  |  |
| No | 1053(87) | 163(13) | 0.9160 |
| Yes | 34(87) | 5(13) |  |
| Chronic liver disease (End of follow-up) | |  |  |
| No | 796(88) | 113(12) | 0.1070 |
| Yes | 291(84) | 55(16) |  |
| Transplant operation (Index date) | |  |  |
| No | 1063–1086 (86–87) | 168(13–14) | 0.6941 |
| Yes | <5(100) | 0(0) |  |
| Transplant operation (End of follow-up) | |  |  |
| No | 1069(86–87) | 164–167(13–14) | 0.6540 |
| Yes | 18(82–95) | <5(10) |  |
| **Continuous Variables** | | | |
| Age (years)(Index date) |  |  |  |
| Median | 39 | 39 | 0.2599 |
| 25^th^–75^th^ percentiles | 33–48 | 33–45 |  |
| Age (years)(End of follow-up) |  |  |  |
| Median | 54 | 55 | 0.0921 |
| 25^th^­–75^th^ percentiles | 48–62 | 50–63 |  |
| Follow-up time (person-years) |  |  |  |
| Total | 15621 | 2860 |  |
| Median (25^th^–75^th^ percentiles) | 16(10–20) | 19(15–20) | <0.0001 |

**Note.** NA: Not applicable; p-value significance is set at 0.05; row percentages are shown.

# **Table S8.** Bivariable comparison of study variables by the number of episodes of herpes zoster (HZ) infection among people living without HIV.

| **Variable** | **Had Herpes**  **Zoster Once** | | **Had Herpes Zoster**  **More Than Once** | **P-value** |
| --- | --- | --- | --- | --- |
|  | **n = 506**  n(%) | | **n = 39**  n(%) |  |
| Sex |  | |  |  |
| Male | 432(94) | | 30(6) | 0.1569 |
| Female | 74(89) | | 9(11) |  |
| Downtown Eastside residence (Index date) | | |  |  |
| No | | 488(93) | 39(7) | 0.2310 |
| Yes | | 18(100) | 0(0) |  |
| Downtown Eastside residence (End of follow-up) | | |  |  |
| No | | 498(93) | 39(7) | 0.4289 |
| Yes | | 8(100) | 0(0) |  |
| Herpes zoster infection (End of follow-up) | |  |  |  |
| 1 time | | 506(100) | 0(0) | <0.0001 |
| 2 or more times | | 0(0) | 39(100) |  |
| Ever vaccinated (End of follow-up) | |  |  |  |
| No | | 445(93) | 33(7) | 0.5418 |
| Yes | | 61(91) | 6(9) |  |
| Systemic lupus erythematosus (Index date) | |  |  |  |
| No | | 506(93) | 39(7) | NA |
| Yes | | 0(0) | 0(0) |  |
| Systemic lupus erythematosus (End of follow-up) | |  |  |  |
| No | | 502–505(93) | 39(7) | 0.7811 |
| Yes | | <5(100) | 0(0) |  |
| Non-AIDS-defining cancer (Index date) | | |  |  |
| No | | 404(93) | 32(7) | 0.7396 |
| Yes | | 102(94) | 7(6) |  |
| Non-AIDS-defining cancer (End of follow-up) | | |  |  |
| No | | 271(93) | 21(7) | 0.9722 |
| Yes | | 235(93) | 18(7) |  |
| Rheumatoid arthritis (Index date) | | |  |  |
| No | | 502–505(93) | 39(7) | 0.6297 |
| Yes | | <5(100) | 0(0) |  |
| Rheumatoid arthritis (End of follow-up) | | |  |  |
| No | | 497(93) | 35–38(7) | 0.7247 |
| Yes | | 9(69–90) | <5(10–31) |  |
| Inflammatory bowel disease (Index date) | | |  |  |
| No | | 502–505(93) | 39(7) | 0.6941 |
| Yes | | <5(100) | 0(0) |  |
| Inflammatory bowel disease (End of follow-up) | | |  |  |
| No | | 502–505(93–94) | 35–38(6–7) | 0.1646 |
| Yes | | <5(20–80) | <5(20–80) |  |
| Chronic obstructive pulmonary disease (Index date) | | |  |  |
| No | | 494(93) | 35–38(7) | 0.9395 |
| Yes | | 12(75–92) | <5(8–25) |  |
| Chronic obstructive pulmonary disease (End of follow-up) | | |  |  |
| No | | 459(92–93) | 35–38(7–8) | 0.7393 |
| Yes | | 47(92–98) | <5(2–8) |  |
| Diabetes mellitus (Index date) | |  |  |  |
| No | | 472(93) | 35–38(7) | 0.3077 |
| Yes | | 34(89–97) | <5(3–11) |  |
| Diabetes mellitus (End of follow-up) | |  |  |  |
| No | | 419(92) | 35–38(8) | 0.2630 |
| Yes | | 87(96–99) | <5(1–4) |  |
| Asthma (Index date) | |  |  |  |
| No | | 475(93) | 34(7) | 0.1049 |
| Yes | | 31(86) | 5(14) |  |
| Asthma (End of follow-up) | |  |  |  |
| No | | 431(94) | 28(6) | 0.0272 |
| Yes | | 75(87) | 11(13) |  |
| Chronic kidney disease (Index date) | | |  |  |
| No | | 493(93) | 35–38(7) | 0.9985 |
| Yes | | 13(76–93) | <5(7–24) |  |
| Chronic kidney disease (End of follow-up) | | |  |  |
| No | | 463(93) | 35–38(7) | 0.8615 |
| Yes | | 43(91–98) | <5(2–9) |  |
| Cardiovascular disease (Index date) | | |  |  |
| No | | 485(92–93) | 35–38(7–8) | 0.6277 |
| Yes | | 21(84–95) | <5(5–16) |  |
| Cardiovascular disease (End of follow-up) | | |  |  |
| No | | 399(92) | 33(8) | 0.3924 |
| Yes | | 107(95) | 6(5) |  |
| Substance use disorder (Index date) | | |  |  |
| No | | 433(93) | 33(7) | 0.8700 |
| Yes | | 73(92) | 6(8) |  |
| Substance use disorder (End of follow-up) | | |  |  |
| No | | 411(93) | 29(7) | 0.2948 |
| Yes | | 95(90) | 10(10) |  |
| Hypertension (Index date) | |  |  |  |
| No | | 461(92–93) | 35–38(7–8) | 0.4196 |
| Yes | | 45(92–98) | <5(2–8) |  |
| Hypertension (End of follow-up) | |  |  |  |
| No | | 313(93) | 25(7) | 0.7808 |
| Yes | | 193(93) | 14(7) |  |
| Mood/anxiety disorders (Index date) | | |  |  |
| No | | 276(94) | 17(6) | 0.1861 |
| Yes | | 230(91) | 22(9) |  |
| Mood/anxiety disorders (End of follow-up) | | |  |  |
| No | | 204(95) | 11(5) | 0.1359 |
| Yes | | 302(92) | 28(8) |  |
| Schizophrenia (Index date) | |  |  |  |
| No | | 498(93) | 39(7) | 0.4289 |
| Yes | | 8(100) | 0(0) |  |
| Schizophrenia (End of follow-up) | | |  |  |
| No | | 488(93) | 35–38(7) | 0.0405 |
| Yes | | 18(82–95) | <5(5–18) |  |
| Traumatic brain injury (Index date) | | |  |  |
| No | | 484(93) | 35–38(7) | 0.0953 |
| Yes | | 22(85–96) | <5(4–15) |  |
| Traumatic brain injury (End of follow-up) | | |  |  |
| No | | 454(94) | 31(6) | 0.0491 |
| Yes | | 52(87) | 8(13) |  |
| Chronic liver disease (Index date) | | |  |  |
| No | | 491(93) | 35–38(7) | 0.4539 |
| Yes | | 15(79–94) | <5(6–21) |  |
| Chronic liver disease (End of follow-up) | | |  |  |
| No | | 477(93) | 35–38(7) | 0.2536 |
| Yes | | 29(88–97) | <5(3–12) |  |
| Transplant operation (Index date) | |  |  |  |
| No | | 502–505(93) | 39(7) | 0.6297 |
| Yes | | <5(100) | 0(0) |  |
| Transplant operation (End of follow-up) | |  |  |  |
| No | | 498(93) | 35–38(7) | 0.1117 |
| Yes | | 8(67–89) | <5(11–33) |  |
| **Continuous Variables** | | | | |
| Age (years)(Index date) | |  |  |  |
| Median | | 43 | 41 | 0.3098 |
| 25^th^–75^th^ percentiles | | 36–51 | 35–47 |  |
| Age (years)(End of follow-up) | |  |  |  |
| Median | | 58 | 60 | 0.9730 |
| 25^th^­–75^th^ percentiles | | 52–67 | 53–63 |  |
| Follow-up time (person-years) | |  |  |  |
| Total | | 8047 | 692 | . |
| Median (25^th^–75^th^ percentiles) | | 18(12–20) | 20(17–20) | 0.0277 |

**Note.** NA: Not applicable; p-value significance is set at 0.05; row percentages are shown.

# **Table S9.** Bivariable comparison of study variables by herpes zoster (HZ) vaccination status among people living with HIV aged 50 years and over.

| **Variable** | **Never Had HZ Vaccination** | **Completed HZ Vaccination** | **P-value** |
| --- | --- | --- | --- |
|  | **n = 4896**  n(%) | **n = 346**  n(%) |  |
| Sex |  |  |  |
| Male | 4335(93) | 321(7) | 0.0157 |
| Female | 561(96) | 25(4) |  |
| Downtown Eastside residence (End of follow-up) | |  |  |
| No | 4657(93) | 340(7) | 0.0073 |
| Yes | 239(98) | 6(2) |  |
| CD4 (cells/mm^3^)(End of follow-up) | |  |  |
| <50 | 125(97–99) | <5(1–3) | <0.0001 |
| 50–199 | 437(99) | 5(1) |  |
| 200–349 | 695(95–96) | 31–34(4–5) |  |
| ≥350 | 2659(91) | 258(9) |  |
| Not measured | 980(95) | 51(5) |  |
| Uncontrolled viremia (End of follow-up) | |  |  |
| Not suppressed | 541(98) | 9(2) | <0.0001 |
| Suppressed | 3847(92) | 330(8) |  |
| Not measured | 508(99) | 7(1) |  |
| Herpes zoster infection (End of follow-up) |  |  |  |
| No | 4102(94) | 277(6) | 0.0710 |
| Yes | 794(92) | 69(8) |  |
| Herpes zoster infection (End of follow up) |  |  |  |
| No | 4102(94) | 277(6) | 0.0039 |
| 1 time | 686(93) | 50(7) |  |
| 2 or more times | 108(85) | 19(15) |  |
| HZ vaccine type (End of follow-up) |  |  |  |
| Live Zoster Vaccine (LZV) | NA | 202(100) | NA |
| Recombinant Zoster Vaccine (RZV) | NA | 162(100) |  |
| Both vaccines | NA | 18(100) |  |
| Year of antiretroviral therapy initiation | |  |  |
| <2000 | 1922(93) | 141(7) | 0.0287 |
| 2000–2009 | 1884(94) | 111(6) |  |
| 2010–2019 | 1090(92) | 94(8) |  |
| Systemic lupus erythematosus (End of follow-up) | |  |  |
| No | 4886(93) | 342–345(7) | 0.7391 |
| Yes | 10(71–91) | <5(9–29) |  |
| Non-AIDS-defining cancer (End of follow-up) | | |  |
| No | 2799(95) | 154(5) | <0.0001 |
| Yes | 2097(92) | 192(8) |  |
| Rheumatoid arthritis (End of follow-up) | |  |  |
| No | 4827(93) | 339(7) | 0.3559 |
| Yes | 69(91) | 7(9) |  |
| Inflammatory bowel disease (End of follow-up) | | |  |
| No | 4872(93) | 342–345(7) | 0.5996 |
| Yes | 24(86–96) | <5(4–14) |  |
| Chronic obstructive pulmonary disease (End of follow-up) | | |  |
| No | 4171(93) | 318(7) | 0.0006 |
| Yes | 725(96) | 28(4) |  |
| Diabetes mellitus (End of follow-up) | |  |  |
| No | 3915(94) | 269(6) | 0.3206 |
| Yes | 981(93) | 77(7) |  |
| Asthma (End of follow-up) | |  |  |
| No | 4139(93) | 291(7) | 0.8291 |
| Yes | 757(93) | 55(7) |  |
| Chronic kidney disease (End of follow-up) | |  |  |
| No | 3997(93) | 288(7) | 0.4568 |
| Yes | 899(94) | 58(6) |  |
| Cardiovascular disease (End of follow-up) | |  |  |
| No | 3834(93) | 268(7) | 0.7104 |
| Yes | 1062(93) | 78(7) |  |
| Substance use disorder (End of follow-up) | |  |  |
| No | 3204(92) | 297(8) | <0.0001 |
| Yes | 1692(97) | 49(3) |  |
| Hypertension (End of follow-up) | |  |  |
| No | 3216(95) | 170(5) | <0.0001 |
| Yes | 1680(91) | 176(9) |  |
| Mood/anxiety disorders (End of follow-up) | | |  |
| No | 1782(94) | 121(6) | 0.5940 |
| Yes | 3114(93) | 225(7) |  |
| Schizophrenia (End of follow-up) | |  |  |
| No | 4636(93) | 342–345(7) | <0.0001 |
| Yes | 260(98–99) | <5(1–2) |  |
| Traumatic brain injury (End of follow-up) | |  |  |
| No | 4183(93) | 318(7) | 0.0008 |
| Yes | 713(96) | 28(4) |  |
| Chronic liver disease (End of follow-up) | |  |  |
| No | 3736(93) | 279(7) | 0.0661 |
| Yes | 1160(95) | 67(5) |  |
| Transplant operation (End of follow-up) | |  |  |
| No | 4834(93) | 342–345(7) | 0.2599 |
| Yes | 62(94–98) | <5(2–6) |  |
| **Continuous Variables** | | | |
| Age (years)(End of follow-up) |  |  |  |
| Median | 58 | 62 | <0.0001 |
| 25^th^–75^th^ percentiles | 54–63 | 57–68 |  |
| Follow-up time (person-years) |  |  |  |
| Total | 39322 | 3742 | . |
| Median (25^th^–75^th^ percentiles) | 7(4–12) | 10(6–15) | <0.0001 |

**Note.** NA: Not applicable; p-value significance is set at 0.05; row percentages are shown.

# **Table S10.** Bivariable comparison of study variables by herpes zoster (HZ) vaccination status among people living without HIV aged 50 years and over.

| **Variable** | **Never Had HZ Vaccination** | **Completed HZ Vaccination** | **P-value** |
| --- | --- | --- | --- |
|  | **n = 5267**  n(%) | **n = 569**  n(%) |  |
| Sex |  |  |  |
| Male | 4785(90) | 503(10) | 0.0572 |
| Female | 482(88) | 66(12) |  |
| Downtown Eastside residence (End of follow-up) | |  |  |
| No | 5133(90) | 565–568(10) | 0.0004 |
| Yes | 134(97–99) | <5(1–3) |  |
| Herpes zoster infection (End of follow-up) |  |  |  |
| No | 4916(91) | 496(9) | <0.0001 |
| Yes | 351(83) | 73(17) |  |
| Herpes zoster infection (End of follow up) |  |  |  |
| No | 4916(91) | 496(9) | <0.0001 |
| 1 time | 327(83) | 67(17) |  |
| 2 or more times | 24(80) | 6(20) |  |
| HZ vaccine type (End of follow-up) |  |  |  |
| Live Zoster Vaccine (LZV) | NA | 400(100) | NA |
| Recombinant Zoster Vaccine (RZV) | NA | 203(100) |  |
| Both vaccines | NA | 34(100) |  |
| Systemic lupus erythematosus (End of follow-up) | |  |  |
| No | 5259(90) | 565–568(10) | 0.0058 |
| Yes | 8(67–89) | <5(11–33) |  |
| Non-AIDS-defining cancer (End of follow-up) | | |  |
| No | 3397(93) | 261(7) | <0.0001 |
| Yes | 1870(86) | 308(14) |  |
| Rheumatoid arthritis (End of follow-up) | |  |  |
| No | 5200(90) | 561(10) | 0.7876 |
| Yes | 67(89) | 8(11) |  |
| Inflammatory bowel disease (End of follow-up) | | |  |
| No | 5240(90) | 565–568(10) | 0.9631 |
| Yes | 27(87–96) | <5(4–13) |  |
| Chronic obstructive pulmonary disease (End of follow-up) | | |  |
| No | 4719(90) | 520(10) | 0.1800 |
| Yes | 548(92) | 49(8) |  |
| Diabetes mellitus (End of follow-up) | |  |  |
| No | 4314(91) | 434(9) | 0.0010 |
| Yes | 953(88) | 135(12) |  |
| Asthma (End of follow-up) | |  |  |
| No | 4557(91) | 473(9) | 0.0259 |
| Yes | 710(88) | 96(12) |  |
| Chronic kidney disease (End of follow-up) | |  |  |
| No | 4874(90) | 524(10) | 0.7006 |
| Yes | 393(90) | 45(10) |  |
| Cardiovascular disease (End of follow-up) | |  |  |
| No | 4247(91) | 407(9) | <0.0001 |
| Yes | 1020(86) | 162(14) |  |
| Substance use disorder (End of follow-up) | |  |  |
| No | 4149(90) | 484(10) | 0.0004 |
| Yes | 1118(93) | 85(7) |  |
| Hypertension (End of follow-up) | |  |  |
| No | 3354(92) | 288(8) | <0.0001 |
| Yes | 1913(87) | 281(13) |  |
| Mood/anxiety disorders (End of follow-up) | | |  |
| No | 2369(91) | 241(9) | 0.2319 |
| Yes | 2898(90) | 328(10) |  |
| Schizophrenia (End of follow-up) | |  |  |
| No | 5059(90) | 562(10) | 0.0011 |
| Yes | 208(97) | 7(3) |  |
| Traumatic brain injury (End of follow-up) | |  |  |
| No | 4637(90) | 517(10) | 0.0465 |
| Yes | 630(92) | 52(8) |  |
| Chronic liver disease (End of follow-up) | |  |  |
| No | 4875(90) | 542(10) | 0.0179 |
| Yes | 392(94) | 27(6) |  |
| Transplant operation (End of follow-up) | |  |  |
| No | 5195(90) | 561(10) | 0.9395 |
| Yes | 72(90) | 8(10) |  |
| **Continuous Variables** | | | |
| Age (years)(End of follow-up) |  |  |  |
| Median | 58 | 64 | <0.0001 |
| 25^th^–75^th^ percentiles | 54–63 | 59–69 |  |
| Follow-up time (person-years) |  |  |  |
| Total | 44975 | 7084 | . |
| Median (25^th^–75^th^ percentiles) | 8(4–12) | 13(8–18) | <0.0001 |

**Note.** NA: Not applicable; p-value significance is set at 0.05; row percentages are shown.

# **Table S11.** Standardized cumulative incidence rate (CIR) (per 1000 person-years) of herpes zoster (HZ) infection over the study period, by HIV status, for (A) the total population, and (B) those who received a complete course of HZ vaccination.

(A)

| **Variable** | **PLWH**  CIR(95%CI) | **PLWoH**  CIR(95%CI) |
| --- | --- | --- |
| Total Population | 14.49(13.18–15.80) | 6.91(5.76–8.06) |
| Sex |  |  |
| Male | 13.70(12.85–14.54) | 5.21(4.71–5.72) |
| Female | 15.22(12.82–17.63) | 7.22(6.42–8.02) |
| Index age | |  |
| ≥50 years | 14.50(12.45–16.56) | 9.11(7.43–10.79) |
| <50 years | 15.16(13.84–16.48) | 3.94(3.19–4.69) |

(B)

| **Variable** | **PLWH**  CIR(95%CI) | **PLWoH**  CIR(95%CI) |
| --- | --- | --- |
| Post-Live Zoster Vaccine (LZV) |  |  |
| Index age ≥50 years | 3.79(0.73–6.85) | 5.86(1.04–10.67) |
| Index age <50 years | 0 | 15.79(0.00–46.74)* |
| Post-Recombinant Zoster Vaccine (RZV) |  |  |
| Index age ≥50 years | 0 | 0 |
| Index age <50 years | 0 | 0 |

**Note:** *Among the 11 PLWoH <50 years of age who received the Live Zoster Vaccine, only one developed shingles; this results in a much higher incidence rate with a wide confidence interval; CI: confidence interval.

# **Table S12.** Number of people who developed herpes zoster (HZ) infection after completing vaccination for those aged ≥50 years, by HIV status.

| **Vaccine Type** | **PLWH**  **n=346** | **PLWoH**  **n=569** |
| --- | --- | --- |
| Live Zoster Vaccine (LZV) | 6/202* | 13/400 |
| Recombinant Zoster Vaccine (RZV) | 0/162 | 0/203 |

**Note.** *Values are the number of individuals who developed shingles/number of vaccinated individuals; PLWH: people living with HIV; PLWoH: people living without HIV

# **Table S13.** Bivariable comparison of study variables among people living with HIV, under 50 years old, who never versus ever developed herpes zoster (HZ) infection after being diagnosed with schizophrenia.

| **Variable** | **Never developed HZ after schizophrenia diagnosis** | **Ever developed HZ after schizophrenia diagnosis** | **P-value** |
| --- | --- | --- | --- |
|  | **n = 366**  n(%) | **n = 20**  n(%) |  |
| Sex |  |  |  |
| Male | 297(97) | 10(3) | 0.0008 |
| Female | 69(87) | 10(13) |  |
| Downtown Eastside residence (Index date) | |  |  |
| No | 315(94–95) | 16–19(5–6) | 0.6186 |
| Yes | 51(93–98) | <5(2–7) |  |
| Downtown Eastside residence (End of follow-up) | |  |  |
| No | 307(94–95) | 16–19(5–6) | 0.1814 |
| Yes | 59(94–98) | <5(2–6) |  |
| CD4 (cells/mm^3^)(Index date) |  |  |  |
| <50 | 22(85–96) | <5(4–15) | 0.2237 |
| 50–199 | 64(94–98) | <5(2–6) |  |
| 200–349 | 74(82–94) | 5–16(6–18) |  |
| ≥350 | 150(97–99) | <5(1–3) |  |
| Not measured | 56(93–98) | <5(2–7) |  |
| CD4 (cells/mm^3^)(End of follow-up) |  |  |  |
| <50 | 20(83–95) | <5(5–17) | 0.0282 |
| 50–199 | 38(100) | 0(0) |  |
| 200–349 | 51(84–91) | 5–10(9–16) |  |
| ≥350 | 199(96) | 8(4) |  |
| Not measured | 58(94–98) | <5(2–6) |  |
| Uncontrolled viremia (Index date) |  |  |  |
| Not suppressed | 218(92–95) | 12–18(5–8) | 0.1433 |
| Suppressed | 122(97–99) | <5(1–3) |  |
| Not measured | 26(87–96) | <5(4–13) |  |
| Uncontrolled viremia (End of follow-up) | |  |  |
| Not suppressed | 92(95) | 5(5) | 0.9982 |
| Suppressed | 236(95) | 13(5) |  |
| Not measured | 38(95) | <5(5) |  |
| Year of antiretroviral therapy initiation | |  |  |
| <2000 | 107(93) | 8(7) | 0.1324 |
| 2000–2009 | 123(92–94) | 8–11(6–8) |  |
| 2010–2019 | 136(97–99) | <5(1–3) |  |
| Ever vaccinated (End of follow-up) |  |  |  |
| No | 366(95) | 20(5) | NA |
| Yes | 0(0) | 0(0) |  |
| Systemic lupus erythematosus (Index date) | |  |  |
| No | 362–365(95) | 20(5) | 0.8149 |
| Yes | <5(100) | 0(0) |  |
| Systemic lupus erythematosus (End of follow-up) | |  |  |
| No | 362–365(95) | 20(5) | 0.7403 |
| Yes | <5(100) | 0(0) |  |
| Non–AIDS defining cancer (Index date) | |  |  |
| No | 331(97) | 12(3) | <0.0001 |
| Yes | 35(81) | 8(19) |  |
| Non–AIDS defining cancer (End of follow-up) | |  |  |
| No | 297(97) | 9(3) | 0.0001 |
| Yes | 69(86) | 11(14) |  |
| Rheumatoid arthritis (Index date) | |  |  |
| No | 362–365(95–96) | 16–19(4–5) | 0.0041 |
| Yes | <5(20–80) | <5(20–80) |  |
| Rheumatoid arthritis (End of follow-up) | |  |  |
| No | 362–365(95–96) | 16–19(4–5) | 0.0272 |
| Yes | <5(20–80) | <5(20–80) |  |
| Inflammatory bowel disease (Index date) | |  |  |
| No | 362–365(95) | 20(5) | 0.8149 |
| Yes | <5(100) | 0(0) |  |
| Inflammatory bowel disease (End of follow-up) | |  |  |
| No | 362–365(95) | 20(5) | 0.8149 |
| Yes | <5(100) | 0(0) |  |
| Chronic obstructive pulmonary disease (Index date) | |  |  |
| No | 361(95–96) | 16–19(4–5) | 0.2008 |
| Yes | 5(56–83) | <5(17–44) |  |
| Chronic obstructive pulmonary disease (End of follow-up) | |  |  |
| No | 338(95) | 16–19(5) | 0.6615 |
| Yes | 28(88–97) | <5(3–12) |  |
| Diabetes mellitus (Index date) |  |  |  |
| No | 351(95) | 20(5) | 0.3558 |
| Yes | 15(100) | 0(0) |  |
| Diabetes mellitus (End of follow-up) |  |  |  |
| No | 335(95) | 16–19(5) | 0.8117 |
| Yes | 31(89–97) | <5(3–11) |  |
| Asthma (Index date) |  |  |  |
| No | 319(94–95) | 16–19(5–6) | 0.3007 |
| Yes | 47(92–98) | <5(2–8) |  |
| Asthma (End of follow-up) |  |  |  |
| No | 300(94–95) | 16–19(5–6) | 0.1340 |
| Yes | 66(94–99) | <5(1–6) |  |
| Chronic kidney disease (Index date) | |  |  |
| No | 354(95) | 20(5) | 0.4107 |
| Yes | 12(100) | 0(0) |  |
| Chronic kidney disease (End of follow-up) | |  |  |
| No | 331(95) | 16–19(5) | 0.9484 |
| Yes | 35(90–97) | <5(3–10) |  |
| Cardiovascular disease (Index date) | |  |  |
| No | 352(95–96) | 16–19(4–5) | 0.0177 |
| Yes | 14(78–93) | <5(7–22) |  |
| Cardiovascular disease (End of follow-up) | |  |  |
| No | 325(94–95) | 16–19(5–6) | 0.2325 |
| Yes | 41(91–98) | <5(2–9) |  |
| Substance use disorder (Index date) | |  |  |
| No | 78(95–99) | <5(1–5) | 0.4997 |
| Yes | 288(94–95) | 16–19(5–6) |  |
| Substance use disorder (End of follow-up) | |  |  |
| No | 49(92–98) | <5(2–8) | 0.6631 |
| Yes | 317(94–95) | 16–19(5–6) |  |
| Hypertension (Index date) |  |  |  |
| No | 334(95) | 16–19(5) | 0.8469 |
| Yes | 32(89–97) | <5(3–11) |  |
| Hypertension (End of follow-up) | |  |  |
| No | 314(94–95) | 16–19(5–6) | 0.4738 |
| Yes | 52(93–98) | <5(2–7) |  |
| Mood/anxiety disorders (Index date) | |  |  |
| No | 56(93–98) | <5(2–7) | 0.2061 |
| Yes | 310(94–95) | 16–19(5–6) |  |
| Mood/anxiety disorders (End of follow-up) | |  |  |
| No | 34(89–97) | <5(3–11) | 0.5153 |
| Yes | 332(95) | 16–19(5) |  |
| Traumatic brain injury (Index date) | |  |  |
| No | 303(94–95) | 16–19(5–6) | 0.4013 |
| Yes | 63(94–98) | <5(2–6) |  |
| Traumatic brain injury (End of follow-up) | |  |  |
| No | 267(93–94) | 16–19(6–7) | 0.2340 |
| Yes | 99(96–99) | <5(1–4) |  |
| Chronic liver disease (Index date) | |  |  |
| No | 296(94) | 20(6) | 0.0306 |
| Yes | 70(100) | 0(0) |  |
| Chronic liver disease (End of follow-up) | |  |  |
| No | 229(95) | 12(5) | 0.8174 |
| Yes | 137(94) | 8(6) |  |
| Transplant operation (Index date) |  |  |  |
| No | 366(95) | 20(5) | NA. |
| Yes | 0(0) | 0(0) |  |
| Transplant operation (End of follow-up) | |  |  |
| No | 362–365(95) | 20(5) | 0.6844 |
| Yes | <5(100) | 0(0) |  |
| **Continuous Variables** | | | |
| Age (years)(Index date) |  |  |  |
| Median | 39 | 37 | 0.3863 |
| 25^th^–75^th^ percentiles | 33–44 | 30–42 |  |
| Age (years)(End of follow-up) |  |  |  |
| Median | 47 | 48 | 0.5268 |
| 25^th^–75^th^ percentiles | 41–49 | 44–49 |  |

**Note.** NA: not applicable; P-value significance is set at 0.05; row percentages are shown.

# **Table S14.** Bivariable comparison of study variables among people living with HIV, aged 50 years and over, who never versus ever developed herpes zoster (HZ) infection after being diagnosed with schizophrenia.

| **Variable** | **Never developed HZ after schizophrenia diagnosis** | **Ever developed HZ after schizophrenia diagnosis** | **P-value** |
| --- | --- | --- | --- |
|  | **n = 241**  n(%) | **n = 22**  n(%) |  |
| Sex |  |  |  |
| Male | 199(90–92) | 18–21(8–10) | 0.9289 |
| Female | 42(91–98) | <5(2–9) |  |
| Downtown Eastside residence (Index date) | |  |  |
| No | 214(91–92) | 18–21(8–9) | 0.7620 |
| Yes | 27(87–91) | <5(9–13) |  |
| Downtown Eastside residence (End of follow-up) | |  |  |
| No | 218(91) | 22(9) | 0.1293 |
| Yes | 23(100) | 0(0) |  |
| CD4 (cells/mm^3^)(Index date) |  |  |  |
| <50 | 17(81–94) | <5(6–19) | 0.3041 |
| 50–199 | 36(90–97) | <5(3–10) |  |
| 200–349 | 50(93–98) | <5(2–7) |  |
| ≥350 | 111(90–96) | 5–13(4–10) |  |
| Not measured | 27(82) | 6(18) |  |
| CD4 (cells/mm^3^)(End of follow-up) |  |  |  |
| <50 | <5(20–80) | <5(20–80) | 0.7479 |
| 50–199 | 29(88–97) | <5(3–12) |  |
| 200–349 | 42(91–98) | <5(2–9) |  |
| ≥350 | 129(90–96) | 5–14(4–10) |  |
| Not measured | 37(88) | 5(12) |  |
| Uncontrolled viremia (Index date) |  |  |  |
| Not suppressed | 72(88) | 10(12) | 0.3165 |
| Suppressed | 157(93–95) | 8–11(5–7) |  |
| Not measured | 12(75–92) | <5(8–25) |  |
| Uncontrolled viremia (End of follow-up) | |  |  |
| Not suppressed | 35(95) | <5(5) | 0.6325 |
| Suppressed | 187(90–93) | 14–20(7–10) |  |
| Not measured | 19(83–95) | <5(5–17) |  |
| Year of antiretroviral therapy initiation | |  |  |
| <2000 | 104(92) | 9(8) | 0.8027 |
| 2000–2009 | 94(89–91) | 9–12(9–11) |  |
| 2010–2019 | 43(91–98) | <5(2–9) |  |
| Ever vaccinated (End of follow-up) |  |  |  |
| No | 237–240(92–93) | 18–21(7–8) | 0.0328 |
| Yes | <5(20–80) | <5(20–80) |  |
| Systemic lupus erythematosus (Index date) | |  |  |
| No | 237–240(92) | 22(8) | 0.7621 |
| Yes | <5(100) | 0(0) |  |
| Systemic lupus erythematosus (End of follow-up) | |  |  |
| No | 237–240(92) | 22(8) | 0.6680 |
| Yes | <5(100) | 0(0) |  |
| Non–AIDS defining cancer (Index date) | |  |  |
| No | 181(90–91) | 18–21(9–10) | 0.2362 |
| Yes | 60(94–98) | <5(2–6) |  |
| Non–AIDS defining cancer (End of follow-up) | |  |  |
| No | 153(91) | 15(9) | 0.6607 |
| Yes | 88(93) | 7(7) |  |
| Rheumatoid arthritis (Index date) | |  |  |
| No | 237–240(92) | 22(8) | 0.5426 |
| Yes | <5(100) | 0(0) |  |
| Rheumatoid arthritis (End of follow-up) | |  |  |
| No | 235(91) | 22(9) | 0.4541 |
| Yes | 6(100) | 0(0) |  |
| Inflammatory bowel disease (Index date) | |  |  |
| No | 241(92) | 22(8) | NA |
| Yes | 0(0) | 0(0) |  |
| Inflammatory bowel disease (End of follow-up) | |  |  |
| No | 241(92) | 22(8) | NA |
| Yes | 0(0) | 0(0) |  |
| Chronic obstructive pulmonary disease (Index date) | |  |  |
| No | 212(91–92) | 18–21(8–9) | 0.8257 |
| Yes | 29(88–97) | <5(3–12) |  |
| Chronic obstructive pulmonary disease (End of follow-up) | |  |  |
| No | 188(93) | 14(7) | 0.1263 |
| Yes | 53(87) | 8(13) |  |
| Diabetes mellitus (Index date) |  |  |  |
| No | 206(91–92) | 18–21(8–9) | 0.6439 |
| Yes | 35(90–97) | <5(3–10) |  |
| Diabetes mellitus (End of follow-up) |  |  |  |
| No | 186(92) | 16(8) | 0.6358 |
| Yes | 55(90) | 6(10) |  |
| Asthma (Index date) |  |  |  |
| No | 201(91–92) | 18–21(8–9) | 0.7192 |
| Yes | 40(91–98) | <5(2–9) |  |
| Asthma (End of follow-up) |  |  |  |
| No | 191(92) | 17(8) | 0.8269 |
| Yes | 50(91) | 5(9) |  |
| Chronic kidney disease (Index date) | |  |  |
| No | 214(91) | 22(9) | 0.0975 |
| Yes | 27(100) | 0(0) |  |
| Chronic kidney disease (End of follow-up) | |  |  |
| No | 184(92) | 17(8) | 0.9221 |
| Yes | 57(92) | 5(8) |  |
| Cardiovascular disease (Index date) | |  |  |
| No | 201(91–92) | 18–21(8–9) | 0.7192 |
| Yes | 40(91–98) | <5(2–9) |  |
| Cardiovascular disease (End of follow-up) | |  |  |
| No | 177(91) | 17(9) | 0.6960 |
| Yes | 64(93) | 5(7) |  |
| Substance use disorder (Index date) | |  |  |
| No | 62(86) | 10(14) | 0.0470 |
| Yes | 179(94) | 12(6) |  |
| Substance use disorder (End of follow-up) | |  |  |
| No | 51(85) | 9(15) | 0.0346 |
| Yes | 190(94) | 13(6) |  |
| Hypertension (Index date) |  |  |  |
| No | 192(92) | 17(8) | 0.7900 |
| Yes | 49(91) | 5(9) |  |
| Hypertension (End of follow-up) | |  |  |
| No | 172(91) | 16(9) | 0.8926 |
| Yes | 69(92) | 6(8) |  |
| Mood/anxiety disorders (Index date) | |  |  |
| No | 33(89–97) | <5(3–11) | 0.5621 |
| Yes | 208(91–92) | 18–21(8–9) |  |
| Mood/anxiety disorders (End of follow-up) | |  |  |
| No | 24(86–96) | <5(4–14) | 0.2313 |
| Yes | 217(91–92) | 18–21(8–9) |  |
| Traumatic brain injury (Index date) | |  |  |
| No | 179(90–91) | 18–21(9–10) | 0.4346 |
| Yes | 62(94–98) | <5(2–6) |  |
| Traumatic brain injury (End of follow-up) | |  |  |
| No | 162(91) | 17(9) | 0.3330 |
| Yes | 79(94) | 5(6) |  |
| Chronic liver disease (Index date) | |  |  |
| No | 162(92) | 15(8) | 0.9266 |
| Yes | 79(92) | 7(8) |  |
| Chronic liver disease (End of follow-up) | |  |  |
| No | 140(93) | 10(7) | 0.2517 |
| Yes | 101(89) | 12(11) |  |
| Transplant operation (Index date) |  |  |  |
| No | 237–240(92–93) | 18–21(7–8) | 0.0002 |
| Yes | <5(20–80) | <5(20–80) |  |
| Transplant operation (End of follow-up) | |  |  |
| No | 237–240(92–93) | 18–21(7–8) | 0.0002 |
| Yes | <5(20–80) | <5(20–80) |  |
| **Continuous Variables** | | | |
| Age (years)(Index date) |  |  |  |
| Median | 50 | 54 | 0.0009 |
| 25^th^–75^th^ percentiles | 50–54 | 50–60 |  |
| Age(years)(End of follow-up) |  |  |  |
| Median | 56 | 63 | 0.0004 |
| 25^th^–75^th^ percentiles | 53–61 | 56–68 |  |

**Note.** P-value significance is set at 0.05; row percentages are shown.

# **Table S15.** Bivariable comparison of study variables among people living with HIV, under 50 years old, who never versus ever developed herpes zoster (HZ) infection after being diagnosed with substance use disorder (SUD).

| **Variable** | **Never developed HZ after SUD diagnosis** | **Ever developed HZ after SUD diagnosis** | **P-value** |
| --- | --- | --- | --- |
|  | **n = 2307**  n(%) | **n = 250**  n(%) |  |
| Sex |  |  |  |
| Male | 1785(91) | 170(9) | 0.0009 |
| Female | 522(87) | 80(13) |  |
| Downtown Eastside residence (Index date) | |  |  |
| No | 2119(90) | 233(10) | 0.4556 |
| Yes | 188(92) | 17(8) |  |
| Downtown Eastside residence (End of follow-up) | |  |  |
| No | 2057(90) | 233(10) | 0.0474 |
| Yes | 250(94) | 17(6) |  |
| CD4 (cells/mm^3^)(Index date) |  |  |  |
| <50 | 169(89) | 21(11) | 0.1438 |
| 50–199 | 381(90) | 42(10) |  |
| 200–349 | 451(89) | 56(11) |  |
| ≥350 | 839(92) | 71(8) |  |
| Not measured | 467(89) | 60(11) |  |
| CD4 (cells/mm^3^)(End of follow-up) |  |  |  |
| <50 | 164(89) | 20(11) | 0.6317 |
| 50–199 | 313(88) | 41(12) |  |
| 200–349 | 367(92) | 34(8) |  |
| ≥350 | 1014(90) | 110(10) |  |
| Not measured | 449(91) | 45(9) |  |
| Uncontrolled viremia (Index date) |  |  |  |
| Not suppressed | 1425(88) | 202(12) | <0.0001 |
| Suppressed | 636(96) | 25(4) |  |
| Not measured | 246(91) | 23(9) |  |
| Uncontrolled viremia (End of follow-up) | |  |  |
| Not suppressed | 683(90) | 76(10) | 0.9153 |
| Suppressed | 1337(90) | 145(10) |  |
| Not measured | 287(91) | 29(9) |  |
| Year of antiretroviral therapy initiation | |  |  |
| <2000 | 699(88) | 97(12) | <0.0001 |
| 2000–2009 | 861(89) | 111(11) |  |
| 2010–2019 | 747(95) | 42(5) |  |
| Ever vaccinated (End of follow-up) |  |  |  |
| No | 2303–2306(90) | 246–249(10) | 0.4411 |
| Yes | <5(20–80) | <5(20–80) |  |
| Systemic lupus erythematosus (Index date) | |  |  |
| No | 2303–2306(90) | 250(10) | 0.5100 |
| Yes | <5(100) | 0(0) |  |
| Systemic lupus erythematosus (End of follow-up) | |  |  |
| No | 2301(90) | 250(10) | 0.4195 |
| Yes | 6(100) | 0(0) |  |
| Non–AIDS defining cancer (Index date) | |  |  |
| No | 2052(90) | 222(10) | 0.9441 |
| Yes | 255(90) | 28(10) |  |
| Non–AIDS defining cancer (End of follow-up) | |  |  |
| No | 1820(91) | 173(9) | 0.0004 |
| Yes | 487(86) | 77(14) |  |
| Rheumatoid arthritis (Index date) | |  |  |
| No | 2293(90) | 246–249(10) | 0.0744 |
| Yes | 14(78–93) | <5(7–22) |  |
| Rheumatoid arthritis (End of follow-up) | |  |  |
| No | 2286(90) | 245(10) | 0.1028 |
| Yes | 21(81) | 5(19) |  |
| Inflammatory bowel disease (Index date) | |  |  |
| No | 2300(90) | 250(10) | 0.3831 |
| Yes | 7(100) | 0(0) |  |
| Inflammatory bowel disease (End of follow-up) | |  |  |
| No | 2298(90) | 250(10) | 0.3225 |
| Yes | 9(100) | 0(0) |  |
| Chronic obstructive pulmonary disease (Index date) | |  |  |
| No | 2266(90) | 246–249(10) | 0.8396 |
| Yes | 41(91–98) | <5(2–9) |  |
| Chronic obstructive pulmonary disease (End of follow-up) | |  |  |
| No | 2142(91) | 221(9) | 0.0116 |
| Yes | 165(85) | 29(15) |  |
| Diabetes mellitus (Index date) |  |  |  |
| No | 2224(90) | 246–249(10) | 0.0979 |
| Yes | 83(95–99) | <5(1–5) |  |
| Diabetes mellitus (End of follow-up) |  |  |  |
| No | 2148(91) | 224(9) | 0.0420 |
| Yes | 159(86) | 26(14) |  |
| Asthma (Index date) |  |  |  |
| No | 2056(91) | 214(9) | 0.0940 |
| Yes | 251(87) | 36(13) |  |
| Asthma (End of follow-up) |  |  |  |
| No | 1925(91) | 185(9) | 0.0002 |
| Yes | 382(85) | 65(15) |  |
| Chronic kidney disease (Index date) | |  |  |
| No | 2241(90) | 246–249(10) | 0.1237 |
| Yes | 66(94–99) | <5(1–6) |  |
| Chronic kidney disease (End of follow-up) | |  |  |
| No | 2065(91) | 209(9) | 0.0047 |
| Yes | 242(86) | 41(14) |  |
| Cardiovascular disease (Index date) | |  |  |
| No | 2216(90) | 240(10) | 0.9660 |
| Yes | 91(90) | 10(10) |  |
| Cardiovascular disease (End of follow-up) | |  |  |
| No | 2082(91) | 212(9) | 0.0071 |
| Yes | 225(86) | 38(14) |  |
| Hypertension (Index date) |  |  |  |
| No | 2179(90) | 234(10) | 0.5790 |
| Yes | 128(89) | 16(11) |  |
| Hypertension (End of follow-up) | |  |  |
| No | 2057(91) | 211(9) | 0.0239 |
| Yes | 250(87) | 39(13) |  |
| Mood/anxiety disorders (Index date) |  |  |  |
| No | 948(92) | 85(8) | 0.0300 |
| Yes | 1359(89) | 165(11) |  |
| Mood/anxiety disorders (End of follow-up) | |  |  |
| No | 606(94) | 41(6) | 0.0007 |
| Yes | 1701(89) | 209(11) |  |
| Schizophrenia (Index date) | |  |  |
| No | 2128(90) | 238(10) | 0.0910 |
| Yes | 179(94) | 12(6) |  |
| Schizophrenia (End of follow-up) |  |  |  |
| No | 2002(90) | 220(10) | 0.5869 |
| Yes | 305(91) | 30(9) |  |
| Traumatic brain injury (Index date) | |  |  |
| No | 2059(90) | 225(10) | 0.7153 |
| Yes | 248(91) | 25(9) |  |
| Traumatic brain injury (End of follow-up) | |  |  |
| No | 1858(90) | 196(10) | 0.4193 |
| Yes | 449(89) | 54(11) |  |
| Chronic liver disease (Index date) | |  |  |
| No | 2067(90) | 230(10) | 0.2324 |
| Yes | 240(92) | 20(8) |  |
| Chronic liver disease (End of follow-up) | |  |  |
| No | 1562(92) | 143(8) | 0.0008 |
| Yes | 745(87) | 107(13) |  |
| Transplant operation (Index date) |  |  |  |
| No | 2299(90) | 250(10) | 0.3511 |
| Yes | 8(100) | 0(0) |  |
| Transplant operation (End of follow-up) | |  |  |
| No | 2286(90) | 246–249(10) | 0.2924 |
| Yes | 21(84–95) | <5(5–16) |  |
| **Continuous Variables** | | | |
| Age (years)(Index date) |  |  |  |
| Median | 38 | 36 | <0.0001 |
| 25^th^–75^th^ percentiles | 32–43 | 31–40 |  |
| Age (years)(End of follow-up) |  |  |  |
| Median | 47 | 49 | 0.0008 |
| 25^th^–75^th^ percentiles | 40–49 | 44–49 |  |

**Note.** P-value significance is set at 0.05; row percentages are shown.

# **Table S16.** Bivariable comparison of study variables among people living with HIV, aged 50 years and over, who never versus ever developed herpes zoster (HZ) infection after being diagnosed with substance use disorder (SUD).

| **Variable** | **Never developed HZ after SUD diagnosis** | **Ever developed HZ after SUD diagnosis** | **P-value** |
| --- | --- | --- | --- |
|  | **n = 1634**  n(%) | **n = 115**  n(%) |  |
| Sex |  |  |  |
| Male | 1393(94) | 95(6) | 0.4421 |
| Female | 241(92) | 20(8) |  |
| Downtown Eastside residence (Index date) | |  |  |
| No | 1471(93) | 109(7) | 0.0951 |
| Yes | 163(96) | 6(4) |  |
| Downtown Eastside residence (End of follow-up) | |  |  |
| No | 1472(93) | 111–114(7) | 0.0037 |
| Yes | 162(98–99) | <5(1–2) |  |
| CD4 (cells/mm^3^)(Index date) |  |  |  |
| <50 | 76(90) | 8(10) | 0.0386 |
| 50–199 | 267(93) | 20(7) |  |
| 200–349 | 339(91) | 32(9) |  |
| ≥350 | 747(95) | 36(5) |  |
| Not measured | 205(92) | 19(8) |  |
| CD4 (cells/mm^3^)(End of follow-up) |  |  |  |
| <50 | 59(94–98) | <5(2–6) | 0.6125 |
| 50–199 | 210(91–92) | 19–22(8–9) |  |
| 200–349 | 262(93) | 20(7) |  |
| ≥350 | 793(94) | 50(6) |  |
| Not measured | 310(93) | 22(7) |  |
| Uncontrolled viremia (Index date) |  |  |  |
| Not suppressed | 531(90) | 62(10) | <0.0001 |
| Suppressed | 1000(96) | 46(4) |  |
| Not measured | 103(94) | 7(6) |  |
| Uncontrolled viremia (End of follow-up) | |  |  |
| Not suppressed | 260(91) | 26(9) | 0.1710 |
| Suppressed | 1209(94) | 78(6) |  |
| Not measured | 165(94) | 11(6) |  |
| Year of antiretroviral therapy initiation | |  |  |
| <2000 | 638(93) | 50(7) | 0.6056 |
| 2000–2009 | 680(94) | 43(6) |  |
| 2010–2019 | 316(93) | 22(7) |  |
| Ever vaccinated (End of follow-up) |  |  |  |
| No | 1591(94) | 110(6) | 0.2406 |
| Yes | 43(90) | 5(10) |  |
| Systemic lupus erythematosus (Index date) | |  |  |
| No | 1630–1633(93) | 115(7) | 0.6456 |
| Yes | <5(100) | 0(0) |  |
| Systemic lupus erythematosus (End of follow-up) | |  |  |
| No | 1630–1633(93) | 115(7) | 0.5953 |
| Yes | <5(100) | 0(0) |  |
| Non–AIDS defining cancer (Index date) | |  |  |
| No | 1187(93) | 93(7) | 0.0543 |
| Yes | 447(95) | 22(5) |  |
| Non–AIDS defining cancer (End of follow-up) | |  |  |
| No | 992(93) | 69(7) | 0.8803 |
| Yes | 642(93) | 46(7) |  |
| Rheumatoid arthritis (Index date) | |  |  |
| No | 1617(94) | 111–114(6) | 0.1263 |
| Yes | 17(81–94) | <5(6–19) |  |
| Rheumatoid arthritis (End of follow-up) | |  |  |
| No | 1608(94) | 111–114(6) | 0.1319 |
| Yes | 26(87–96) | <5(4–13) |  |
| Inflammatory bowel disease (Index date) | |  |  |
| No | 1628(93) | 115(7) | 0.5151 |
| Yes | 6(100) | 0(0) |  |
| Inflammatory bowel disease (End of follow-up) | |  |  |
| No | 1627(93) | 115(7) | 0.4819 |
| Yes | 7(100) | 0(0) |  |
| Chronic obstructive pulmonary disease (Index date) | |  |  |
| No | 1430(93) | 102(7) | 0.7105 |
| Yes | 204(94) | 13(6) |  |
| Chronic obstructive pulmonary disease (End of follow-up) | |  |  |
| No | 1249(94) | 82(6) | 0.2121 |
| Yes | 385(92) | 33(8) |  |
| Diabetes mellitus (Index date) |  |  |  |
| No | 1443(93) | 102(7) | 0.9011 |
| Yes | 191(94) | 13(6) |  |
| Diabetes mellitus (End of follow-up) |  |  |  |
| No | 1345(94) | 89(6) | 0.1843 |
| Yes | 289(92) | 26(8) |  |
| Asthma (Index date) |  |  |  |
| No | 1391(93) | 99(7) | 0.7797 |
| Yes | 243(94) | 16(6) |  |
| Asthma (End of follow-up) |  |  |  |
| No | 1323(94) | 86(6) | 0.1053 |
| Yes | 311(91) | 29(9) |  |
| Chronic kidney disease (Index date) | |  |  |
| No | 1441(93) | 110(7) | 0.0146 |
| Yes | 193(97) | 5(3) |  |
| Chronic kidney disease (End of follow-up) | |  |  |
| No | 1263(94) | 79(6) | 0.0349 |
| Yes | 371(91) | 36(9) |  |
| Cardiovascular disease (Index date) | |  |  |
| No | 1403(93) | 103(7) | 0.2672 |
| Yes | 231(95) | 12(5) |  |
| Cardiovascular disease (End of follow-up) | |  |  |
| No | 1237(94) | 75(6) | 0.0121 |
| Yes | 397(91) | 40(9) |  |
| Hypertension (Index date) |  |  |  |
| No | 1302(93) | 101(7) | 0.0341 |
| Yes | 332(96) | 14(4) |  |
| Hypertension (End of follow-up) | |  |  |
| No | 1143(94) | 75(6) | 0.2859 |
| Yes | 491(92) | 40(8) |  |
| Mood/anxiety disorders (Index date) |  |  |  |
| No | 470(93) | 35(7) | 0.7023 |
| Yes | 1164(94) | 80(6) |  |
| Mood/anxiety disorders (End of follow-up) | |  |  |
| No | 367(93) | 27(7) | 0.8006 |
| Yes | 1267(94) | 88(6) |  |
| Schizophrenia (Index date) | |  |  |
| No | 1481(93) | 107(7) | 0.3881 |
| Yes | 153(95) | 8(5) |  |
| Schizophrenia (End of follow-up) |  |  |  |
| No | 1447(94) | 99(6) | 0.4244 |
| Yes | 187(92) | 16(8) |  |
| Traumatic brain injury (Index date) | |  |  |
| No | 1336(93) | 105(7) | 0.0094 |
| Yes | 298(97) | 10(3) |  |
| Traumatic brain injury (End of follow-up) | |  |  |
| No | 1220(93) | 91(7) | 0.2852 |
| Yes | 414(95) | 24(5) |  |
| Chronic liver disease (Index date) | |  |  |
| No | 1174(93) | 84(7) | 0.7828 |
| Yes | 460(94) | 31(6) |  |
| Chronic liver disease (End of follow-up) | |  |  |
| No | 962(95) | 55(5) | 0.0203 |
| Yes | 672(92) | 60(8) |  |
| Transplant operation (Index date) |  |  |  |
| No | 1620(93) | 111–114(7) | 0.9885 |
| Yes | 14(78–93) | <5(7–22) |  |
| Transplant operation (End of follow-up) | |  |  |
| No | 1605(93) | 111–114(7) | 0.5190 |
| Yes | 29(88–97) | <5(3–12) |  |
| **Continuous Variables** | | | |
| Age (years)(Index date) |  |  |  |
| Median | 50 | 50 | 0.9787 |
| 25^th^–75^th^ percentiles | 50–53 | 50–53 |  |
| Age (years)(End of follow-up) |  |  |  |
| Median | 56 | 60 | <0.0001 |
| 25^th^–75^th^ percentiles | 53–61 | 56–64 |  |

**Note.** P-value significance is set at 0.05; row percentages are shown.

# **Table S17.** Bivariable comparison of study variables among people living with HIV, under 50 years old, who never versus ever developed herpes zoster (HZ) infection after residing in Vancouver’s Downtown Eastside (DTES).

| **Variable** | **Never developed HZ after DTES residence** | **Ever developed HZ after DTES residence** | **P-value** |
| --- | --- | --- | --- |
|  | **n = 818**  n(%) | **n = 61**  n(%) |  |
| Sex |  |  |  |
| Male | 614(94) | 42(6) | 0.2824 |
| Female | 204(91) | 19(9) |  |
| CD4 (cells/mm^3^)(Index date) |  |  |  |
| <50 | 43(88) | 6(12) | 0.4200 |
| 50–199 | 121(96) | 5(4) |  |
| 200–349 | 160(93) | 12(7) |  |
| ≥350 | 263(93) | 20(7) |  |
| Not measured | 231(93) | 18(7) |  |
| CD4 (cells/mm^3^)(End of follow-up) |  |  |  |
| <50 | 59(94–98) | <5(2–6) | 0.7700 |
| 50–199 | 120(90–92) | 11–14(8–10) |  |
| 200–349 | 142(93) | 11(7) |  |
| ≥350 | 376(93) | 28(7) |  |
| Not measured | 121(95) | 7(5) |  |
| Uncontrolled viremia (Index date) |  |  |  |
| Not suppressed | 566(92) | 48(8) | 0.0637 |
| Suppressed | 112(97–99) | <5(1–3) |  |
| Not measured | 140(92–94) | 9–12(6–8) |  |
| Uncontrolled viremia (End of follow-up) | |  |  |
| Not suppressed | 247(95) | 14(5) | 0.4678 |
| Suppressed | 464(92) | 39(8) |  |
| Not measured | 107(93) | 8(7) |  |
| Year of antiretroviral therapy initiation | |  |  |
| <2000 | 227(91) | 23(9) | 0.0513 |
| 2000–2009 | 343(92) | 28(8) |  |
| 2010–2019 | 248(96) | 10(4) |  |
| Ever vaccinated (End of follow-up) |  |  |  |
| No | 818(93) | 61(7) | NA |
| Yes | 0(0) | 0(0) |  |
| Systemic lupus erythematosus (Index date) | |  |  |
| No | 814–817(93) | 61(7) | 0.7847 |
| Yes | <5(100) | 0(0) |  |
| Systemic lupus erythematosus (End of follow-up) | |  |  |
| No | 814–817(93) | 61(7) | 0.7847 |
| Yes | <5(100) | 0(0) |  |
| Non–AIDS defining cancer (Index date) | |  |  |
| No | 774(93) | 57–60(7) | 0.4773 |
| Yes | 44(92–98) | <5(2–8) |  |
| Non–AIDS defining cancer (End of follow-up) | |  |  |
| No | 672(95) | 39(5) | 0.0005 |
| Yes | 146(87) | 22(13) |  |
| Rheumatoid arthritis (Index date) | |  |  |
| No | 814–817(93) | 61(7) | 0.6990 |
| Yes | <5(100) | 0(0) |  |
| Rheumatoid arthritis (End of follow-up) | |  |  |
| No | 813(93) | 61(7) | 0.5400 |
| Yes | 5(100) | 0(0) |  |
| Inflammatory bowel disease (Index date) | |  |  |
| No | 814–817(93) | 61(7) | 0.7847 |
| Yes | <5(100) | 0(0) |  |
| Inflammatory bowel disease (End of follow-up) | |  |  |
| No | 814–817(93) | 61(7) | 0.7847 |
| Yes | <5(100) | 0(0) |  |
| Chronic obstructive pulmonary disease (Index date) | |  |  |
| No | 812(93) | 61(7) | 0.5021 |
| Yes | 6(100) | 0(0) |  |
| Chronic obstructive pulmonary disease (End of follow-up) | |  |  |
| No | 752(93) | 55(7) | 0.6272 |
| Yes | 66(92) | 6(8) |  |
| Diabetes mellitus (Index date) |  |  |  |
| No | 805(93) | 57–60(7) | 0.9759 |
| Yes | 13(76–93) | <5(7–24) |  |
| Diabetes mellitus (End of follow-up) |  |  |  |
| No | 768(94) | 52(6) | 0.0093 |
| Yes | 50(85) | 9(15) |  |
| Asthma (Index date) |  |  |  |
| No | 753(93) | 54(7) | 0.3322 |
| Yes | 65(90) | 7(10) |  |
| Asthma (End of follow-up) |  |  |  |
| No | 685(94) | 42(6) | 0.0030 |
| Yes | 133(88) | 19(13) |  |
| Chronic kidney disease (Index date) | |  |  |
| No | 811(93) | 57–60(7) | 0.5341 |
| Yes | 7(64–88) | <5(12–36) |  |
| Chronic kidney disease (End of follow-up) | |  |  |
| No | 732(94) | 45(6) | 0.0002 |
| Yes | 86(84) | 16(16) |  |
| Cardiovascular disease (Index date) | |  |  |
| No | 807(93) | 57–60(7) | 0.8483 |
| Yes | 11(73–92) | <5(8–27) |  |
| Cardiovascular disease (End of follow-up) | |  |  |
| No | 749(94) | 50(6) | 0.0119 |
| Yes | 69(86) | 11(14) |  |
| Substance use disorder (Index date) |  |  |  |
| No | 442(93) | 32(7) | 0.8118 |
| Yes | 376(93) | 29(7) |  |
| Substance use disorder (End of follow) |  |  |  |
| No | 220(94) | 14(6) | 0.5014 |
| Yes | 598(93) | 47(7) |  |
| Hypertension (Index date) |  |  |  |
| No | 802(93) | 57–60(7) | 0.8625 |
| Yes | 16(80–94) | <5(6–20) |  |
| Hypertension (End of follow-up) | |  |  |
| No | 757(94) | 49(6) | 0.0009 |
| Yes | 61(84) | 12(16) |  |
| Mood/anxiety disorders (Index date) |  |  |  |
| No | 514(94) | 31(6) | 0.0621 |
| Yes | 304(91) | 30(9) |  |
| Mood/anxiety disorders (End of follow-up) | |  |  |
| No | 305(95) | 15(5) | 0.0468 |
| Yes | 513(92) | 46(8) |  |
| Schizophrenia (Index date) | |  |  |
| No | 767(93) | 57–60(7) | 0.3494 |
| Yes | 51(93–98) | <5(2–7) |  |
| Schizophrenia (End of follow-up) |  |  |  |
| No | 689(93) | 53(7) | 0.5812 |
| Yes | 129(94) | 8(6) |  |
| Traumatic brain injury (Index date) | |  |  |
| No | 763(93) | 55(7) | 0.3561 |
| Yes | 55(90) | 6(10) |  |
| Traumatic brain injury (End of follow-up) | |  |  |
| No | 667(93) | 47(7) | 0.3862 |
| Yes | 151(92) | 14(8) |  |
| Chronic liver disease (Index date) | |  |  |
| No | 786(93) | 57–60(7) | 0.6983 |
| Yes | 32(89–97) | <5(3–11) |  |
| Chronic liver disease (End of follow-up) | |  |  |
| No | 541(95) | 29(5) | 0.0033 |
| Yes | 277(90) | 32(10) |  |
| Transplant operation (Index date) |  |  |  |
| No | 814–817(93) | 61(7) | 0.6990 |
| Yes | <5(100) | 0(0) |  |
| Transplant operation (End of follow-up) | |  |  |
| No | 810(93) | 57–60(7) | 0.6206 |
| Yes | 8(67–89) | <5(11–33) |  |
| **Continuous Variables** | | | |
| Age (years)(Index date) |  |  |  |
| Median | 35 | 33 | 0.0258 |
| 25^th^–75^th^ percentiles | 29–40 | 30–37 |  |
| Age (years)(End of follow-up) |  |  |  |
| Median | 48 | 47 | 0.6434 |
| 25^th^–75^th^ percentiles | 41–49 | 42–49 |  |

**Note.** NA: Not applicable; p-value significance is set at 0.05; row percentages are shown.

# **Table S18.** Bivariable comparison of study variables among people living with HIV, aged 50 years and over, who never versus ever developed herpes zoster (HZ) infection after residing in Vancouver’s Downtown Eastside (DTES).

| **Variable** | **Never developed HZ after DTES residence** | **Ever developed HZ after DTES residence** | **P-value** |
| --- | --- | --- | --- |
|  | **n = 409**  n(%) | **n = 27**  n(%) |  |
| Sex |  |  |  |
| Male | 344(93–94) | 23–26(6–7) | 0.5072 |
| Female | 65(94–98) | <5(2–6) |  |
| CD4 (cells/mm^3^)(Index date) |  |  |  |
| <50 | 19(83–95) | <5(5–17) | 0.4411 |
| 50–199 | 68(94–99) | <5(1–6) |  |
| 200–349 | 119(94–96) | 5–8(4–6) |  |
| ≥350 | 168(93) | 12(7) |  |
| Not measured | 35(88) | 5(13) |  |
| CD4 (cells/mm^3^)(End of follow-up) |  |  |  |
| <50 | 13(100) | 0(0) | 0.5499 |
| 50–199 | 64(94–98) | <5(2–6) |  |
| 200–349 | 104(96–99) | <5(1–4) |  |
| ≥350 | 187(89–93) | 15–24(7–11) |  |
| Not measured | 41(91–98) | <5(2–9) |  |
| Uncontrolled viremia (Index date) |  |  |  |
| Not suppressed | 191(88–91) | 19–25(9–12) | <0.0001 |
| Suppressed | 196(98–99) | <5(1–2) |  |
| Not measured | 22(85–96) | <5(4–15) |  |
| Uncontrolled viremia (End of follow-up) | |  |  |
| Not suppressed | 67(92) | 6(8) | 0.7334 |
| Suppressed | 309(94–95) | 17–20(5–6) |  |
| Not measured | 33(89–97) | <5(3–11) |  |
| Year of antiretroviral therapy initiation | |  |  |
| <2000 | 122(92) | 11(8) | 0.3160 |
| 2000–2009 | 199(93–94) | 12–15(6–7) |  |
| 2010–2019 | 88(96–99) | <5(1–4) |  |
| Ever vaccinated (End of follow-up) |  |  |  |
| No | 401(94) | 27(6) | 0.4633 |
| Yes | 8(100) | 0(0) |  |
| Systemic lupus erythematosus (Index date) | |  |  |
| No | 409(94) | 27(6) | NA |
| Yes | 0(0) | 0(0) |  |
| Systemic lupus erythematosus (End of follow-up) | |  |  |
| No | 409(94) | 27(6) | NA |
| Yes | 0(0) | 0(0) |  |
| Non–AIDS defining cancer (Index date) | |  |  |
| No | 346(94) | 22(6) | 0.6657 |
| Yes | 63(93) | 5(7) |  |
| Non–AIDS defining cancer (End of follow-up) | |  |  |
| No | 259(94) | 16(6) | 0.6716 |
| Yes | 150(93) | 11(7) |  |
| Rheumatoid arthritis (Index date) | |  |  |
| No | 404(94) | 27(6) | 0.5634 |
| Yes | 5(100) | 0(0) |  |
| Rheumatoid arthritis (End of follow-up) | |  |  |
| No | 401(94) | 27(6) | 0.4633 |
| Yes | 8(100) | 0(0) |  |
| Inflammatory bowel disease (Index date) | |  |  |
| No | 409(94) | 27(6) | NA |
| Yes | 0(0) | 0(0) |  |
| Inflammatory bowel disease (End of follow-up) | |  |  |
| No | 405–408(94) | 27(6) | 0.7157 |
| Yes | <5(100) | 0(0) |  |
| Chronic obstructive pulmonary disease (Index date) | |  |  |
| No | 371(93–94) | 23–26(6–7) | 0.3245 |
| Yes | 38(90–97) | <5(3–10) |  |
| Chronic obstructive pulmonary disease (End of follow-up) | |  |  |
| No | 304(92–93) | 23–26(7–8) | 0.0896 |
| Yes | 105(96–99) | <5(1–4) |  |
| Diabetes mellitus (Index date) |  |  |  |
| No | 374(94) | 23–26(6) | 0.6487 |
| Yes | 35(90–97) | <5(3–10) |  |
| Diabetes mellitus (End of follow-up) |  |  |  |
| No | 343(95) | 19(5) | 0.0705 |
| Yes | 66(89) | 8(11) |  |
| Asthma (Index date) |  |  |  |
| No | 365(93–94) | 23–26(6–7) | 0.9543 |
| Yes | 44(92–98) | <5(2–8) |  |
| Asthma (End of follow-up) |  |  |  |
| No | 341(93–94) | 23–26(6–7) | 0.8061 |
| Yes | 68(94–99) | <5(1–6) |  |
| Chronic kidney disease (Index date) | |  |  |
| No | 388(93) | 27(7) | 0.2275 |
| Yes | 21(100) | 0(0) |  |
| Chronic kidney disease (End of follow-up) | |  |  |
| No | 312(96) | 14(4) | 0.0046 |
| Yes | 97(88) | 13(12) |  |
| Cardiovascular disease (Index date) | |  |  |
| No | 379(94) | 23–26(6) | 0.4724 |
| Yes | 30(88–97) | <5(3–12) |  |
| Cardiovascular disease (End of follow-up) | |  |  |
| No | 305(96) | 14(4) | 0.0099 |
| Yes | 104(89) | 13(11) |  |
| Substance use disorder (Index date) |  |  |  |
| No | 193(91) | 18(9) | 0.0498 |
| Yes | 216(96) | 9(4) |  |
| Substance use disorder (End of follow) |  |  |  |
| No | 129(91) | 13(9) | 0.0745 |
| Yes | 280(95) | 14(5) |  |
| Hypertension (Index date) |  |  |  |
| No | 354(94) | 22(6) | 0.4588 |
| Yes | 55(92) | 5(8) |  |
| Hypertension (End of follow-up) | |  |  |
| No | 292(96) | 12(4) | 0.0032 |
| Yes | 117(89) | 15(11) |  |
| Mood/anxiety disorders (Index date) |  |  |  |
| No | 191(93) | 14(7) | 0.6034 |
| Yes | 218(94) | 13(6) |  |
| Mood/anxiety disorders (End of follow-up) | |  |  |
| No | 149(94) | 9(6) | 0.7458 |
| Yes | 260(94) | 18(6) |  |
| Schizophrenia (Index date) | |  |  |
| No | 368(93) | 23–26(7) | 0.2810 |
| Yes | 41(91–98) | <5(2–9) |  |
| Schizophrenia (End of follow-up) |  |  |  |
| No | 355(93–94) | 23–26(6–7) | 0.3833 |
| Yes | 54(95–98) | <5(2–5) |  |
| Traumatic brain injury (Index date) | |  |  |
| No | 354(93–94) | 23–26(6–7) | 0.1427 |
| Yes | 55(93–98) | <5(2–7) |  |
| Traumatic brain injury (End of follow-up) | |  |  |
| No | 306(94) | 21(6) | 0.7307 |
| Yes | 103(94) | 6(6) |  |
| Chronic liver disease (Index date) | |  |  |
| No | 336(93–94) | 23–26(6–7) | 0.6889 |
| Yes | 73(95–99) | <5(1–5) |  |
| Chronic liver disease (End of follow-up) | |  |  |
| No | 252(94) | 15(6) | 0.5315 |
| Yes | 157(93) | 12(7) |  |
| Transplant operation (Index date) |  |  |  |
| No | 405–408(94) | 27(6) | 0.7157 |
| Yes | <5(100) | 0(0) |  |
| Transplant operation (End of follow-up) | |  |  |
| No | 399(94) | 27(6) | 0.4111 |
| Yes | 10(100) | 0(0) |  |
| **Continuous Variables** | | | |
| Age (years)(Index date) |  |  |  |
| Median | 50 | 53 | <0.0001 |
| 25^th^–75^th^ percentiles | 50–50 | 50–56 |  |
| Age (years)(End of follow-up) |  |  |  |
| Median | 58 | 67 | <0.0001 |
| 25^th^–75^th^ percentiles | 54–63 | 62–71 |  |

**Note.** NA: Not applicable; p-value significance is set at 0.05; row percentages are shown.

# **References**

1. Patterson S, Cescon A, Samji H, et al. Cohort Profile: HAART Observational Medical Evaluation and Research (HOMER) cohort. Int J Epidemiol **2015**; 44:58-67.

2. British Columbia Ministry of Health [creator]. Vital Events Deaths. V2. Population Data BC [publisher]. Data Extract. MOH(2020).

3. British Columbia Ministry of Health. BC PharmaCare plans. Available at: <https://www2.gov.bc.ca/gov/content/health/health-drug-coverage/pharmacare-for-bc-residents/who-we-cover#planc>. Accessed October 26 2022.

4. Budu MO, Kooij KW, Heath K, et al. Cohort Profile Update: Reflecting back and looking ahead: Updating the Comparative Outcomes and Service Utilization Trends (COAST) Study to include 28 years of linked data from people with and without HIV in British Columbia, Canada. Int J Popul Data Sci **2025**; 10:2496.

5. Chronic Disease Information Working Group. BC Chronic Disease and Selected Procedure Case Definitions, Case Definition – Diabetes Mellitus. Available at: <http://www.bccdc.ca/resource-gallery/Documents/Chronic-Disease-Dashboard/diabetes-mellitus.pdf>. Accessed 11-Jun-2021.

6. Williamson T, Green ME, Birtwhistle R, et al. Validating the 8 CPCSSN case definitions for chronic disease surveillance in a primary care database of electronic health records. Ann Fam Med **2014**; 12:367-72.

7. Chronic Disease Information Working Group. BC Chronic Disease and Selected Procedure Case Definitions, Case Definition – Ischemic Heart Disease. Available at: <http://www.bccdc.ca/resource-gallery/Documents/Chronic-Disease-Dashboard/ischemic-heart-disease.pdf>. Accessed 11-Jun-2021.

8. Chronic Disease Information Working Group. BC Chronic Disease and Selected Procedure Case Definitions, Case Definition – Stroke, Hospitalized Transient Ischemis Attack (TIA) Available at: <http://www.bccdc.ca/resource-gallery/Documents/Chronic-Disease-Dashboard/stroke-hospitalized-transient-ischemic-attack.pdf>. Accessed 11-Jun-2021.

9. Chronic Disease Information Working Group. BC Chronic Disease and Selected Procedure Case Definitions, Case Definition – Stroke, Hospitalized Available at: <http://www.bccdc.ca/resource-gallery/Documents/Chronic-Disease-Dashboard/stroke-hospitalized.pdf>. Accessed 11-Jun-2021.

10. Chronic Disease Information Working Group. BC Chronic Disease and Selected Procedure Case Definitions, Case Definition – Heart Failure. Available at: <http://www.bccdc.ca/resource-gallery/Documents/Chronic-Disease-Dashboard/heart-failure.pdf>. Accessed 11-Jun-2021.

11. Chronic Disease Information Working Group. BC Chronic Disease and Selected Procedure Case Definitions, Case Definition – Acute Myocardial Infarction (AMI), Hospitalized. Available at: <http://www.bccdc.ca/resource-gallery/Documents/Chronic-Disease-Dashboard/acute-myocardial-infraction-hospitalized.pdf>.

12. Chronic Disease Information Working Group. BC Chronic Disease and Selected Procedure Case Definitions, Case Definition – Mood and Anxiety Disorders. Available at: <http://www.bccdc.ca/resource-gallery/Documents/Chronic-Disease-Dashboard/mood-anxiety-disorders.pdf>. Accessed 11-Jun-2021.

13. Chronic Disease Information Working Group. BC Chronic Disease and Selected Procedure Case Definitions. Available at: <http://www.bccdc.ca/resource-gallery/Documents/Chronic-Disease-Dashboard/rheumatoid-arthritis.pdf>. Accessed 09-Jun-2023.

14. Chronic Disease Information Working Group. BC Chronic Disease and Selected Procedure Case Definitions, Case Definition – Chronic Obstructive Pulmonary Disease Available at: <http://www.bccdc.ca/resource-gallery/Documents/Chronic-Disease-Dashboard/chronic-obstructive-pulmonary-disease.pdf>. Accessed 29-Jun-2021.

15. Chronic Disease Information Working Group. BC Chronic Disease and Selected Procedure Case Definitions, Case Definition-Asthma. Available at: <http://www.bccdc.ca/resource-gallery/Documents/Chronic-Disease-Dashboard/asthma.pdf>. Accessed 09-Jun-2023.

16. Hanly JG, Thompson K, Skedgel C. Identification of patients with systemic lupus erythematosus in administrative healthcare databases. Lupus **2014**; 23:1377-82.

17. University of Manitoba. Concept: Inflammatory Bowel Disease (IBD) - Defining in Administrative Data. Available at: <http://mchp-appserv.cpe.umanitoba.ca/viewConcept.php?printer=Y&conceptID=1375>. Accessed 7 Feb 2022.

18. Chronic Disease Information Working Group. BC Chronic Disease and Selected Procedure Case Definition, Case Definition-Hypertension. Available at: <http://www.bccdc.ca/resource-gallery/Documents/Chronic-Disease-Dashboard/hypertension.pdf>. Accessed 09-Jun-2023.

19. Chronic Disease Information Working Group. BC Chronic Disease and Selected Procedure Case Definitions, Case Definition – Schizophrenia Available at: <http://www.bccdc.ca/resource-gallery/Documents/Chronic-Disease-Dashboard/schizophrenia.pdf>. Accessed 29-Jun-2021.

20. BC Ministry of Health. British Columbia Chronic Disease Registries (BCCDR) Case Definitions - Substance Use Disorder. Available at: <https://zmail.cfenet.ubc.ca/service/home/~/?auth=co&loc=en_US&id=9395&part=2>. Accessed Nov 11 2022.

21. Chronic Disease Information Working Group. BC Chronic Disease and Selected Procedure Case Definitions, Case Definition-Traumatic Brain Injury. Available at: <http://www.bccdc.ca/resource-gallery/Documents/Chronic-Disease-Dashboard/stroke-hospitalized-haemorrhagic.pdf>. Accessed 09-Jun-2023.

22. Kim DH, Lee J, Kim CA, et al. Evaluation of algorithms to identify delirium in administrative claims and drug utilization database. Pharmacoepidemiol Drug Saf **2017**; 26:945-53.

23. BC Cancer (2020): BC Cancer Registry Data. V2. Population Data BC. Data Extract. BC Cancer (2020).

24. Lapointe-Shaw L, Georgie F, Carlone D, et al. Identifying cirrhosis, decompensated cirrhosis and hepatocellular carcinoma in health administrative data: a validation study. PloS one **2018**; 13:e0201120.

25. Roberts HW, Utuama OA, Klevens M, Teshale E, Hughes E, Jiles R. The contribution of viral hepatitis to the burden of chronic liver disease in the United States. Official journal of the American College of Gastroenterology| ACG **2014**; 109:387-93.
